# Supplementary material for: Which outcomes should always be measured in intervention studies for improving work participation for people with a health problem? An international multistakeholder Delphi study to develop a core outcome set for Work participation (COS for Work)
Source: BMJ Open. 2023 Feb 15;13(2):e069174. doi: 10.1136/bmjopen-2022-069174 (PMC9933745; doi:10.1136/bmjopen-2022-069174)
Supplement: Supplementary data [file bmjopen-2022-069174supp001.pdf]

PROTOCOL

REACHING CONSENSUS ON A CORE OUTCOME SET FOR WORK PARTICIPATION. A DELPHI STUDY.

Margarita Ravinskaya, Jos H. Verbeek, Miranda W. Langendam, Ira Madan, Suzanne M.M. Verstappen, Regina Kunz, Carel T.J. Hulshof, Jan L. Hoving

Date: 21 March 2022

INTRODUCTION

Authors of systematic reviews (SRs) state that inconsistent work participation outcome reporting in clinical trials hampers evidence synthesis in the field of occupational health (1-3). Co-ordinating Editors of Cochrane review groups indicate that reliability and quality of SRs could be improved by use of core outcome sets (4). In 2019, the Coronel Institute of Occupational Health at AMC Amsterdam established an international research collaboration to launch the development of a Core Outcome Set for Work Participation (COS for Work), based on the Core Outcome Measures in Effectiveness (COMET) Trials approach (COMET Initiative | Development of a Core Outcome Set for Work Participation (COS-WP) (comet-initiative.org)

The first phase of our research project involved a systematic review on the spectrum of work outcomes used to measure the effect of interventions in published trials (5). This review showed extensive heterogeneity in work participation outcome measurements and a need for a COS for Work. In the second phase we completed a survey among systematic reviewers and trial authors to gain insight in the reasons and preferences for choosing and using work outcomes (6). In addition, we created a framework to aid the selection of outcomes on of work participation (submitted). The framework outlines four main stages of work participation which can be used to group unique outcomes relevant for each stage. The stages are: (a) initiating employment, (b) having employment, (c) increasing or maintaining productivity at work and (d) return to employment.

In the current third phase we seek consensus on a comprehensive core set of work outcomes which can be used in all intervention studies that are expected to impact work participation directly or indirectly. In the fourth and final phase we will establish a list of validated outcome instruments/ measurable definitions for each of the core work outcomes.

The overall aim of this Delphi study is to define a minimal set of outcomes that are relevant, valid and feasible for measuring the effectiveness of interventions to achieve higher work participation, based on the consensus of an international, interdisciplinary and interprofessional stakeholder group.

Ravinskaya M, *et al. BMJ Open* 2023; 13:e069174. doi: 10.1136/bmjopen-2022-069174

METHODS

Consensus process

Steps of the consensus process

The consensus process will include five steps:

1. Preparation
2. First online consensus participant discussion (20 April 2022)
3. First round of Delphi voting (11-14 May 2022)
4. Second online discussion about the results of the first Delphi round (25 May 2022)
5. Second Delphi voting round (30 May 2022)

1. Preparation

The objective of this step: prepare the participants for the first online discussion.

Two groups participate in this study:

1. The international COS steering group with researchers from four countries:

   Jan Hoving (NL) , Margarita Ravinskaya (NL), Carel Hulshof (NL), Suzanne Verstappen (UK), Ira Madan (UK), Regina Kunz (CH), Miranda Langendam (NL), Jos Verbeek (FI)

2. Study participants.

   Five types of international stakeholders will be recruited for the consensus process:

   1. Researchers in occupational health with experience in RCT/SR and / or cost-effectiveness studies
   2. Health professionals in occupational health and insurance medicine
   3. Representatives of workers (e.g. union members)
   4. Representatives of employers (e.g. members of business federations)
   5. Representatives of policy makers (e.g. government representatives, members of EU OSHA or the World Health Organization)

Participants may also fulfill multiple roles (for example be a researcher and health professional) and share their knowledge and experience as input during the discussion rounds. During the Delphi rounds, however, the participants must choose one stakeholder group that they wish to represent. We will assign the stakeholder roles to every participant based on their background and confirm prior to the first discussion round if they agree. The majority of the participants will be researchers because their experience with measuring work participation will be helpful in making informed decisions. We arranged this composition of stakeholders to provide a diversity of views from the health, societal, worker and employer perspectives. There are no set guidelines on the number of participants for the consensus on core outcome sets. A study by Signha et al. that the number of participants to range from 13 to 222 (7). We will recruit about 100 participants with the aim to have 50 participants take part in the consensus.

We will recruit potential via the following channels:

1. Invite researchers who have responded to our survey (6) and indicated that we could contact them in the future
2. Invite researchers and other stakeholders through our international network

We will identify the potential participants based on their professional background and the geographical location. Participants living in Europe, North and South America, Asia, Australia and Africa will be invited. An e-mail will be sent out specifying the aims of the project, the required input and dates of the plenary discussions and the Delphi rounds.

Using the results from the systematic review, the survey and the new framework, the steering group will write a concise background document for participants attending the two rounds of the consensus process. This includes information on the COS for Work framework, the various work participation outcomes and definitions used in the literature and our proposed definitions for the different outcomes which will be used in the Delphi voting rounds. The participants are asked to reserve approximately two hours to prepare for the consensus process by reading/familiarizing themselves with the background document. In addition they are asked to provide written feedback about any outcome which is not part of our preliminary set but they would find potentially core with their reasoning behind it.

2. First online consensus participant discussion

The objectives of the online discussion are as follows:

1. Present the COS for Work framework with the four stages of work participation: (1) initiating employment, (2) having employment, (3) increasing or maintaining productivity at work, and (4) return to employment
2. Make explicit that within each stage of work participation there are various outcome concepts and we are seeking consensus on core outcomes belonging to each category.
3. Give an example of a COS for Work: core outcomes for every stage of work participation.
4. Ensure that all participants understand their roles and their tasks for the Delphi voting
5. Discuss which are the most important outcomes from the preliminary for every group of stakeholders
6. Present any additional core outcomes we received from the stakeholders (if more than two stakeholders provided they via mail prior to the discussion)

The discussions will take two hours per group. We will divide the participants in two groups to facilitate participation across all times zones. The first group will be from 09:00 till 11:00 CET and include participants from Europe, Asia and Australia. The second group will be from 17:00 to 19:00 CET and include participants from Europe, Canada, North America and South America.

The participants will receive a Zoom link. In the e-mail invitation we will state that the session will be recorded unless anyone wishes to object. We will discuss the topics as outlined in the aims of the discussion using the background document. Participants will have the opportunity to provide input on each topic. The chair, Carel Hulshof, will facilitate the discussion and the other members of the steering group will facilitate the chat and the poll functions. The chair will work with a meeting

manual prepared by the steering group. The results of this online discussion will serve as input for the first Delphi round.

3. First round of Delphi voting

Objective: get a preliminary agreed upon set of core outcomes for each stage of work participation

If for some reason participants were not able to attend the first discussion round they may take part in the Delphi voting if they familiarized themselves with the background document. Any written feedback will be taken into consideration by the steering group as input for the Delphi voting.

The list of the outcomes for every stage of work participation will be agreed upon during the discussion. If more than one stakeholder wants to add an outcome which was not part of the preliminary set we will add the outcome to the Delphi rounds.

Voting procedures and interpretation of the findings:

Participants will receive a link to register as a stakeholder for the DelphiManager software (provided by COMET) and vote on which categories and outcomes within the four stages of work participation they deem as core outcomes.

Quantitative data will be collected and provided to participants to inform the ranking of outcomes. The DelphiManager includes ranking based on a Likert Scale from 1-9; from not important to critical. An “unable to score” option is also given.

Consensus on outcomes to include in the core outcome set is when ≥ 80% of participants score an outcome as critically important (score 7 to 9) and consensus to not include an outcome as core is when <50 % of participants score outcomes with 7-9. However, it is possible that too many outcomes may have consensus to include for a core outcome set and some important outcomes may receive low scores due to lack of awareness of their importance by the majority of stakeholders. Therefore we will use the consensus definition, as described above, as a guideline and provide the participants with the opportunity to elaborate on their choices during the second discussion.

4. Online discussion of the results of the first Delphi round

The aim of the second discussion will be to go through the results of the first voting round. Outcomes with most agreement (score 7 to 9) will be presented and discussed. Clarifications of the outcomes and opinions on why certain outcomes deserve priority will be discussed. Any strong arguments from stakeholders against high scoring outcomes and potential “downgrading” of high scoring outcomes will be discussed. Outcomes which were not ranked as critical (<50 % of participants score outcomes with 7-9) will also be discussed and the participants will provide input whether all or some these outcomes can be dismissed from the second round of voting. Based on this discussion the steering group will consider the argumentation of the stakeholders and decide which outcomes will be included in the second Delphi round.

5. Second Delphi voting round

The objective of the second Delphi round will be to reach agreement by stakeholders on the final set of core outcomes for each stage of work participation. In the second Delphi round the participants will see the scores from other participants on every outcome within each of the four stages and re-rate the outcomes which were considered critical after the first round of voting and the second discussion round.

DATA ANALYSIS

The data from the Delphi rounds will be processed anonymously but in the discussion rounds the participants will be identifiable.

Group discussions

We will summarize the main take-aways from the group discussion on every topic and hold a meeting with the steering group on how we will process the input for the Delphi study. For the topics which require voting, at least 70% agreement will be required for an argument to be considered as a "yes".

Delphi voting rounds

The results from the Delphi rounds will be extracted from the Delphi Manager software into a CSV format and analyzed in SPSS using descriptive statistics. We will report measures of the distribution scores for all outcomes which will be included in the final round. If too many outcomes will have agreement on inclusion, outcomes with the highest mean scores within each stage of work participation will be selected as core.

RESULTS

Results will be processed anonymously. Qualitative data from the discussions with the stakeholders will be summarized. Quantitative data from the voting rounds will include distributions of the scores for all outcomes and the number of participants who scored the outcomes.
In the following project the steering group will give recommendations on how to best measure the selected core outcomes. The results will be reported in an article to be submitted to an international peer reviewed journal. After the publication we will share the results with the participants and disseminate further within the research community.

Reference list

1. Hoving JL, Lacaille D, Urquhart DM, Hannu TJ, Sluiter JK, Frings-Dresen MH. Non-pharmacological interventions for preventing job loss in workers with inflammatory arthritis. Cochrane Database of Systematic Reviews. 2014(11).
2. de Boer AG, Taskila TK, Tamminga SJ, Feuerstein M, Frings-Dresen MH, Verbeek JH. Interventions to enhance return-to-work for cancer patients. Cochrane database of systematic reviews. 2015(9).
3. Nieuwenhuijsen K, Faber B, Verbeek JH, Neumeyer-Gromen A, Hees HL, Verhoeven AC, et al. Interventions to improve return to work in depressed people. Cochrane Database of Systematic Reviews. 2014(12).
4. Kirkham JJ, Gargon E, Clarke M, Williamson PR. Can a core outcome set improve the quality of systematic reviews?–a survey of the Co-ordinating Editors of Cochrane Review Groups. Trials. 2013;14(1):21.
5. Ravinskaya M, Verbeek JH, Langendam M, Daams JG, Hulshof CT, Madan I, et al. Extensive variability of work participation outcomes measured in randomized controlled trials: a systematic review. Journal of Clinical Epidemiology. 2022;142:60-99.
6. Ravinskaya Margarita; Verbeek JHL, Miranda; Hulshof, Carel TJ; Madan, Ira; Verstappen, Suzanne MM; Kunz, Regina; Hoving, Jan L. Preferred methods of measuring work participation. An international survey among trialists and Cochrane systematic reviewers. "In press". 2022.
7. Sinha IP, Smyth RL, Williamson PR. Using the Delphi technique to determine which outcomes to measure in clinical trials: recommendations for the future based on a systematic review of existing studies. PLoS medicine. 2011;8(1):e1000393.
