# Supplementary material for: Which outcomes should always be measured in intervention studies for improving work participation for people with a health problem? An international multistakeholder Delphi study to develop a core outcome set for Work participation (COS for Work)
Source: BMJ Open. 2023 Feb 15;13(2):e069174. doi: 10.1136/bmjopen-2022-069174 (PMC9933745; doi:10.1136/bmjopen-2022-069174)
Supplement: Supplementary data [file bmjopen-2022-069174supp002.pdf]

Development of a core outcome set for work participation:

COS for Work

Introduction on COS for Work and instructions for the Delphi study

Steering group

Dr. Jan L. Hoving - Amsterdam UMC, location Academic Medical Center, University of Amsterdam, Coronel Institute of Occupational Health, Amsterdam Public Health research institute, Amsterdam, NL

Margarita Ravinskaya - Amsterdam UMC, Academic Medical Center, University of Amsterdam, Coronel Institute of Occupational Health, Amsterdam Public Health research institute, Amsterdam, NL

Dr. Jos H. Verbeek MD - Amsterdam UMC, location Academic Medical Center, University of Amsterdam, Coronel Institute of Occupational Health, Amsterdam Public Health research institute, Amsterdam, NL

Dr. Miranda W. Langendam - Amsterdam UMC, location Academic Medical Center, University of Amsterdam, Department Epidemiology and Data Science, Amsterdam Public Health research institute, NL

Prof. Ira Madan, MD, FRCP, FFOM – Guy's and St Thomas' NHS Trust and Faculty of Life Sciences and Medicine, King's College London, Centre for Musculoskeletal Health and Work, UK

Prof. Dr. Regina Kunz, MD - Research Unit EbIM, Evidence Based Insurance Medicine, Department of Clinical Research, University of Basel, CH

Dr. Suzanne M.M. Verstappen - [1] Centre for Epidemiology Versus Arthritis, Faculty of Biology, Medicine and Health, The University of Manchester, UK, [2] NIHR Manchester Biomedical Research Centre, Manchester University NHS Foundation Trust, Manchester Academic Health Science Centre, UK, [3] MRC Versus Arthritis Centre for Musculoskeletal Health and Work, University of Southampton, UK

Prof. Dr. Carel T.J. Hulshof, MD - Amsterdam UMC, location Academic Medical Center, University of Amsterdam, Coronel Institute of Occupational Health, Amsterdam Public Health research institute, Amsterdam, NL

1. Introduction

1.1 What is this document about?

You have accepted our invitation to take part in the consensus process to establish a core outcome set for work participation (COS for Work). Thank you for taking the time to collaborate with all of our international colleagues in the field of occupational health and related areas. In this document we explain the need for and purpose of COS for Work, how the consensus process will work, and what is expected of you.

1.2 What is a COS?

A COS is an agreed *minimum* set of outcomes that should be measured consistently in all studies in a specific health area to make study findings on similar topics more comparable. These core outcomes are chosen and defined by key stakeholders, for example health professionals, researchers, patient population and policy makers.

Core outcome sets have the following benefits: (1) facilitate large scale evidence synthesis by systematic reviews, (2) provide clearer answers to the question of what works best, (3) support informed decision making in clinical practice and policy, (4) reduce reporting bias and research waste, and (5) ensure that the outcome

measures selected are truly critical to key stakeholders[1] . A common misconception about the use of core outcome sets is that studies should *only* include the outcomes from the COS. This is not the case: rather, any other primary and secondary outcomes can be measured *alongside* the outcomes from a specific COS.

The current heterogeneity in measuring work participation (WP) hinders a full understanding of the findings across similar studies. A small set of WP outcomes used in all studies would close this gap. As of date such a generic set of study outcomes is lacking. This project aims to create a COS for Work. We consider this project to be a highly relevant contribution to research methodology of occupational health. It is a starting point. Further iterations will refine and improve the COS over time.

1.3 The purpose COS for Work

Even though COS can be developed for practice and/or research, this COS for Work is intended for research purposes only. The aim is to assist researchers in choosing which outcomes to measure in effectiveness studies to improve WP for people with health problems. Our COS for Work will be a generic COS (in contrast to COS that are developed for a specific disease). This means, it intends to evaluate any type of WP intervention in people with any type of health problem.

In general, two types of interventions can be distinguished: (1) vocational interventions with direct impact on work participation, such as Individual Placement and Support studies, and (2) non-vocational interventions, i.e. any type of clinical, pharmacological, psychological study with an intervention to improve health. For non-vocational interventions the effect on work participation is often a secondary objective and work outcomes are measured as secondary outcomes.

1.4 Our preparatory studies for COS for Work

To lay the groundwork for the consensus process, we conducted three studies. The full text versions are attached to the e-mail. Reading is optional but highly recommended. Below is a summary of the aims and findings of the three papers.

1. **Extensive variability of work participation outcomes measured in randomized controlled trials: a systematic review. https://doi.org/10.1016/j.jclinepi.2021.10.013 – Published**
We performed a systematic review which showed how WP outcomes are measured across randomized controlled trials (RCTs) aimed to impact WP directly or indirectly. We found a great deal of heterogeneity on the level of outcome terminology and measurement methods. We grouped outcomes in four main categories: “employment status”, “absence from work”, “at work productivity loss” and “employability”.

2. **Preferred methods of measuring work participation. An international survey among trialists and Cochrane systematic reviewers. https://doi.org/10.1007/s10926-022-10031-0 - Published**
We contacted the authors of the RCTs included in the above review and the authors of Cochrane systematic reviews to explore their reasons for choosing specific outcomes and measurement methods. Our findings show that the authors based their choice of measurement methods mainly on previous similar studies, that feasibility is the most important factor in the choice of measurement method, and that a COS for Work is considered very useful for research. In addition, the authors indicated that the most important outcomes for a COS for Work would be “employment status” and “sickness absence” and “return to work”. Their preferred follow-up period was 12 months across all outcome categories.

[1] Williamson PR, Altman DG, Bagley H, Barnes KL, Blazeby JM, Brookes ST, et al. The COMET handbook: version 1.0. Trials. 2017;18(3):280.

3. **A general framework for selecting work participation outcomes in intervention studies among persons with health problems.** **https://doi.org/10.21203/rs.3.rs-1342930/v1 - Under review**
As work participation is an umbrella term containing different types of outcomes, we looked for a comprehensive framework that could be used for COS for Work. Since there was no pre-existing framework, we developed one for COS for Work. The framework includes the following four stages of WP: (1) initiating employment, (2) having employment, (3) increasing or maintaining productivity at work and (4) return to employment. These work participation stages should help our consensus process to select the outcome concepts and outcome measures. Furthermore, we defined criteria for COS outcomes that are part of the document for selecting outcomes in the Delphi study.

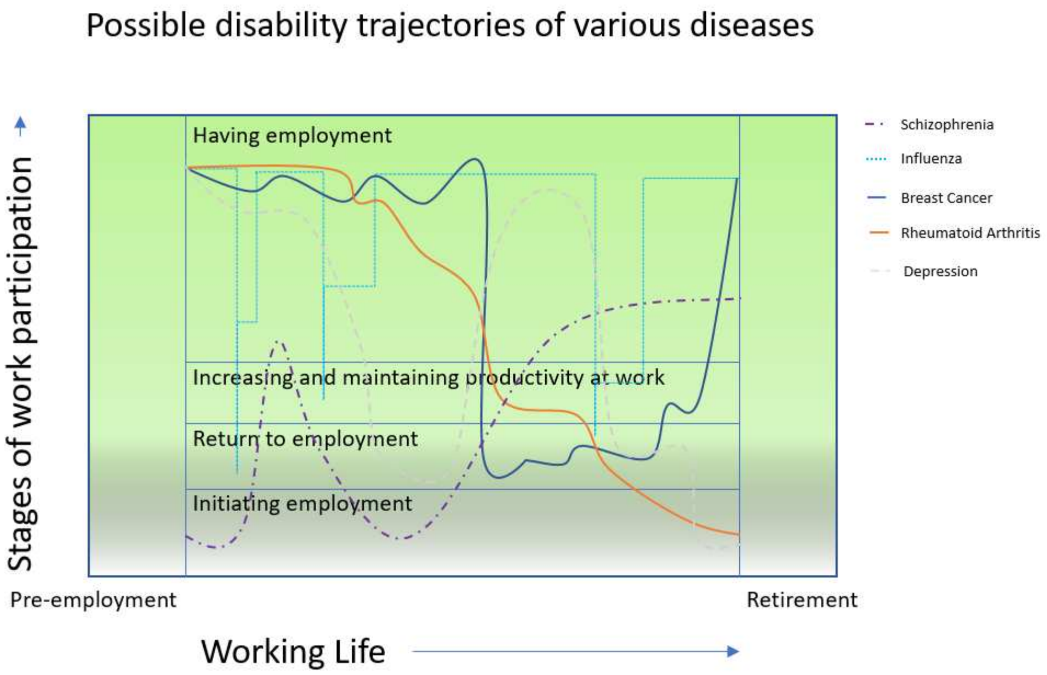

This figure is adapted from the COS framework. Here we illustrate how possible disability trajectories result from various health problems. The four work participation stages can be experienced during the course of the working life.

When linking the 24 commonly used outcomes to the four stages, 10 outcomes referred to 'Return to employment', 5 outcomes to 'Increasing or maintaining productivity at work', 7 outcomes to 'Having employment' and 2 outcomes to 'Initiating employment'. Many of these outcomes can be refined in more details, e.g. by specifying the timing (1 day, 4 weeks, 12 months) or type or amount of work (full time, part-time, last job, any work).

2. Instructions for the Delphi Study

2.1 Purpose of the study

The purpose of this consensus study is to identify a core set of outcomes (COS) that captures work participation and that can be used in intervention studies at each of the four stages of work participation

identified in our framework. Please note that discussion and votes in this study do not address measurement instruments.

The study includes 4 activities: Two online consensus discussions and two rounds of Delphi voting.

1. First online group discussion (20 April 2022)
2. Delphi I voting (11 – 14 May 2022)
3. Second online group discussion about the results of the first Delphi vote (25 May 2022)
4. Delphi II voting (30 May – 1 June)

We seek a minimum set of outcomes that are meaningful and easy to measure ('feasible') by all studies in the field. There are no strict rules for the number of outcomes a COS may contain, but the current 24 outcomes would defeat the purpose. Compromises may need to be made in the number and type of outcomes.

2.2 Organization

a) Selection of participants

To cover core outcomes relevant to a broad range of stakeholders, we invited experts from diverse professional groups. We have assigned you to one of the following stakeholder groups: researcher, policy maker, employer representative, employee representative, occupational health professional (see document "COS Delphi participants_ stakeholder groups"). Please use the perspective of your assigned stakeholder group in your country when discussing and selecting the core outcomes. Some of you fulfill more than one role in practice or have done so in the past. If for any reason you disagree with the group you have been assigned to, please let us know.

b) Running the meeting

Chair: Prof. Carel Hulshof will chair the meetings, and the COS for Work team will assist the process.

Protocol: To be transparent on how consensus is reached, we are working with a protocol that is publicly available on the COMET website (https://www.comet-initiative.org/studies/details/1195).

Video recording: We will record the online meetings for study purposes only. The recordings will be destroyed before 31 December 2022.

c) Ground rules for communication

All stakeholders should have the opportunity to voice their opinion. As the time for discussion will be limited, please try to present your arguments briefly and succinctly so that others can also put forward their arguments, thus facilitating the exchange.

d) Contact for queries

If anything is unclear, please do not hesitate to contact us.

Contact: Margarita Ravinskaya, m.ravinskaya@amsterdamumc.nl

2.3 The activities

1. First online group discussion

When: 20 April 2022

Zoom link

Connect to the discussion via the following link: https://uva-live.zoom.us/j/9294218837

Aims of the session

1. Ensure that all participants understand their roles and their tasks for the discussions and the Delphi votes.
2. Present the COS for Work framework.
3. Make explicit that each stage of work participation has more than one outcome concept. We are seeking consensus on outcome concepts and the core outcomes belonging to each stage for most stakeholders.
4. Present additional core outcomes that may have been submitted by at least two stakeholders.
5. Discuss the most important outcomes from the preliminary outcome set (document "Delphi voting: Stages and Outcomes"), first in breakout rooms and finally in plenary setting.

Preparatory work for the participants

1. Familiarize yourself with the document "Delphi voting: Stages and Outcomes" (see email attachment) to be used for the Delphi rounds. The preliminary list of outcomes per WP stage are based on the outcomes most commonly measured in our systematic review (see email attachment). If you miss crucial outcomes, please send us those outcomes and your reasons, as soon as possible but 18 April 2022 at the latest.
2. Write your preliminary selection of core outcomes in the worksheet table in the document. Please specify the minimum of outcomes that you think should be included in COS for Work.

   In this project, we aim to achieve consensus on outcomes only (not on measurement instruments). While choosing your preferred core outcomes, please write down your rationale for doing so. For example, in our survey reasons for choosing a certain outcome were their use in similar studies, relevance for the study population, or easy access to registry data. Discussing and balancing preferences for specific outcomes will facilitate the final selection.

**Definition "Outcome Measure" versus "Measurement Instrument"**		
Please note the difference between "outcome concept", "outcome measure" and "measurement instrument" (or tool or method). "Outcome concept" is an umbrella term for the many facets of a certain concept. *An outcome is WHAT we aim to measure. A measurement instrument is the method HOW the outcome is measured.*<br>The Delphi voting will refer to the colored columns only (NO VOTING on the measurement instrument)		
Outcome concept	Outcome measure<br>(Description)	Measurement instrument<br>(Example)
Work ability	Current self-rated physical and mental work ability compared to lifetime best	The Work Ability Index

3. Recommended but optional: familiarize yourself with our three background studies (systematic review, survey, and framework, see email attachment).

2. Delphi I: Individual online voting

When: 11 – 14 May 2022

Individual assignment for all participants

Use the DelphiManager software to select and rank outcomes and to motivate your selection and ranking. Please complete this task between 11 and 14 of May 2022.

Overall group aim:

- Reach an initial consensus on a set of core outcomes for each stage of work participation.

The DelphiManager software

We will use the DelphiManager software to vote on the outcomes for the four stages of WP. You will receive a link to register for the DelphiManager software.

The DelphiManager includes a ranking of the outcomes on a Likert Scale from 1-9: from 'not important' to 'critical', plus an 'unable to score' option. The results will be summarized and reflected in the second discussion round and a refined set of outcomes will be presented in the second Delphi vote.

3. Second online group discussion

When: 25 May 2022

Zoom link

Connect to the discussion via the following link: https://uva-live.zoom.us/j/9294218837

Overall group aim:

- Discuss the results of the first Delphi voting round with other stakeholders.

We will discuss the outcomes with the highest agreement (score 7 to 9) and the rationale on why certain outcomes deserve priority. Any strong arguments from stakeholders against high scoring outcomes and potential "downgrading" of high scoring outcomes will be discussed. Outcomes not ranked as critical (<50 % of the participants scored the outcomes with 7-9) will be reviewed to determine whether some or all of these outcomes can be dismissed from the second round of voting. Based on the arguments of the stakeholders in this discussion, the COS for Work team will make the final decision on which outcomes to include in the second Delphi vote.

4. Delphi II : Individual online voting

When: 30 May – 1 June 2022

Overall group aim:

- Reach consensus on the final set of core outcomes for each stage of work participation.

In the second Delphi voting round, participants see the scores of the other participants for each outcome in all four stages. Using DelphiManager software, you will re-rate all outcomes that were considered critical after the first Delphi round of voting and the second round of discussion.
