# Supplementary material for: Which outcomes should always be measured in intervention studies for improving work participation for people with a health problem? An international multistakeholder Delphi study to develop a core outcome set for Work participation (COS for Work)
Source: BMJ Open. 2023 Feb 15;13(2):e069174. doi: 10.1136/bmjopen-2022-069174 (PMC9933745; doi:10.1136/bmjopen-2022-069174)
Supplement: Supplementary data [file bmjopen-2022-069174supp003.pdf]

Introduction

In this document we present a list of work participation outcomes measured randomized controlled trials. The outcomes are sorted according to the stage of work participation.
The stages are presented in the order of most to least commonly measured outcomes.

First, you will find a brief explanation of every stage. In particular we describe for which target group and intervention types outcomes belonging to each stage are relevant. We also provide some additional information for every stage. The “criteria to consider for COS outcomes” were developed as part of the framework. They are mostly important on the level of measurement. We will use these criteria after the Delphi study to determine how each COS outcome resulting from this Delphi study can be measured. We include the criteria for your information, so you may take them into consideration (as far as possible) when selecting most important outcomes.

For every stage you will find a preliminary set of outcomes. The “outcome concept” refers to a category of outcomes within each stage. The “outcome measure” is the list of outcomes which will be used to vote on in the Delphi rounds. In addition, we provide examples of measurement tools, instruments or methods.

Please consider which outcome measures you find most important and complete the worksheet belonging to every stage. It will be helpful if you also note the outcome ID you are referring to. This will be the basis of our first round of Discussion on 20 April 20 2022.

If you miss an important outcome concept or outcome measure, please send us the outcome and the reason of its’ importance. **Please do so 18 April 2022 at the latest**, in that way we can include the additional outcomes in the first discussion on 20 April.

Stage ‘Return to Employment’ & Sick leave from work

This is a stage of work participation when employed people (temporarily) stop attending work and are on sick leave. The ICF does not include sick leave or sickness absence, but potentially outcomes for this stage could fit under “maintaining employment” and “remunerative employment” ICF categories. Work related rehabilitation interventions designed to help workers return to work include outcomes relevant to this stage as their primary outcome. Any type of clinical, pharmaceutical or otherwise health related intervention may be expected to indirectly impact sick leave. Sickness absence can show that a person is unable to fulfil their worker role due to ill health, but other reasons for absenteeism, such as maternity leave or unwillingness to come to work also exist. Evaluation of absenteeism outcomes is often approached from the societal/economic perspective. Sick leave is often converted into a monetary value as part of economic evaluations to indicate extra costs for the employer or/and the employee.
Measuring changes in sick leave is not straightforward as the denominator *can refer to all workers or to workers that are on sick leave*. It is, therefore, very important to be clear to which group the sick leave figures refer.
More recently, the term ‘return to work’ has been introduced as an indicator of sickness absence from an individual perspective. For individuals, the worker role is important for economic reasons but also for reasons of well-being. For example, cancer survivors report that getting back to work is a final step in getting back to normal life after their disease/treatment experience.

Additional information to consider for this stage

- Outcomes within this stage of work participation are measured most frequently (45% of all RTC’s in our review) in intervention studies which directly or indirectly aim to impact work participation
- In our survey, 93% of RCT authors and 69% of Cochrane systematic reviewers indicated that outcomes belonging to this stage should be in COS for Work
- Registry data is preferred by researchers, but type and definition of sickness benefits registry data varies per country and may be inaccurate about the diagnosis

- Time to RtW as a standalone measure may not be a meaningful outcome about sustained effect of an intervention (recommendations are made to also measure duration of sick leave, sick leave spells and proportion of employees who return to part/full time work)

Criteria for COS outcomes

Criteria to consider	***Optional criteria***
Outcomes should:<br>1. Be sensitive to change<br>2. Be feasible to measure<br>3. Be applicable internationally<br>4. Be work participation specific<br>5. Capture multiple stakeholder perspectives<br>6. Be in alignment with the ICF model	*Outcomes should:*<br>1. Be used for cost-effectiveness studies<br>2. Be applicable across varying insurance schemes

**ID**	**Outcome concept**	**Outcome measure (description)**	**Measurement instrument (examples)**
1.1.1	Return to work	*Proportion of workers that return to work* after being absent because of an illness	- to any type of work<br>- to specific type of work<br>- to a defined amount of work<br>- at a defined point in time (e.g. at least 1 day, 3 days, 4 weeks, 12 months)
1.1.2		*Time to return to work* or duration of sickleave after being absent because of an illness	- to any type of work<br>- to specific type of work<br>- to a defined amount of work<br>- at a defined point in time (e.g. at least 1 day, 3 days, 4 weeks, 12 months)
1.1.3	Sustainable Return to work	*Proportion of workers that return to work and remain at work for a specified amount of time*	e.g., 4 weeks without relapse of sick leave at a specific moment of follow-up e.g. 12 months
1.1.4		*Time to return to work in workers absent because of an illness and who do not relapse within a specified amount of time*	e.g., 4 weeks at a specific moment of follow-up e.g. 12 months
1.1.5	Return to work: the workers perceptions	*Time it will take to return to work as perceived by the sick worker;* also described as self-efficacy for return to work, return to work expectations or intention to return to work	- Work Readiness Questionnaire (WoRQ)<br>- Inventory for Job Coping and Return Intention (JoCoRi)

1.2.1	Sickness absence *among all workers* in an organization/group	*A. Lost working days*<br>*B. Lost sick days*	A. Percentage of all working days of all workers in an organization during a time period e.g. 12 months<br>B. Sick days as percentage of all available days during a time period
1.2.2		*Sick leave duration*	- Working days absent because of illness averaged overall workers<br>- Calendar days sick
1.2.3		*Sick leave frequency*	Number of episodes of sick leave per worker expressed as percentage

1.2.4	Sickness absence *among absent workers* in an organization/group	*a) Lost working days*<br>*or*<br>*b) lost sick days*	a) percentage of all working days during a time period e.g. 12 months;<br>b) sick days as a percentage of all available days during a time period
1.2.5		*Sick leave duration*	• working days absent averaged over all sick leave episodes; can also be expressed as calendar days sick;<br>• is numerically identical to *time to return to work* among workers absent from work

Worksheet for participants

**Outcomes measures for 'Return to Employment'**	**Reasons for selecting these outcomes**<br>*(Examples: previously used in similar studies, relevance for the study population, easy access to data, e.g. registry data, ...)*
**Step 1: Select all outcomes that you feel are important**	
a.	
b.	
c.	
d.	
e.	
f.	
g.	
**Step 2: Note the outcomes that you consider to be the bare minimum for COS for Work**	
a.	
b.	
c.	
d.	
e.	
f.	
g.	

Stage 'Increasing or maintaining productivity at work'

This stage of work participation occurs when people experience limitations or impairments whilst working which results in lower quality or lower output of work. The input versus output is often used to calculate cost. In terms of ICF, the outcomes could be placed under the ICF-category "Maintaining a job".
Outcomes measure relevant for this stage are for any type of vocational or clinical/pharmaceutical intervention that may impact on overall productivity, work ability and work functioning of people with a health problem.
From the worker perspective, feeling fit to work is essential for a successful worklife. Employers can offer interventions to reduce stress and increase wellbeing in the workplace as preventive measures.

Additional information to consider for this stage

- Outcomes for this stage were measured second most frequently in the review; 30% of all RCTs measured an outcome relating to productivity.
- 63% of the RCT authors and 75% of Cochrane reviewers indicated in the survey that measuring outcomes for this stage should be part of the core outcome set.

Criteria for COS outcomes

Criteria to consider	***Optional criteria***
Outcomes should:<br>1. Be sensitive to change<br>2. Be feasible to measure<br>3. Be applicable internationally<br>4. Be work participation specific<br>5. Capture multiple stakeholder perspectives<br>6. Be in alignment with the ICF model	*Outcomes should:*<br>1. Be used for cost-effectiveness studies<br>2. Be applicable across varying insurance schemes

**ID**	**Outcome concept**	**Outcome measure (Description)**	**Measurement instrument (Examples)**
2.1.1	Work productivity loss (economic evaluation)	Overall loss of productivity at work (in terms of quality or output at work) resulting from presenteeism or absenteeism regardless of cause.<br>Typically used for economic evaluations	Work Productivity and Activity Impairment Questionnaire
2.2.1	Presenteeism	Not being productive while at work due to health problems.<br>Typically used to evaluate the effect of health interventions for individuals	Stanford Presenteeism Scale
2.3.1	Work ability	Current self-rated physical and mental work ability compared to lifetime best. Often used for prognosis of RtW, however the commonly used tool (WAI) is not effective for identifying changes of work ability after RtW	Work Ability Index (WAI)
2.4.1	Work activity impairment	Experienced functional impairment of performing work activities due to a health problem.	Work Limitations Questionnaire (e.g. WLQ 25)
2.5.1	Perceptions affecting productivity	Beliefs in working capacity, motivation for work, vocational commitment, job coping, contentment with work, need for recovery	- Subjective Prognosis of Work Capacity<br>- Work Motivation Scale<br>- Need for Recovery Scale<br>- Job Coping (JoCoRi))

Worksheet for participants

**Outcomes measures for 'Increasing or maintaining productivity at work**	**Reasons for selecting these outcomes** *(Examples: previously used in similar studies, relevance for the study population, easy access to data, e.g. registry data, ...)*
**Step 1: Select all outcomes that you feel are important**	
a.	
b.	
c.	
d.	
e.	
f.	
g.	
**Step 2: Note the outcomes that you consider to be the bare minimum for COS for Work**	
a.	
b.	
c.	
d.	
e.	
f.	
g.	

Stage 'Having employment'

Outcomes relevant for this stage of work participation measure whether a person is in employment, can retain employment, loses work or retires within the duration of the study. In general, these outcomes fit in the ICF categories under "Remunerative Employment" and "Keeping a Job". Being employed can be considered as having a contract with an employer or being self-employed. Both, vocational or non-vocational interventions, are relevant for this stage. Most studies on vocational rehabilitation have the primary aim to determine the effect of an intervention on employment status. Medical or pharmaceutical studies are more likely to measure the effect of the intervention on employment status as secondary outcome. The target group of these interventions are people who are known to be at risk of losing employment due to a health problem.

Additional information to consider for this stage

- Our review showed that 16% of all measured outcomes belonged to this stage.

- 98% of RCT authors and 100% of Cochrane reviewers agreed that outcomes for this stage of work participation should be part of COS for Work.
- Most of the measurement tools used to measure outcomes at this stage are not validated, such as unspecified (self-developed) items, interviews, and phone calls. The exception is data from registries such as those in the Nordic countries.

Criteria for COS outcomes

Criteria to consider	***Optional criteria***
Outcomes should:<br>1. Be sensitive to change<br>2. Be feasible to measure<br>3. Be applicable internationally<br>4. Be work participation specific<br>5. Capture multiple stakeholder perspectives<br>6. Be in alignment with the ICF model	*Outcomes should:*<br>1. Be used for cost-effectiveness studies<br>2. Be applicable across varying insurance schemes

**ID**	**Outcome concept**	**Outcome measure (Description)**	**Measurement instrument (Examples)**
3.1.1	Employment	Having any type of employment including self-employment	
3.1.2	Employment	Having met a pre-defined status of employment; part/full time, competitive work in a mainstream setting, performing the same tasks as non-disabled workers	
3.1.3	Employment	Predefined type or amount of income	
3.1.4	Employment	Duration of employment	
3.2.1	Employment	Having lost employment	
3.3.1	Work disability	Permanent and complete inability to engage in any work participation	
3.3.2	Work disability	Permanent partial disability to engage in work participation	

Worksheet for participants

**Outcomes measures for 'Having employment'**	**Reasons for selecting these outcomes**<br>*(Examples: previously used in similar studies, relevance for the study population, easy access to data, e.g. registry data, ...)*
**Step 1: Select all outcomes that you feel are important**	
a.	

b.	
c.	
d.	
e.	
f.	
g.	
**Step 2: Note the outcomes that you consider to be the bare minimum for COS for Work**	
a.	
b.	
c.	
d.	
e.	
f.	
g.	

Stage: Initiating employment

Outcomes within this stage measure whether participants are prepared for initiating employment. The target group are people who are unemployed and seeking employment (i.e., not self-employed or contracted by an employer, however, possibly with a type of subsidized governmental wage replacement benefit). This stage is particularly applicable to those who never worked, for example due to just completed education. ICF categories “Apprenticeship” (work preparation) and “Acquiring a job” apply. Interventions for this category focus on increasing skills, knowledge or attitude of participants for successful engagement in a workers’ role.

Additional information to consider for this stage

- The outcomes belonging to this stage were measured least frequently in our review, in about 5% of all trials.
- In our survey, 59% of RCT authors and 75% of Cochrane reviewers indicated that outcomes from this category should be part of the COS.
- These outcomes have been shown to be a predictor for employment status.

Criteria for COS outcomes

Criteria to consider	***Optional criteria***
Outcomes should:	*Outcomes should:*

Criteria to consider	*Optional criteria*
7. Be sensitive to change<br>1. Be feasible to measure<br>2. Be applicable internationally<br>3. Be work participation specific<br>4. Capture multiple stakeholder perspectives<br>5. Be in alignment with the ICF model	1. Be used for cost-effectiveness studies<br>2. Be applicable across varying insurance schemes

ID	Outcome concept	Outcome measure (Description)	Measurement instrument (Examples)
4.1	Skills for job procurement	Total work-related network, Engagement in work seeking activities, Job interview skills, Work-related benefit of vocational training	- Work section of the Social Adjustment scale<br>- Mock Interview Rating Scale
4.2	Self-efficacy	Self-efficacy for job procurement, Hope, optimism and self-efficacy for achieving vocational success, Social/interpersonal self-efficacy	- Job Procurement Self-efficacy Scale<br>- The Work Hope Scale<br>- Subjective Prognosis of Work Capacity

Worksheet for participants

Outcomes measures for 'Initiating employment'	Reasons for selecting these outcomes<br>*(Examples: previously used in similar studies, relevance for the study population, easy access to data, e.g. registry data, ...)*
**Step 1: Select all outcomes that you feel are important to include in COS for Work**	
a.	
b.	
c.	
d.	
e.	
f.	
g.	
**Step 2: Note the outcomes that you consider to be the bare minimum for COS for Work**	
a.	
b.	

c.	
d.	
e.	
f.	
g.	
