# Supplementary material for: Which outcomes should always be measured in intervention studies for improving work participation for people with a health problem? An international multistakeholder Delphi study to develop a core outcome set for Work participation (COS for Work)
Source: BMJ Open. 2023 Feb 15;13(2):e069174. doi: 10.1136/bmjopen-2022-069174 (PMC9933745; doi:10.1136/bmjopen-2022-069174)
Supplement: Supplementary data [file bmjopen-2022-069174supp004.pdf]

Suggested outcome(s)	Stage	Argumentation	Other remarks
**“Work environment” Availability/access to appropriate work modifications/supports**	Increasing or maintaining productivity	This is an issue that we have identified at least for physical disability groups that we have surveyed/interviewed. This also ensures a ‘biopsychosocial approach’ – in terms of CoS identifying personal factors but also factors in the social environment that are critical to RTW.	
**Work-life balance**	Increasing or maintaining productivity	We notice in people with Parkinson’s they initially keep up productivity at work at cost of participation in other life roles. You partly measure this with ‘need for recovery’… But would you consider including a measure about satisfaction with participation (e.g. USER-P- satisfaction scale?)	
**Additional perceptions affecting productivity**	Increasing or maintaining productivity	Are (self)-perceived stigma and perceived sef-efficacy at work covered in the concepts you describe?	
			A general problem I have with a lot of the current PROMS: they all assume the person is employed; some questions are not appropriate for people who are self-employed.
**Perceived competences**	Unclear		
**Impact of the disease on the career**	Unclear		
			The first question is that we probably should define the scope : individual level (e.g. effect of an intervention for people with cancer or depression) of collective level (effect of preventive measures on the rate of sick leave in a company),

			in order to priorize the COS
			The contentment for the job is mentioned but there is no COS for this
			Work ability index : effect of the disease or of aging ? We should include age in the analysis since the WAI may decrease with age, independently from the potential onset of diseases
**Predefined type of occupation**	Having employment	Sometimes people want to work in an occupation that meets their values more than money or time.	
**Predefined commute time**	Having employment	Work is a social activity for humans in the COMMUNITY. In some countries, it can take a long time to get to the workplace. Also, with regard to mental illness, people may be hesitant to work near their homes due to stigma. Therefore, matching commuting time seems to be important.	
**Predefined illness disclosure status**	Having employment	It would be important to make your own decisions about disclosure or nondisclosure for mental illness, HIV/AIDS, LGBT, and other conditions that are highly stigmatized.	
**Job satisfaction**	Having employment	Ultimately, job satisfaction appears to be most important, while measuring this outcome needs further discussion and scientific evidence.	
Lost working days <br>**(please note: I propose instead a slightly different measure: *working time of observed time ~ working days of days lived/observed*)**	Return to Employment	Basically, this measurement is sensitive to almost all core aspects of most other proposed measurements: time to RtW, sustained RtW and	

		risk of reoccurrence of sick leave:<br><br>It is sensitive to both sick leave duration of index sick leave *and* any reoccurrence of sick leave as well, and the length and timing of both of these events. This is contrary to *time to RtW (1.1.2), sick leave duration (1.2.5)* that are not sensitive to reoccurrence and *time to RtW… (1.1.4)* that is not sensitive to relapse beyond the specified threshold period (e.g. 4 weeks).	
Time to return to sustainable work, with high threshold (e.g. four consecutive months)	Return to Employment	Time to return to work (or any other positive event) is often not sensitive to reoccurrence – this problem can be handled by defining the threshold of sustainability rather high, like e.g. four months. The threshold of sustainability should be high enough, so that any reoccurrence after this threshold would not change the perception that the previous return to work was harmful, and retrospectively not recommended.	
**Skills for job procurement, externally assessed**	Initiating employment	Self-reported skill level is biased, why it should be externally assessed. Yet, job skills are most important for work participation, since work implies other stakeholders (costumers, employers etc.) should perceive work or services to have been delivered to pay money (which requires skills!)	
			I send you my proposals, but *I am not sure*, whether my

			choices/preferences are "missing outcomes" or just already mentioned outcomes. The reason for my doubts are thate the adjustments I propose might be so slight that the outcomes do not count as other and hence missing outcomes.
**Employment status operationalization**	Having employment	a) Labour market status during the last week of each year<br><br>OR<br><br>b) The main activity (i.e., labour market status) during the year<br><br>OR<br><br>c) The measure of labour market status, based on labour market status during each month.<br><br>###<br><br>a) and b) are strongly correlated.<br><br>We have used almost always a).	
**"Having lost employment" - early retirement**	Having employment	This seems to also be an important outcome	
**Time until employment since finishing school/vocational training**	Initiating employment	Would also be a more objective and internationally comparable outcome.	
