# Supplementary material for: Which outcomes should always be measured in intervention studies for improving work participation for people with a health problem? An international multistakeholder Delphi study to develop a core outcome set for Work participation (COS for Work)
Source: BMJ Open. 2023 Feb 15;13(2):e069174. doi: 10.1136/bmjopen-2022-069174 (PMC9933745; doi:10.1136/bmjopen-2022-069174)
Supplement: Supplementary data [file bmjopen-2022-069174supp005.pdf]

This document contains 35 additional outcomes which were suggested by participants in the first round of Delphi voting. We consider whether the suggested outcomes match the criteria for outcomes suitable for COS for Work.

We would also like to draw your attention to the overarching definition of work participation used for COS for Work: *"work participation entails either performing work related activities or having the capacity to do a work related activity".* This definition is based on the definition of participation provided by the International Classification of Functioning, Disability and Health (ICF). The operationalization of this definition is not straight forward but the outcomes have to measure at the level of (not) performing a work related activity.

In addition, we use the criteria for outcomes suitable for the COS which were developed as part of the COS for Work framework.

For more reading on defining work participation, operationalization of outcomes and of the criteria, the framework can be accessed via **https://doi.org/10.21203/rs.3.rs-1342930/v1**

COS for Work outcomes criteria.

Criteria to consider	***Optional criteria***
Outcomes should:<br>1. Be sensitive to change<br>2. Be feasible to measure<br>3. Be applicable internationally<br>4. Be work participation specific<br>5. Capture multiple stakeholder perspectives<br>6. Be in alignment with the ICF model	*Outcomes should:*<br>1. Be used for cost-effectiveness studies<br>2. Be applicable across varying insurance schemes

Below we provide our argumentation for or against inclusion of the suggested outcomes in the next round of Delphi voting. If you disagree with our decision, there will be room to discuss this during our meeting on May 25.

Suggested outcome	Yes or No from COS team to include in next round	Reason
**Caregiver outcomes i.e. does caregiving affect work production**	No	This is an indirect variable/outcome. A contextual factor which may impact work but does not directly measure at either mental or physical level of "activity" by an individual.
**Worker satisfaction with job**	No	Job satisfaction was already included under outcome concept 2.5.1 "perceptions affecting productivity".
**Worker preceived stigma/bias from employer/society**	No	(Self) perceived stigma was already included under outcome concept 2.5.1 "perceptions affecting productivity".
**Provided with accomodation(s) for equitable work environment**	No	This is an indirect variable/outcome. A contextual factor which may impact work but does not directly measure at either mental or physical level of "activity" by an individual.
**Perceived social support - includes support from employer and access to appropriate work modifications)**	No	This is an indirect variable/outcome. A contextual factor which may impact work but does not directly measure at either mental or physical level of "activity" by an individual.
**Satisfaction of the worker with its career**	No	Job satisfaction was already included under outcome concept 2.5.1 "perceptions affecting productivity".
**Sustainable employment**	Yes	
**Work Ability Score (WAS)**	No	This is a measurement instrument which would measure the outcome 2.3.1 "work ability".
**Employment 3 - 5 years post injury/illness**	No	This would be a more specific definition of the outcome concept 3.1.4 (duration of employment).
**Accommodations requested and received**	No	This is an indirect variable/outcome. A contextual factor which may impact work but does not directly measure at either mental or physical level of "activity" by an individual.
**Quality of life**	No	Does not match criteria 4: COS for Work outcomes should be work participation specific.
**Health Status**	No	Does not match criteria 4: COS for Work outcomes should be work participation specific.

**Employer attention to occupational safety and health**	No	This is an indirect variable/outcome. A contextual factor which may impact work but does not directly measure at either mental or physical level of "activity" by an individual.
**Worker participation in job design**	No	This outcome is relevant in the context of workers participation decision making processes rather than evaluating a person's ability to work despite of a health problem.
**Worker participation in evaluation of injury control interventions**	No	This outcome is relevant in the context of workers participation decision making processes rather than evaluating a person's ability to work despite of a health problem.
**Adequacy of compensation system in replacing lost income after disability**	No	This is an indirect variable/outcome. A contextual factor which may impact work but does not directly measure at either mental or physical level of "activity" by an individual.
**Injury rate after return to work**	No	Does not match criteria 4: COS for Work outcomes should be work participation specific.
**Work Participation Score within 12 months following RTW; defined as a fraction with numbers of RTW weeks in the numerator and numbers of weeks receiving health related benefits in the denominator. K. Biering; N.H. Hjollund and T. Lund 2013**	No	This is at the level of measurement of RTW rather than on the level of outcome concepts.
**Average time from requested evaluation to determination**	No	Does not match criteria 4: COS for Work outcomes should be work participation specific.
**Possibilities to get a suitable job in the same organisation/group**	No	This would fall under the outcome concept 3.1.2 (having a pre-defined status of employment).
**Having support by your colleques - possibillitie that colleques take over task by your absence**	No	This is an indirect variable/outcome. A contextual factor which may impact work but does not directly measure at either mental or physical level of "activity" by an individual.
**Safe environment/culture in the organsiation by meaning workers**	No	This is an indirect variable/outcome. A contextual factor which may impact work but does not directly measure at either mental or physical level of "activity" by an individual.
**Number of HR coach /buddy's for returning/adjust to job in % total employees of organisations**	No	This is an indirect variable/outcome. A contextual factor which may impact work but does not directly measure at either mental or physical level of "activity" by an individual.
**Flexibility in hours and location to fulfill your tasks**	No	This is an indirect variable/outcome. A contextual factor which may impact

		work but does not directly measure at either mental or physical level of “activity” by an individual.
**Amount that is invested in hardware to maintain /keep workers (for instead invalid toilets rsi mouse)**	No	This is an indirect variable/outcome. A contextual factor which may impact work but does not directly measure at either mental or physical level of “activity” by an individual.
**Ability to visit a work related docter (arbocurative)**	No	This is an indirect variable/outcome. A contextual factor which may impact work but does not directly measure at either mental or physical level of “activity” by an individual.
**Work satisfaction vs normal workers**	No	Job satisfaction was already included under outcome concept 2.5.1 “perceptions affecting productivity”.
**Level of education**	No	Baseline characteristic rather than an outcome measured at the start and end of an intervention to establish effectiveness.
**Sort of work (physical or mental capacity needed for the job)**	No	This is an indirect variable/outcome. A contextual factor which may impact work but does not directly measure at either mental or physical level of “activity” by an individual.
**Is the sick leave from a work related injury**	Yes/No	To be discussed
**Is there paid sick leave at the workplace**	No	This is an indirect variable/outcome. A contractual factor.
**Did workers see a company provided health care person or receive outside medical care**	No	This is an indirect variable/outcome. A contextual factor which may impact work but does not directly measure at either mental or physical level of “activity” by an individual.
**Did the person get any workers compensation for their injury**	No	This is an indirect variable/outcome. A contractual factor.
**If a work related injury; was the workplace changed to reduce chance of reinjury?**	No	This is an indirect variable/outcome. A contextual factor which may impact work but does not directly measure at either mental or physical level of “activity” by an individual.
**Hybrid work if position allows - in person and at home**	No	This is an indirect variable/outcome. A contextual factor which may impact work but does not directly measure at either mental or physical level of “activity” by an individual.
