# Supplementary material for: Which outcomes should always be measured in intervention studies for improving work participation for people with a health problem? An international multistakeholder Delphi study to develop a core outcome set for Work participation (COS for Work)
Source: BMJ Open. 2023 Feb 15;13(2):e069174. doi: 10.1136/bmjopen-2022-069174 (PMC9933745; doi:10.1136/bmjopen-2022-069174)
Supplement: Supplementary data [file bmjopen-2022-069174supp006.pdf]

Results of the first round ratings combined for all stakeholders and stakeholder groups separately. Ratings are presented for each stage from most to least highly rated; a combination of mean score ($\overline{x}$) and the percentage of the stakeholders which rated an outcome with a score between 7-9. N.a.: not applicable. Stages of work participation: (1) initiating employment, (2) having employment, (3) increasing or maintaining productivity at work and (4) return to employment.

Stage	Outcome; clarifying text	Overall (n=55) $\overline{x}$; % 7-9	Researcher (n=35) $\overline{x}$; % 7-9	Employee (n=10) $\overline{x}$; % 7-9	Policy Maker (n=5) $\overline{x}$; % 7-9	Occupational Health Professional (n=4) $\overline{x}$; % 7-9	Employer (n=1) score
1	**Time to employment;** Time till getting a job	5,6; 44%	5,9; 51%	5,7; 50%	4,6; 0%	5; 25%	1 n.a.
1	**Self-efficacy for job procurement;** Self-efficacy for job procurement; Hope, optimism and self-efficacy for achieving vocational success; Social/intepersonal self-efficacy	5,6; 38%	5,4; 31%	6,1; 50%	6,2; 60%	5,8; 50%	1 n.a.
1	**Skills for job procurement;** Total work-related network, Engagement in work seeking activities, Job interview skills, Work-related benefit of vocational training	5,4; 38%	5; 29%	6,4; 60%	5,2; 40%	5,3; 50%	9 n.a.
2	**Employment - any type of employment including self-employment;** Any type of employment including self-employment	7,4; 75%	7,6; 80%	7,2; 70%	6,6; 60%	7; 75%	9 n.a.
2	**Employment - having lost**	6,5; 55%	6,4; 54%	7,5; 80%	5,4; 20%	7,3; 50%	1 n.a.

	**employment;** Having lost employment						
2	**Employment - predefined type of employment;** Having met a pre-defined status of employment; part/full time, competitive work in a mainstream setting, performing the same tasks as non-disabled workers	6,5; 51%	6,5; 51%	7,1; 60%	7,4; 60%	5; 25%	1 n.a.
2	**Work disability - Permanent and complete inability to engage in any work participation;** Permanent and complete inability to engage in any work participation	6,7; 34%	6,9; 63%	7,2; 80%	6; 40%	6,3; 50%	1 n.a.
2	**Work disability - Permanent partial disability to engage in work participation;** Permanent partial disability to engage in work participation	6,4; 31%	6,6; 51%	7,5; 90%	6; 40%	6; 50%	1 n.a.
2	**Employment - duration of employment;** Duration of employment	5,7; 40%	5,9; 40%	6; 50%	5,4; 20%	5; 50%	1 n.a.
2	**Employment - predefined type or amount of income;** Predefined type or amount of income	5,5; 33%	5,1; 23%	6,9; 70%	5,4; 20%	4.3; 25%	9 n.a.

3	**Work productivity loss (economic evaluation);** Overall loss of productivity at work (in terms of quality or output at work) resulting from presenteeism or absenteeism regardless of cause. Typically used for economic evaluations.	6,4; 58%	6,7; 63%	6,9; 70%	6; 40%	5; 25%	1 n.a.
3	**Work activity impairment;** Experienced functional impairment of performing work activities due to a health problem.	6,4; 51%	5,9; 40%	7,7; 80%	6; 40%	7,5; 75%	9 n.a.
3	**Work ability;** Current self-rated physical and mental work ability compared to lifetime best.	6,3; 53%	6,3; 46%	7,1; 90%	6,2; 40%	5,6; 50%	1 n.a.
3	**Presenteeism;** Not being productive while at work due to health problems. Typically used to evaluate the effect of health interventions for individuals.	5,9; 35%	5,8; 29%	6,7; 70%	4,6 ; 0%	5,3; 25%	9 n.a.
3	**Perceptions affecting productivity;** Beliefs in working capacity, motivation for work, vocational commitment, job coping, job satisfaction, need	5,5; 36%	5,1; 26%	7,2; 80%	5,2; 40%	4,3; 25%	9 n.a.

	for recovery, work-life balance, (self)-perceived stigma						
4	**Return to work - proportion of workers;** Proportion of workers that return to work after being absent because of illness	7,4; 76%	7,7; 80%	7; 70%	7,8; 80%	7,5; 75%	1 n.a.
4	**Return to work - time to return to work;** Time to return to work or duration of sickleave after being absent because of illness	7; 67%	7,3; 71%	6,8; 60%	6,8; 60%	7; 75%	1 n.a.
4	**Sustainable return to work – proportion of workers;** Proportion of workers that return to work and remain at work for a specified amount of time	6,7; 57%	6,3; 49%	7,6; 80%	7,2; 60%	6; 50%	9 n.a.
4	**Sustainable return to work – time until;** Time to return to work in workers absent because of illness and who do not relapse within a specified amount of time	6,3; 49%	6,1; 42%	6,9; 60%	6,6; 60%	5,8; 50%	9 n.a.
4	**Sick leave frequency;** Number of episodes of sick leave *per worker* expressed as percentage	5,8; 44%	5,4; 37%	6,9; 60%	5,6; 40%	5,5; 50%	9 n.a.
4	**Return to work - the worker's perceptions;** Self-efficacy for return	5,7; 38%	5; 20%	7,8; 80%	4,3; 20%	4,3; 25%	9 n.a.

	to work, return to work expectations or intention to return to work						
4	**Sick leave duration of all workers;** Working days or calendar days absent because of illness averaged over all workers	5,5; 38%	5,4; 37%	6,8; 50%	5; 20%	4; 25%	1 n.a.
4	**Sick leave duration - over all sick leave episodes;** Working days absent averaged over all sick leave episodes; can also be expressed as calendar days sick	5,4; 36%	5,6; 34%	6,8; 60%	4,8; 20%	3; 25%	1 n.a.
4	**Sickness absence among absent workers in an organization/group - lost working or sick days;** Working days or lost sick days as a percentage	5,3; 33%	5,3; 31%	6,1; 40%	6; 40%	4,3; 25%	1 n.a.
4	**Sickness absence among all workers;** Percentage of all working days of all workers in an organization during a time period e.g. 12 months; Sick days as percentage of all available days during a time period	5,3; 31%	5,3; 29%	6,8; 50%	6; 20%	4,3; 25%	1 n.a.
