# Supplementary material for: Which outcomes should always be measured in intervention studies for improving work participation for people with a health problem? An international multistakeholder Delphi study to develop a core outcome set for Work participation (COS for Work)
Source: BMJ Open. 2023 Feb 15;13(2):e069174. doi: 10.1136/bmjopen-2022-069174 (PMC9933745; doi:10.1136/bmjopen-2022-069174)
Supplement: Author's manuscript [file bmjopen-2022-069174.draft_revisions.pdf]

BMJ Open

BMJ Open is committed to open peer review. As part of this commitment we make the peer review history of every article we publish publicly available.

When an article is published we post the peer reviewers' comments and the authors' responses online. We also post the versions of the paper that were used during peer review. These are the versions that the peer review comments apply to.

The versions of the paper that follow are the versions that were submitted during the peer review process. They are not the versions of record or the final published versions. They should not be cited or distributed as the published version of this manuscript.

BMJ Open is an open access journal and the full, final, typeset and author-corrected version of record of the manuscript is available on our site with no access controls, subscription charges or pay-per-view fees (http://bmjopen.bmj.com).

If you have any questions on BMJ Open's open peer review process please email info.bmjopen@bmj.com

BMJ Open

A Core Outcome Set for work participation: an international multi-stakeholder Delphi study

Journal:	*BMJ Open*
Manuscript ID	bmjopen-2022-069174
Article Type:	Original research
Date Submitted by the Author:	14-Oct-2022
Complete List of Authors:	Ravinskaya, Margarita; Amsterdam UMC Locatie AMC, Department of Public and Occupational Health, Coronel Institute of Occupational Health, University of Amsterdam, Amsterdam Public Health research institute, Cochrane Work,<br>Verbeek, Jos ; University of Amsterdam, Amterdam UMC, Department of Public and Occupational Health, Coronel Institute of Occupational Health, University of Amsterdam, Amsterdam Public Health research institute, Cochrane Work<br>Langendam, Miranda; Amsterdam UMC Locatie AMC, Department Epidemiology and Data Science, University of Amsterdam, Amsterdam Public Health research institute<br>MADAN, IRA; GUYS AND ST THOMAS NHS FOUNDATION TRUST; King's College London Faculty of Life Sciences and Medicine<br>Verstappen, Suzanne; University of Manchester, Centre for Epidemiology Versus Arthritis, Faculty of Biology, Medicine and Health; NIHR Manchester Biomedical Research Centre<br>Kunz, Regina; University Hospital Basel, Research Unit EbIM, Evidence Based Insurance Medicine, Division of Clinical Epidemiology<br>Hulshof, C.; University of Amsterdam, Amsterdam UMC, Academic Medical Center, Department of Public and Occupational Health, Coronel Institute of Occupational Health, University of Amsterdam, Amsterdam Public Health research institute<br>Hoving, Jan; Amsterdam UMC Locatie AMC, Department of Public and Occupational Health, Coronel Institute of Occupational Health, University of Amsterdam, Amsterdam Public Health research institute, Cochrane Work
Keywords:	OCCUPATIONAL & INDUSTRIAL MEDICINE, PUBLIC HEALTH, REHABILITATION MEDICINE

SCHOLARONE™
Manuscripts

I, the Submitting Author has the right to grant and does grant on behalf of all authors of the Work (as defined in the below author licence), an exclusive licence and/or a non-exclusive licence for contributions from authors who are: i) UK Crown employees; ii) where BMJ has agreed a CC-BY licence shall apply, and/or iii) in accordance with the terms applicable for US Federal Government officers or employees acting as part of their official duties; on a worldwide, perpetual, irrevocable, royalty-free basis to BMJ Publishing Group Ltd ("BMJ") its licensees and where the relevant Journal is co-owned by BMJ to the co-owners of the Journal, to publish the Work in this journal and any other BMJ products and to exploit all rights, as set out in our licence.

The Submitting Author accepts and understands that any supply made under these terms is made by BMJ to the Submitting Author unless you are acting as an employee on behalf of your employer or a postgraduate student of an affiliated institution which is paying any applicable article publishing charge ("APC") for Open Access articles. Where the Submitting Author wishes to make the Work available on an Open Access basis (and intends to pay the relevant APC), the terms of reuse of such Open Access shall be governed by a Creative Commons licence – details of these licences and which Creative Commons licence will apply to this Work are set out in our licence referred to above.

Other than as permitted in any relevant BMJ Author's Self Archiving Policies, I confirm this Work has not been accepted for publication elsewhere, is not being considered for publication elsewhere and does not duplicate material already published. I confirm all authors consent to publication of this Work and authorise the granting of this licence.

For peer review only

A Core Outcome Set for work participation was developed in an international multi-stakeholder Delphi study

Margarita Ravinskaya [a], Jos H. Verbeek [a], Miranda Langendam [c], Ira Madan[d,h], Suzanne M.M. Verstappen [f, g, h], Regina Kunz [e], Carel T.J. Hulshof [b], Jan L. Hoving [a]

[a] Cochrane Work, Amsterdam UMC, Academic Medical Center, University of Amsterdam, Coronel Institute of Occupational Health, Amsterdam Public Health research institute, Meibergdreef 9, 1105 AZ, Amsterdam, The Netherlands

[b] Amsterdam UMC, location Academic Medical Center, University of Amsterdam, Coronel Institute of Occupational Health, Amsterdam Public Health research institute, Meibergdreef 9, 1105 AZ, Amsterdam, The Netherlands

[c] Amsterdam UMC location Academic Medical Centre, Epidemiology and Data Science, Amsterdam, Amsterdam Public Health, Methodology, Meibergdreef 9, 1105 AZ, Amsterdam, The Netherlands

[d] Guy's and St Thomas' NHS Trust and Faculty of Life Sciences and Medicine, King's College London, United Kingdom

[e] Research Unit EbIM, Evidence-Based Insurance Medicine, University of Basel, Petersgraben 4, 4031 Basel, Switzerland

[f] Centre for Epidemiology Versus Arthritis, Faculty of Biology, Medicine and Health, The University of Manchester, Oxford Road, Manchester M13 9PL, United Kingdom

[g] NIHR Manchester Biomedical Research Centre, Manchester University NHS Foundation Trust, Manchester Academic Health Science Centre, 29 Grafton St, Manchester M13 9WU, United Kingdom

[h] MRC Versus Arthritis Centre for Musculoskeletal Health and Work, University of Southampton, University Rd, Highfield, Southampton SO17 1BJ, United Kingdom

Corresponding author. Margarita Ravinskaya. Cochrane Work, Amsterdam UMC, University of Amsterdam, Department of Public and Occupational Health, Coronel Institute of Occupational Health, Amsterdam Public Health research institute, Meibergdreef 9, 1105 AZ Amsterdam, The Netherlands. Tel.: +31 20 566 5337 E-mail address: m.ravinskaya@amsterdamumc.nl

Word count: 3354

For peer review only

ABSTRACT

Objective Synthesizing evidence of the effects of interventions to improve work participation among people with health problems is currently difficult due to heterogeneity in outcome measurements. A core outcome set for work participation is needed.

Study design and setting Following the Core Outcome Measures in Effectiveness Trials (COMET) methodology we used a five-step approach to reach international multi-stakeholder consensus on a core outcome set for work participation. Five sub-groups of stakeholders took part in two rounds of discussions and completed two Delphi voting rounds on 26 outcomes. A consensus of >= 80% determined core-outcomes and 50%-80% consensus was required for candidate-outcomes.

Results Fifty-eight stakeholders took part in the Delphi rounds. Core outcomes were: “any type of employment including self-employment”, “proportion of workers that return to work after being absent because of illness” and “time to return to work”. Ten candidate outcomes were proposed, among others: “sustainable employment”, “work productivity”, and “workers’ perception of return to work”.

Conclusion As a minimum, all studies evaluating the impact of interventions on work participation should include one employment outcome and two return to work outcomes if workers are on sick leave prior to the intervention.

Keywords work participation, employment, return to work, outcome measurements, core outcome set

Strengths and limitations of this study

- This is the first core outcome set for work participation (COS for Work) applicable for any type of health problem and any intervention which includes measures of work participation either as a primary or secondary outcome
- The study was completed in accordance with the Core Outcome Measures in Effectiveness Trials (COMET) guidelines
- Five key stakeholders were involved in the consensus process: researchers, occupational health professionals, policy makers, employer and employee/patient representatives
- The majority of the stakeholders were researchers
- The majority of the stakeholders were from Europe and North America

For peer review only

INTRODUCTION

Authors of systematic reviews (SRs) state that inconsistent reporting of work participation outcomes in clinical trials hampers evidence synthesis in the field of occupational health (OH).(1-3) Co-ordinating Editors of Cochrane review groups have indicated that the reliability and quality of SRs could be improved by using core outcome sets.(4) In 2019, the Coronel Institute of OH at AMC Amsterdam established an international research collaboration to develop a Core Outcome Set for Work Participation (COS for Work), based on the Core Outcome Measures in Effectiveness Trials (COMET) methodology.(5) Our aim was to develop a COS for Work to be used by researchers in intervention studies in which work participation is being measured as a primary or secondary outcome.

The first phase of our project involved a SR on the spectrum of work outcomes used to measure the effect of interventions in published randomized controlled trials (RCTs).(6) The SR showed extensive heterogeneity in work-participation outcome measurements and confirmed the need for a COS for Work. In addition, we saw that it was unclear why authors chose to measure outcomes in a certain way and that to create COS for Work we would need to have a framework for outcomes which would ensure meaningful, pragmatic and mutually exclusive categories in which outcomes could be placed.

In the second phase, we created a framework to aid the selection of work participation outcomes (submitted for publication). The framework outlines four main stages of work participation: outcomes assessing whether a person is successful in acquiring or *initiating employment (stage 1)*, outcomes indicating whether a person is in employment, can retain employment or lost work within the duration of the study, i.e., *having employment (stage 2)*, outcomes that measure *increasing or maintaining productivity at work (stage 3)* when persons experience limitations or restrictions with working or have less output, and outcomes that assess *return to employment and sick leave from work (stage 4)* when people (temporarily) stop attending work and are on sick leave. Phase three involved a survey among reviewers and trial authors showing different reasons and preferences for choosing and using work outcomes. In particular, researchers choose outcomes because of their use in similar studies or their relevance for the health problem or type of intervention.(7)
In the fourth phase, we sought international consensus on COS for Work via a Delphi study. In the fifth and final phase, we will make recommendations for clinimetrically sound methods to measure the core outcomes selected during phase four.
The overall aim of the Delphi study is to define a comprehensive and minimal set of outcomes that is relevant and feasible for measuring the effectiveness of interventions that result in a change in work participation, based on consensus of an international, multi-stakeholder group.

METHODS

On-line multi-stakeholder discussions and Delphi voting

To develop methods we followed the COMET guidelines,(5) the Core Outcome Set—STAndards for Development: The COS-STAD (8) and the Core outcome set–STAndards for reporting: the COS-STAR (9) to develop the methods. The protocol for this study was published on the COMET website prior to the commencement of the study (https://www.comet-initiative.org/studies/details/1195, online supplementary file 1), and includes an extensive explanation of the methodology. The five steps are summarized briefly below.

Step 1: preparation

We invited five groups of stakeholder representatives: OH researchers, OH professionals, workers and patients, employers and policy makers. Stakeholders were recruited from our phase two survey (6) and our professional network. We anticipated that an effective group discussion with up to 30 participants would be feasible to allow diversity of opinion in the various subgroups. Our aim was to organize two group discussions of 30 participants, in the morning and late afternoon to account for the varying time zones. We sent out 90 invitations.

Stakeholders who agreed to participate received an information package that included [1] instructions for Delphi participation (online supplementary file 2), [2] a document containing the outcomes that would be

For peer review only - http://bmjopen.bmj.com/site/about/guidelines.xhtml

included in the Delphi with worksheets to prepare for the first discussion (table 1, online supplementary file 3) and [3] a list of all participants in each discussion group. Item generation for the twenty four outcomes in the preliminary set (table 1) were based on the SR (6) and categorized under the four work participation stages as described in the framework (table 1).

Participants were invited to familiarize themselves with the material and add any outcomes, which they deemed missing from the preliminary list prior to the first discussion (online supplementary file 4). The research team evaluated whether these new outcomes fitted the COS for Work criteria (table 2) and presented the outcomes during the first discussion round.

For peer review only

Table 1. The preliminary set of outcomes that was presented to the stakeholders prior to the first discussion. *Item generation was based on the systematic review on how work participation outcomes are currently measured. Online supplementary file 3 contains the full overview with examples of how the outcomes could be measured.*

Stage of work participation — interventions and study populations for which outcomes are relevant within the stage	Outcome to be voted on during the Delphi
**Stage 1: Initiating employment:**<br><br>Interventions aiming to help unemployed people with distance to the job market due to a health problem get work	**1. Skills for job procurement.** Total work-related network, Engagement in work seeking activities, Job interview skills, Work-related benefit of vocational training
	**2. Self-efficacy**. Self-efficacy for job procurement, hope, optimism, and self-efficacy for achieving vocational success, social/interpersonal self-efficacy
**Stage 2: Having employment**<br><br>Relevant for any intervention and any type of health problem. Any type of health condition may impair work participation at the most elemental level, having work	**3. Employment. Having any type of employment including self-employment**
	**4. Employment. Having met a pre-defined status of employment**; part/full time, competitive work in a mainstream setting, performing the same tasks as non-disabled workers
	**5. Employment. Predefined type or amount of income**
	**6. Employment. Duration of employment**
	**7. Employment. Having lost employment**
	**8. Work disability.** Permanent and complete inability to engage in any work participation
	**9. Work disability.** Permanent partial disability to engage in work participation
**Stage 3: Increasing or maintaining productivity at work**<br><br>Interventions expected to help workers who are not on sick leave to maintain or increase productivity at work despite a health problem	**10. Work productivity loss (economic evaluation).** Overall loss of productivity at work (in terms of quality or output at work) resulting from presenteeism or absenteeism regardless of cause. Typically used for economic evaluations
	**11. Presenteeism.** Not being productive while at work due to health problems. Typically used to evaluate the effect of health interventions for individuals
	**12. Work ability.** Current self-rated physical and mental work ability compared to lifetime best.
	**13. Work activity impairment.** Experienced functional impairment of performing work activities due to a health problem
	**14. Perceptions affecting productivity.** Possible constructs: beliefs in working capacity, motivation for work, vocational commitment, job coping, contentment with work, need for recovery
**Stage 4: Return to employment and sick leave from work**<br><br>***Return to work:***<br>Interventions expected to help people who are on sick leave return to work	**15. Return to work - proportion of workers that return to work** after being absent because of illness
	**16. Return to work - time to return to work** or duration of sick leave after being absent because of illness
	**17. Sustainable return to work.** Proportion of workers that return to work and remain at work for a specified amount of time
	**18. Sustainable return to work.** Time to return to work in workers absent because of illness and who do not relapse within a specified amount of time
	**19. Return to work - the worker's perceptions.** Time it will take to return to work as perceived by the sick worker

For peer review only

Sick leave from work: (1) Interventions including people at risk of (frequent) sick leave due to a health problem, e.g. chronic conditions or recovery after an injury or procedure (2) Interventions aiming to prevent overall sickness absence among all workers	also described as self-efficacy for return to work, return to work expectations or intention to return to work
	**20. Sickness absence among all workers** in an organization/ group expressed as lost working days or lost sick days
	**21. Sick leave duration.** Working days absent because of illness averaged over all workers
	**22. Sick leave frequency**. Number of episodes of sick leave *per worker* expressed as percentage
	**23. Sickness absence among absent workers** in an organization/group expressed as lost working days or lost sick days
	**24. Sick leave duration.** Working days absent because of illness averaged over all workers

Table 2: Criteria for COS outcomes which the stakeholders were asked to consider when deciding for or against inclusion of outcomes in COS for Work.

Criteria to consider	*Optional criteria*
Outcomes should: 1. **Be sensitive to change** 2. **Be feasible to measure** 3. **Be applicable internationally** 4. **Be work participation specific** 5. **Capture multiple stakeholder perspectives** 6. **Be in alignment with the ICF model**	*Outcomes should if possible/applicable:* 1. Be used for cost-effectiveness studies 2. Be applicable across varying insurance schemes

Step 2: First online consensus stakeholder meeting

In the first online meeting, we presented the steps and research findings of the COS development including the four stages of work participation (see Table 1) and criteria for COS outcomes (table 2). Further, we elaborated on the various stakeholder perspectives and its effect on outcome prioritization, and discussed the proposed additional outcomes. In breakout rooms, groups of stakeholders created and discussed “absolutely in” and “absolutely out” outcomes for each stage. The results were discussed in the plenary session.

Step 3: First round of Delphi voting

COMET online Delphi Manager software participants voted on a total of twenty-five outcomes (outcomes from table 1 plus one outcome suggested by a stakeholder) accompanied by clarifying text on how it could be measured. With a personal login, the DelphiManager software allowed each outcome to be anonymously ranked on a Likert Scale from 1-9: from not important (0 points) to critical (9 points), including an “unable to score” option. Participants were asked to provide rationale for their ratings and additional outcomes could be suggested. A single voting reminder was sent to participants two days before the closing of the first round.

Step 4: Second online stakeholder meeting

The results of the first online Delphi voting round and a collated list of arguments on why to include or exclude an outcomes were first distributed via e-mail and then discussed in the second online stakeholder meeting. We also presented our analysis of suggested additional outcomes (online supplementary file 5). Based on the a-priori defined consensus definition (see section 2.2) and input from the stakeholder meeting, the authors then decided which outcomes would be dropped, included, reformulated or added to the second Delphi round.

Step 5: Second Delphi voting round

For peer review only

The same online Delphi procedure allowed each participant to individually rate the outcomes that remained from the first Delphi round. As before, reminders were sent to non-completers.

Patient and Public involvement

Consultation of patients' is seen as a crucial aspect in the development of COS (5). We have consulted patient representatives, who were also representing the 'employee' perspective, in the discussion rounds and the selection of core outcomes for COS for Work. The patient representatives were members of, amongst others: The Netherlands Patients Federation, Canadian Injured Workers Alliance and labor unions. In addition, we also involved policy makers active in the field of occupational health. The results were disseminated to all participants via e-mail.

Data analysis

After the first and second stakeholder meetings we held a meeting with the steering group to discuss how we could use and present the stakeholder input for the Delphi voting to inform all Delphi participants. The results from the Delphi rounds were extracted from the Delphi Manager software into a CSV format and analyzed in SPSS using descriptive statistics.

For each round, we calculated the distribution of scores for all outcomes as well as the percentage of stakeholder scoring between the 7-9. As per protocol, outcomes scored between 7-9 by at least 80% of all stakeholders were deemed as core – showing as "*definite* core outcome IN" on the spreadsheet distributed amongst stakeholders. Outcomes receiving a mean score between 7 -9, from 50% - 80% of the participants in the first Delphi round were "candidate outcomes" to be discussed with the stakeholders and re-rated in the second Delphi round – labeled as "*possible* core outcome IN". Outcomes which received a score between 7-9 from less than 50% of the participants were dropped after the first round. For outcomes that were not considered definite core outcome but for which there was strong disagreement between stakeholder groups in the first round, the steering group still considered them for being discussed and rated in the second round. It was possible to upgrade or downgrade the ranking of an outcome for inclusion or exclusion in the second Delphi round based on argumentation of stakeholders. The final core outcome set is based on the rankings of the second round.

RESULTS

Respondents

Initially we approached 90 potential stakeholders and 59 stakeholders agreed to participate (not everyone from this group registered for the Delphi). It was difficult to recruit employer and employee representatives; we had no replies from employee representatives and one employer. After the first discussion round, participants helped us to recruit 11 employee representatives through their professional network. In total 70 stakeholders agreed to participate. Twelve participants took part in either one or two of discussions but did not register for the Delphi Manager software, some of whom e-mailed that they did not manage to due to time constraints (See Table 3 for participant characteristics).

Table 3. Characteristics of survey respondents for two Delphi rounds (n; %). In total 58 participants registered as participants in the Delphi Manager. Each participant belonged to one stakeholder group. Three participants in each round did not start or complete a round.

	Round 1	Round 2
**Stakeholder group**		
All Delphi participants	58; 100%	58; 100 %
• **Researcher**	35; 60%	37; 63%
• **Employee**	10; 17%	10; 17%
• **Occupational Health professional**	4; 7%	4; 7%

• **Employer**	1; 2%	1; 2%
• **Policy maker**	5; 9%	3; 5%
• **Non-completers**	3; 5%	3; 5%;
**Location of residence**		
• **Europe**	31; 53%	30; 52%
• **North America**	17; 31%	17; 29%
• **Australia**	3; 5%	4; 7%
• **Asia**	3; 5%	3; 5%
• **South America**	1; 2%	1; 2%

First online consensus stakeholder discussion

We presented all Delphi voting information (ratings and arguments) to the stakeholders as outlined in section 2.1. The stakeholders discussed their preferences for and against the outcomes in four breakout rooms. They then presented their arguments in a plenary session that included discussion on topics such as:

- when work can be considered as sustainable and healthy and the challenges of measuring such concepts as part of this COS,
- which types of outcomes would be applicable and feasible to measure in an international context,
- why COS outcomes should have a positive focus (such as work-ability) rather than negative (such as sick leave).

We received fifteen outcomes (online supplementary file 4) which stakeholders found missing from the preliminary set (table 1). We did not include the outcomes which did not match with the COS criteria (table 2), outcomes which were already included but phrased slightly different, and outcomes which were a specification on the level of measurement. Two outcomes were added to the list for the first round of Delphi voting: (1) "(self)-perceived stigma" to the *perceptions affecting productivity* outcome concept and (2) "time to employment" to the *initiating employment* stage.

First round of Delphi voting and second online stakeholder discussion

The ratings for the first round of Delphi voting are presented in the online supplementary file 6. None of the outcomes within the four stages received our cut-point for "definite core outcome IN" (>80% score of 7-9 points).

<u>Stage 1: Initiating employment</u>

The highest-ranking outcome was "time to employment" with a mean score of 5.6 and 44% of stakeholders giving it a score between 7 - 9. To not entirely exclude this stage from the second round, the steering group included the newly proposed outcome "any type of employment including self-employment" for the second Delphi round.

<u>Stage 2: Having employment</u>

"Any type of employment" was the highest-ranking outcome with a mean score of 7.4 and 75% of the stakeholders scoring it between 7-9. Stakeholders indicated that this outcome was feasible to measure internationally irrespective of social insurance schemes. In addition, this outcome was also seen as an indicator of the degree of work participation when sickness absence is not measured, and people lose work because of a health problem - relevant for countries where sickness benefits are limited. An additional outcome was proposed by a stakeholder, discussed, and agreed for inclusion in the second round: "sustainable employment" (online supplementary file 5). The employee representatives advocated to define "sustainable" in terms of quality of the working life, such as having decent work and healthy work.

<u>Stage 3: Increasing or maintaining productivity at work</u>

“Work productivity loss”, “Work activity impairment” and “Work ability” received “possible core outcome IN” rankings, and during the discussion, there were no objections, biddings to downgrade any of them or exclude them from the second round. “Perceptions affecting productivity” was ranked to be left out from the second round. However, we did not exclude this outcome as the outcome concept included measures such as work-life balance and job-satisfaction, which were brought forward as highly important by several stakeholders during the discussion. Therefore, it was upgraded for inclusion during the discussion.

Stage 4: Return to employment

The steering group discussed the disagreement on “Return to work – workers’ perceptions’ which were voted out by all stakeholder groups combined. The main argument against including it was whether the outcome actually measured work participation and whether it would be relevant to measure at the beginning and end of all types of interventions. However, this outcome was ranked as “definite core outcome IN” by the employee representatives and some stakeholders from other groups were keen on including it in the second round. Therefore, it was not excluded All versions of sickness absence outcomes received low ratings. Feedback from stakeholders suggested that this may be due to varying insurance schemes, how it is registered, lack of compensation of sick leave in many countries and in some cases punitive measures for being on registered sick leave. However, as there are interventions for which it is relevant to measure accumulated absence over time due to a health problem, and return to work may not be a relevant measure, the steering group decided to keep one absence outcome in the second round. We created a new outcome for the second round, with the aim of replacing sick leave terminology: “Duration of absence from work due to a health problem over a period of time”. In addition, we added one outcome “having lost employment” to this stage, as an indicator of workers who do not return to work at all after a period of health-related absence.

3.4 *Second Delphi round – final vote on core outcomes*

Results of the second round are presented in table 4 and table 5 contains the COS for Work.

Stage 1: Initiating employment

“Having any type of employment including self-employment” as an outcome for interventions aiming to help unemployed people get work did not meet the consensus criteria to be included in the COS for Work.

Stage 2: Having employment

“Any type of employment including self-employment” received 87% consensus and was therefore included in the COS for Work

Stage 3: Increasing or maintaining productivity at work

None of the outcomes in this stage received enough consensus to be included in the COS for Work. However, “work productivity loss”, “work ability” and “work activity impairment” received similar ratings to round one indicating they are seen as important but not critical for the COS for Work.

Stage 4: Return to employment

Two outcomes received overall consensus for inclusion: (1) “Return to work – proportion” and (2) “Time to return to work”. “Proportion of workers with sustainable return to work” was voted as important but not critical by all stakeholders. “Workers’ perception on return to work” received 90% agreement within the employee representatives group for inclusion but very low scores by other stakeholder groups. The “duration of absence from work due to health problem over a period of time” also received low scores for inclusion (38% score between 7-9), making it a higher rating than all the sick leave outcomes from the first round – but nonetheless excluding any absenteeism outcome from consideration for the COS for Work.

Table 4. Results of the second-round ratings combined for all stakeholders combined and stakeholder groups separately. Ratings are presented for each stage from most to least highly rated; including the mean

For peer review only

score ($\bar{x}$) and the percentage of the stakeholders which rated an outcome with a score between 7-9. N.a.: not applicable.

**Outcome**<br><br>**Clarifying text for new outcomes added to the second round (table 1 contains all initial outcomes)**	**Overall (n=55)**<br>$\bar{x}$; % 7-9	**Researcher (n=37)**<br>$\bar{x}$; % 7-9	**Employee (n=10)**<br>$\bar{x}$; % 7-9	**Occupational Health Professional (n=4)**<br>$\bar{x}$; % 7-9	**Policy Maker (n=3)**<br>$\bar{x}$; % 7-9	**Employer (n=1)**<br>**score**
**Stage 1: Initiating employment**						
Any type of employment including self-employment<br>(Outcome replaces all previous outcomes for stage 1. Also included in stage 2.)	6,2; 55%	6,6; 65%	6; 40%	5,3; 25%	6; 33%	1<br>n.a.
Stage 2: Having employment						
Employment - any type of employment including self-employment	7,9; 87%	8; 89%	7,4; 80%	7; 7,5%	8,7; 100%	9<br>n.a.
Sustainable employment<br>(New outcome based on suggestion from Delphi round 1 and the discussion: Any type of employment including self-employment AND one or more indicators of quality of working life (such as work life balance, healthy work, optimal work, work accommodations, decent work)).	7,2; 66%	6,7; 60%	8,6; 100%	7,8; 75%	5; 0%	9<br>n.a.
Work disability - self reported inability to engage in any work participation (Rephrased outcome based on the discussion).	6,9; 64%	7; 65%	7,4; 80%	7,8; 75%	4,3; 0%	1<br>n.a.
Employment - having lost employment	6,5; 56%	6,7; 60%	7,6; 80%	6; 25%	4,3; 0%	1<br>n.a.
**Stage 3: Increasing or maintaining employment**						
Work productivity loss (economic evaluation)	6,4; 62%	6,6; 70%	6,5; 50%	5; 25%	6,7; 67%	1<br>n.a.
Work ability	6,5; 60%	6,6; 60%	7,3; 90%	5,5; 25%	6,7; 67%	1<br>n.a.
Work activity impairment	6; 51%	5,5; 41%	7,5; 80%	6,8; 50%	6; 67%	9<br>n.a.
Perceptions affecting productivity	5,2; 35%	4,7; 24%	6,8; 70%	5; 50%	4,3; 0%	9<br>n.a.
**Stage 4: return to employment**						
Return to employment						
Return to work - proportion of workers	8; 91%	8; 92%	7,6; 90%	8,5; 100%	7,7; 67%	9<br>n.a.
Time to return to work	7,8; 80%	8; 84%	7,6; 80%	7,8; 75%	6; 33%	9<br>n.a.

Sustainable return to work – proportion of workers	7,2; 62%	7,2; 62%	7,7; 70%	7,8; 75%	5,3; 33%	9 n.a.
Duration of absence from work due to health problem over a period of time. (**This outcomes replaces previous sickness absence outcomes based on the suggestions from round 1 and the discussion**)	6; 38%	6; 40%	6,7; 40%	5,5; 25%	6,7; 33%	1 n.a.
Return to work - the worker's perceptions.	5,4; 38%	5,1; 24%	7,4; 90%	3,5; 25%	4,3; 33%	9 n.a.

Table 5: COS for Work. Outcomes which were selected by the panel to always be measured in studies measuring an effect on work participation.

Stage of work participation<br>Interventions and study populations for which the outcomes are relevant within the stage	COS for Work<br>Outcomes which should always be measured in intervention studies relevant for the stage of work participation
**Having employment**<br><br>Relevant for any intervention and any type of health problem. Any type of health condition may impair work participation at the most elemental level, having work	1. Having any type of employment including self-employment
**Return to work**<br><br>Interventions expected to help people who are absent from work due to a health problem return to work	1. Proportion of workers that return to work after being absent because of illness<br>2. Time to return to work in workers absent because of illness

DISCUSSION

Summary of main findings

Our international Delphi panel achieved consensus on three work participation outcomes for inclusion in COS for Work. As a minimum, all intervention studies addressing work participation should include *"any type of employment including self-employment"* as an outcome. Intervention studies which include participants who are absent from work due to a health problem should include the outcomes *"proportion of workers that return to work after being absent because of illness"* and *"time to return to work"*.

Strengths

We followed recommendations by the COMET initiative (10) to design the Delphi study, as well as the Core Outcome Set—STAndards for Development: The COS-STAD (8) and Core outcome set–STAndards for reporting: the COS-STAR.(9) For example, based on these standards we described the consensus definition *a priori* in a publicly available protocol. In addition, we used clearly communicated and specifically designed criteria for deciding which additional outcomes suggested by stakeholders would be suitable for COS for Work. Our systematic review provided us with a comprehensive overview of how work participation is measured and

For peer review only

reported internationally. We were able to cluster all the outcome concepts we found in the literature into twenty-four outcomes. Working with a relatively low number of outcomes is a facilitating factor for having focused discussions with stakeholders and may have contributed to the low attrition rate.(11)

The Delphi Manager software was designed by the COMET initiative to conduct Delphi studies.
We had a group of international stakeholders representing a wide range of expertise relevant for research in the field of occupational health. The online discussions provided valuable exchange of perspectives between the stakeholders. For instance, through sharing the information on the various social security systems it became clear why some outcomes would be less feasible to measure internationally.

Limitations

A limitation of the study is that we do not have core outcomes for all four work-participation stages. However, for some outcomes that did not meet the inclusion criterion, the consensus was still high and they are good candidates to be considered for measurement in studies (see Table 6).

Another limitation of this study is that we had difficultly recruiting employee and employer representatives. The employee group did not join the first online discussion. As our panel consisted mostly of researchers it is possible that views of the other four stakeholder groups were underrepresented. Nonetheless, as COS for Work is developed to be used by researchers we believe that it addresses issues researchers face at an elemental level. As we presented the work participation stages with relevant outcomes in order of most to least commonly measured (6) the “initiating employment” stage might have received less, and possibly not enough, attention.

Most participants were from Europe and North America. This might have influenced which outcomes are seen as most relevant for studies conducted on these continents and the results might be less applicable to low-and middle-income countries.

Results compared to previous findings

In terms of defining core outcomes for work participation most research to date has been done by the Outcome Measures in Rheumatology (OMERACT) initiative.(12) Their main focus is on worker productivity loss including outcomes such as absenteeism, presenteeism and costs, and focus on people with rheumatic diseases and musculoskeletal diseases.(13, 14) We consider that COS for Work is a suitable addition to include in studies which measure such productivity outcomes.

Sustainable employment was proposed as a potential COS outcome by a stakeholder during the first Delphi round. While we did not find this outcome as a commonly measured work outcome in the literature (6) it appears to be a highly important outcome from the employee perspective. Sustainable return to work is commonly measured in terms of having the ability to work for an extended period. However, employee representatives and some researchers understand sustainable employment in terms of quality of work, having decent work and a good work-life balance. Defining sustainability in terms of healthy work is in line with results of a study done among employees on how they perceive sustainable work.(15)

Implications for practice and research

In table 6 we present the candidate outcomes that did not meet the cut-off but were also highly ranked by all stakeholders. Two outcomes received low overall ratings but were ranked as “definite core outcome” by the employees: “workers’ perceptions on return to work” and “having lost employment” in the return to work stage. Further research is needed to investigate which of these outcomes should be prioritized under which circumstances.

Table 6: Candidate outcomes. *These outcomes were also high ranking (by at least one group) and need further investigation in which context they could be (additional) COS for Work outcomes.*

For peer review only

Stage of work participation — interventions and study populations for which outcomes are relevant within the stage	Potential (additional) future COS outcomes which did not meet the cut-point for COS for Work inclusion
**Stage 1: Initiating employment**<br><br>Interventions aiming to help unemployed people with distance to the job market due to a health problem get work	• Having any type of employment including self-employment
**Stage 2: Having employment**<br><br>Relevant for any intervention and any type of health problem. Any type of health condition may impair work participation at the most elemental level, having work	• Sustainable employment<br>• Work disability - self reported inability to engage in any work participation<br>• Employment – having lost employment
**Stage 3: Increasing or maintaining productivity at work**<br><br>Interventions expected to help workers who are not on sick leave to maintain or increase productivity at work despite a health problem	• Work productivity loss (economic)<br>• Work ability<br>• Work activity impairment
**Stage 4: Return to work**<br><br>Interventions expected to help people who are on sick leave return to work	• Sustainable return to work – proportion of workers<br>• Workers' perceptions on return to work<br>• Having lost employment

In our next study we will define and evaluate *how* to best measure 'having any employment' and 'return to work' in the COS for Work. Use of COS for Work by researchers will greatly help with evidence synthesis for researchers thereby helping decision making for practice and policy.

For peer review only

Acknowledgements

We would like to thank all the stakeholders who participated in this study for contributing to the results for COS for Work, the investment of time and the lively discussions. The following stakeholders have agreed to be publicly acknowledged as participants:

Aasdahl, Lene; Anema, Johannes; Berkowitz, Debbie; Bethge, Matthias; Bühler, Jonas; Bültmann, Ute; Böckerman, Petri; Coons, Trevor; Curti, Stefania; Crosse, Caroline; de Boer, Angela; Dorstyn, Diana; Foster, Nadine; Friberg, Emilie; Gehanno, Jean Francois; Godderis, Lode; Goodson, Nicola; Graveline, Christine; Gross, Douglas; Hara, Karen Walseth; Hannu, Timo; Hoff, Andreas; Hensing, Gunnel; Hegewald, Janice; Hoorntje, Alexander; Janssen, Svenja; Lacaille, Diane; Lam, Raymond; Landsbergis, Paul; Luiza Comper, Maria; Lytsy, Per; Macfarlane, Gary; Mantis, Steve; Merry, Kohle; Meyers, Alysha; Neupane, Subas; Nygård, Clas-Hakan; Oyeflaten, Irene Larsen; Pingle, Shyam; Prior, Yeliz; Schaafsma, Frederieke; Samant, Yogindra; Shaw, William; Snyder, Alexis; Steenstra, Ivan; Sturkenboom, Ingrid; Suijkerbuijk, Yvonne; Tsutsumi, Akizumi; Urquhart, Donna; van EE, Ilse; van Zaanen, Yvonne; Yamaguchi, Sosei; Zhang, Wei; Walker Bone, Karen

Funding

This research received no specific grant from any funding agency in the public, commercial or not-for-profit sectors. SV is supported by Versus Arthritis (grant numbers 20385, 20380) and the NIHR Manchester Biomedical Research Centre.

Contributors

All authors contributed to the conception and the study design. MR and JV analyzed the data. MR and JH assessed data quality. MR and JH tested the Delphi manager software prior to the first Delphi round. MR drafted the first version of the manuscript and all authors contributed to the review and editing process.

Competing interests

None declared.

Patient and public involvement

Patients and/or the public were involved in the design and conduct plans of this research as described in the Methods section.

Patient consent for publication

Not applicable.

Data sharing statement

All relevant data has been included in the article. Data are available upon request.

Ethics Approval

The research was conducted in accordance with the Declaration of Helsinki. The research proposal was submitted and approved by the Medical Ethical Committee of the Amsterdam UMC, Academic Medical Centre, Amsterdam, which judged that a comprehensive evaluation was not required since this study was not subject to the Netherlands' Medical Research Involving Human Subjects Act (reference: W22_129 # 22.171).

For peer review only

For peer review only

Reference list

1. Hoving JL, Lacaille D, Urquhart DM, et al. Non-pharmacological interventions for preventing job loss in workers with inflammatory arthritis. Cochrane Database of Systematic Reviews. 2014(11).
2. de Boer AG, Taskila TK, Tamminga SJ, et al. Interventions to enhance return-to-work for cancer patients. Cochrane database of systematic reviews. 2015(9).
3. Nieuwenhuijsen K, Faber B, Verbeek JH, et al. Interventions to improve return to work in depressed people. Cochrane Database of Systematic Reviews. 2014(12).
4. Kirkham JJ, Gargon E, Clarke M, et al. Can a core outcome set improve the quality of systematic reviews?–a survey of the Co-ordinating Editors of Cochrane Review Groups. *Trials*. 2013;14(1):21.
5. Williamson PR, Altman DG, Bagley H, et al. The COMET handbook: version 1.0. Trials. 2017;18(3):1-50.
6. Ravinskaya M, Verbeek JH, Langendam M, et al. Extensive variability of work participation outcomes measured in randomized controlled trials: a systematic review. *J Clin Epidemiol*. 2022;142:60-99.
7. Ravinskaya M, Verbeek JH, Langendam MW, et al. Preferred Methods of Measuring Work Participation: An International Survey Among Trialists and Cochrane Systematic Reviewers. *J Occup Rehabil*. 2022:1-9.
8. Kirkham JJ, Davis K, Altman DG, et al. Core outcome Set-STAndards for development: the COS-STAD recommendations. *PLoS Med.* 2017;14(11):e1002447.
9. Kirkham JJ, Gorst S, Altman DG, et al. Core outcome set–STAndards for reporting: the COS-STAR statement. *PLoS Med*. 2016;13(10):e1002148.
10. Williamson PR, Altman DG, Bagley H, et al. The COMET handbook: version 1.0. *Trials*. 2017;18(3):280.
11. Gargon E, Crew R, Burnside G, et al. Higher number of items associated with significantly lower response rates in COS Delphi surveys. *J Clin Epidemiol*. 2019;108:110-20.
12. Verstappen SM, Lacaille D, Boonen A, et al. Considerations for evaluating and recommending worker productivity outcome measures: an update from the OMERACT worker productivity group. *J Rheumatol.* 2019;46(10):1401-5.
13. Escorpizo R, Bombardier C, Boonen A, et al. Worker productivity outcome measures in arthritis. *J Rheumatol*. 2007;34(6):1372-80.
14. Gladman DD, Mease PJ, Healy P, et al. Outcome measures in psoriatic arthritis. *J Rheumatol*. 2007;34(5):1159-66.
15. Rooijackers B, de Rijk A, Horstman K, et al. A social ecology of sustainable employability: a qualitative study among Dutch employeesInge Houkes. *Eur J Public Health.* 2015;25(suppl_3).

For peer review only

PROTOCOL

REACHING CONSENSUS ON A CORE OUTCOME SET FOR WORK PARTICIPATION. A DELPHI STUDY.

Margarita Ravinskaya, Jos H. Verbeek, Miranda W. Langendam, Ira Madan, Suzanne M.M. Verstappen, Regina Kunz, Carel T.J. Hulshof, Jan L. Hoving

Date: 21 March 2022

INTRODUCTION

Authors of systematic reviews (SRs) state that inconsistent work participation outcome reporting in clinical trials hampers evidence synthesis in the field of occupational health (1-3). Co-ordinating Editors of Cochrane review groups indicate that reliability and quality of SRs could be improved by use of core outcome sets (4). In 2019, the Coronel Institute of Occupational Health at AMC Amsterdam established an international research collaboration to launch the development of a Core Outcome Set for Work Participation (COS for Work), based on the Core Outcome Measures in Effectiveness (COMET) Trials approach (COMET Initiative | Development of a Core Outcome Set for Work Participation (COS-WP) (comet-initiative.org))

The first phase of our research project involved a systematic review on the spectrum of work outcomes used to measure the effect of interventions in published trials (5). This review showed extensive heterogeneity in work participation outcome measurements and a need for a COS for Work. In the second phase we completed a survey among systematic reviewers and trial authors to gain insight in the reasons and preferences for choosing and using work outcomes (6). In addition, we created a framework to aid the selection of outcomes on of work participation (submitted). The framework outlines four main stages of work participation which can be used to group unique outcomes relevant for each stage. The stages are: (a) initiating employment, (b) having employment, (c) increasing or maintaining productivity at work and (d) return to employment.

In the current third phase we seek consensus on a comprehensive core set of work outcomes which can be used in all intervention studies that are expected to impact work participation directly or indirectly. In the fourth and final phase we will establish a list of validated outcome instruments/ measurable definitions for each of the core work outcomes.

The overall aim of this Delphi study is to define a minimal set of outcomes that are relevant, valid and feasible for measuring the effectiveness of interventions to achieve higher work participation, based on the consensus of an international, interdisciplinary and interprofessional stakeholder group.

For peer review only - http://bmjopen.bmj.com/site/about/guidelines.xhtml

METHODS

Consensus process

Steps of the consensus process

The consensus process will include five steps:

1. Preparation
2. First online consensus participant discussion (20 April 2022)
3. First round of Delphi voting (11-14 May 2022)
4. Second online discussion about the results of the first Delphi round (25 May 2022)
5. Second Delphi voting round (30 May 2022)

1. Preparation

The objective of this step: prepare the participants for the first online discussion.

Two groups participate in this study:

1. The international COS steering group with researchers from four countries:

   Jan Hoving (NL) , Margarita Ravinskaya (NL), Carel Hulshof (NL), Suzanne Verstappen (UK), Ira Madan (UK), Regina Kunz (CH), Miranda Langendam (NL), Jos Verbeek (FI)

2. Study participants.

   Five types of international stakeholders will be recruited for the consensus process:

   1. Researchers in occupational health with experience in RCT/SR and / or cost-effectiveness studies
   2. Health professionals in occupational health and insurance medicine
   3. Representatives of workers (e.g. union members)
   4. Representatives of employers (e.g. members of business federations)
   5. Representatives of policy makers (e.g. government representatives, members of EU OSHA or the World Health Organization)

Participants may also fulfill multiple roles (for example be a researcher and health professional) and share their knowledge and experience as input during the discussion rounds. During the Delphi rounds, however, the participants must choose one stakeholder group that they wish to represent. We will assign the stakeholder roles to every participant based on their background and confirm prior to the first discussion round if they agree. The majority of the participants will be researchers because their experience with measuring work participation will be helpful in making informed decisions. We arranged this composition of stakeholders to provide a diversity of views from the health, societal, worker and employer perspectives. There are no set guidelines on the number of participants for the consensus on core outcome sets. A study by Signha et al. that the number of participants to range from 13 to 222 (7). We will recruit about 100 participants with the aim to have 50 participants take part in the consensus.

For peer review only - http://bmjopen.bmj.com/site/about/guidelines.xhtml

We will recruit potential via the following channels:

1. Invite researchers who have responded to our survey (6) and indicated that we could contact them in the future
2. Invite researchers and other stakeholders through our international network

We will identify the potential participants based on their professional background and the geographical location. Participants living in Europe, North and South America, Asia, Australia and Africa will be invited. An e-mail will be sent out specifying the aims of the project, the required input and dates of the plenary discussions and the Delphi rounds.

Using the results from the systematic review, the survey and the new framework, the steering group will write a concise background document for participants attending the two rounds of the consensus process. This includes information on the COS for Work framework, the various work participation outcomes and definitions used in the literature and our proposed definitions for the different outcomes which will be used in the Delphi voting rounds. The participants are asked to reserve approximately two hours to prepare for the consensus process by reading/familiarizing themselves with the background document. In addition they are asked to provide written feedback about any outcome which is not part of our preliminary set but they would find potentially core with their reasoning behind it.

<u>2. First online consensus participant discussion</u>

The objectives of the online discussion are as follows:

1. Present the COS for Work framework with the four stages of work participation: (1) initiating employment, (2) having employment, (3) increasing or maintaining productivity at work, and (4) return to employment
2. Make explicit that within each stage of work participation there are various outcome concepts and we are seeking consensus on <u>core outcomes</u> belonging to each category.
3. Give an example of a COS for Work: core outcomes for every stage of work participation.
4. Ensure that all participants understand their roles and their tasks for the Delphi voting
5. Discuss which are the most important outcomes from the preliminary for every group of stakeholders
6. Present any additional core outcomes we received from the stakeholders (if more than two stakeholders provided they via mail prior to the discussion)

The discussions will take two hours per group. We will divide the participants in two groups to facilitate participation across all times zones. The first group will be from 09:00 till 11:00 CET and include participants from Europe, Asia and Australia. The second group will be from 17:00 to 19:00 CET and include participants from Europe, Canada, North America and South America.

The participants will receive a Zoom link. In the e-mail invitation we will state that the session will be recorded unless anyone wishes to object. We will discuss the topics as outlined in the aims of the discussion using the background document. Participants will have the opportunity to provide input on each topic. The chair, Carel Hulshof, will facilitate the discussion and the other members of the steering group will facilitate the chat and the poll functions. The chair will work with a meeting

For peer review only - http://bmjopen.bmj.com/site/about/guidelines.xhtml

manual prepared by the steering group. The results of this online discussion will serve as input for the first Delphi round.

3. First round of Delphi voting

Objective: get a preliminary agreed upon set of core outcomes for each stage of work participation

If for some reason participants were not able to attend the first discussion round they may take part in the Delphi voting if they familiarized themselves with the background document. Any written feedback will be taken into consideration by the steering group as input for the Delphi voting.

The list of the outcomes for every stage of work participation will be agreed upon during the discussion. If more than one stakeholder wants to add an outcome which was not part of the preliminary set we will add the outcome to the Delphi rounds.

Voting procedures and interpretation of the findings:

Participants will receive a link to register as a stakeholder for the DelphiManager software (provided by COMET) and vote on which categories and outcomes within the four stages of work participation they deem as core outcomes.

Quantitative data will be collected and provided to participants to inform the ranking of outcomes. The DelphiManager includes ranking based on a Likert Scale from 1-9; from not important to critical. An "unable to score" option is also given.

Consensus on outcomes to include in the core outcome set is when ≥ 80% of participants score an outcome as critically important (score 7 to 9) and consensus to not include an outcome as core is when <50 % of participants score outcomes with 7-9. However, it is possible that too many outcomes may have consensus to include for a core outcome set and some important outcomes may receive low scores due to lack of awareness of their importance by the majority of stakeholders. Therefore we will use the consensus definition, as described above, as a guideline and provide the participants with the opportunity to elaborate on their choices during the second discussion.

4. Online discussion of the results of the first Delphi round

The aim of the second discussion will be to go through the results of the first voting round. Outcomes with most agreement (score 7 to 9) will be presented and discussed. Clarifications of the outcomes and opinions on why certain outcomes deserve priority will be discussed. Any strong arguments from stakeholders against high scoring outcomes and potential "downgrading" of high scoring outcomes will be discussed. Outcomes which were not ranked as critical (<50 % of participants score outcomes with 7-9) will also be discussed and the participants will provide input whether all or some these outcomes can be dismissed from the second round of voting. Based on this discussion the steering group will consider the argumentation of the stakeholders and decide which outcomes will be included in the second Delphi round.

For peer review only - http://bmjopen.bmj.com/site/about/guidelines.xhtml

5. Second Delphi voting round

The objective of the second Delphi round will be to reach agreement by stakeholders on the final set of core outcomes for each stage of work participation. In the second Delphi round the participants will see the scores from other participants on every outcome within each of the four stages and re-rate the outcomes which were considered critical after the first round of voting and the second discussion round.

DATA ANALYSIS

The data from the Delphi rounds will be processed anonymously but in the discussion rounds the participants will be identifiable.

Group discussions

We will summarize the main take-aways from the group discussion on every topic and hold a meeting with the steering group on how we will process the input for the Delphi study. For the topics which require voting, at least 70% agreement will be required for an argument to be considered as a "yes".

Delphi voting rounds

The results from the Delphi rounds will be extracted from the Delphi Manager software into a CSV format and analyzed in SPSS using descriptive statistics. We will report measures of the distribution scores for all outcomes which will be included in the final round. If too many outcomes will have agreement on inclusion, outcomes with the highest mean scores within each stage of work participation will be selected as core.

RESULTS

Results will be processed anonymously. Qualitative data from the discussions with the stakeholders will be summarized. Quantitative data from the voting rounds will include distributions of the scores for all outcomes and the number of participants who scored the outcomes.
In the following project the steering group will give recommendations on how to best measure the selected core outcomes. The results will be reported in an article to be submitted to an international peer reviewed journal. After the publication we will share the results with the participants and disseminate further within the research community.

For peer review only

Reference list

1. Hoving JL, Lacaille D, Urquhart DM, Hannu TJ, Sluiter JK, Frings-Dresen MH. Non-pharmacological interventions for preventing job loss in workers with inflammatory arthritis. Cochrane Database of Systematic Reviews. 2014(11).
2. de Boer AG, Taskila TK, Tamminga SJ, Feuerstein M, Frings-Dresen MH, Verbeek JH. Interventions to enhance return-to-work for cancer patients. Cochrane database of systematic reviews. 2015(9).
3. Nieuwenhuijsen K, Faber B, Verbeek JH, Neumeyer-Gromen A, Hees HL, Verhoeven AC, et al. Interventions to improve return to work in depressed people. Cochrane Database of Systematic Reviews. 2014(12).
4. Kirkham JJ, Gargon E, Clarke M, Williamson PR. Can a core outcome set improve the quality of systematic reviews?–a survey of the Co-ordinating Editors of Cochrane Review Groups. Trials. 2013;14(1):21.
5. Ravinskaya M, Verbeek JH, Langendam M, Daams JG, Hulshof CT, Madan I, et al. Extensive variability of work participation outcomes measured in randomized controlled trials: a systematic review. Journal of Clinical Epidemiology. 2022;142:60-99.
6. Ravinskaya Margarita; Verbeek JHL, Miranda; Hulshof, Carel TJ; Madan, Ira; Verstappen, Suzanne MM; Kunz, Regina; Hoving, Jan L. Preferred methods of measuring work participation. An international survey among trialists and Cochrane systematic reviewers. "In press". 2022.
7. Sinha IP, Smyth RL, Williamson PR. Using the Delphi technique to determine which outcomes to measure in clinical trials: recommendations for the future based on a systematic review of existing studies. PLoS medicine. 2011;8(1):e1000393.

For peer review only

Development of a core outcome set for work participation:

COS for Work

Introduction on COS for Work and instructions for the Delphi study

Steering group

Dr. Jan L. Hoving - Amsterdam UMC, location Academic Medical Center, University of Amsterdam, Coronel Institute of Occupational Health, Amsterdam Public Health research institute, Amsterdam, NL

Margarita Ravinskaya - Amsterdam UMC, Academic Medical Center, University of Amsterdam, Coronel Institute of Occupational Health, Amsterdam Public Health research institute, Amsterdam, NL

Dr. Jos H. Verbeek MD - Amsterdam UMC, location Academic Medical Center, University of Amsterdam, Coronel Institute of Occupational Health, Amsterdam Public Health research institute, Amsterdam, NL

Dr. Miranda W. Langendam - Amsterdam UMC, location Academic Medical Center, University of Amsterdam, Department Epidemiology and Data Science, Amsterdam Public Health research institute, NL

Prof. Ira Madan, MD, FRCP, FFOM – Guy's and St Thomas' NHS Trust and Faculty of Life Sciences and Medicine, King's College London, Centre for Musculoskeletal Health and Work, UK

Prof. Dr. Regina Kunz, MD - Research Unit EbIM, Evidence Based Insurance Medicine, Department of Clinical Research, University of Basel, CH

Dr. Suzanne M.M. Verstappen - [1] Centre for Epidemiology Versus Arthritis, Faculty of Biology, Medicine and Health, The University of Manchester, UK, [2] NIHR Manchester Biomedical Research Centre, Manchester University NHS Foundation Trust, Manchester Academic Health Science Centre, UK, [3] MRC Versus Arthritis Centre for Musculoskeletal Health and Work, University of Southampton, UK

Prof. Dr. Carel T.J. Hulshof, MD - Amsterdam UMC, location Academic Medical Center, University of Amsterdam, Coronel Institute of Occupational Health, Amsterdam Public Health research institute, Amsterdam, NL

1. Introduction

1.1 What is this document about?

You have accepted our invitation to take part in the consensus process to establish a core outcome set for work participation (COS for Work). Thank you for taking the time to collaborate with all of our international colleagues in the field of occupational health and related areas. In this document we explain the need for and purpose of COS for Work, how the consensus process will work, and what is expected of you.

1.2 What is a COS?

A COS is an agreed *minimum* set of outcomes that should be measured consistently in all studies in a specific health area to make study findings on similar topics more comparable. These core outcomes are chosen and defined by key stakeholders, for example health professionals, researchers, patient population and policy makers.

Core outcome sets have the following benefits: (1) facilitate large scale evidence synthesis by systematic reviews, (2) provide clearer answers to the question of what works best, (3) support informed decision making in clinical practice and policy, (4) reduce reporting bias and research waste, and (5) ensure that the outcome

For peer review only - http://bmjopen.bmj.com/site/about/guidelines.xhtml

measures selected are truly critical to key stakeholders[1] . A common misconception about the use of core outcome sets is that studies should *only* include the outcomes from the COS. This is not the case: rather, any other primary and secondary outcomes can be measured *alongside* the outcomes from a specific COS.

The current heterogeneity in measuring work participation (WP) hinders a full understanding of the findings across similar studies. A small set of WP outcomes used in all studies would close this gap. As of date such a generic set of study outcomes is lacking. This project aims to create a COS for Work. We consider this project to be a highly relevant contribution to research methodology of occupational health. It is a starting point. Further iterations will refine and improve the COS over time.

1.3 The purpose COS for Work

Even though COS can be developed for practice and/or research, this COS for Work is intended for research purposes only. The aim is to assist researchers in choosing which outcomes to measure in effectiveness studies to improve WP for people with health problems. Our COS for Work will be a generic COS (in contrast to COS that are developed for a specific disease). This means, it intends to evaluate any type of WP intervention in people with any type of health problem.

In general, two types of interventions can be distinguished: (1) vocational interventions with direct impact on work participation, such as Individual Placement and Support studies, and (2) non-vocational interventions, i.e. any type of clinical, pharmacological, psychological study with an intervention to improve health. For non-vocational interventions the effect on work participation is often a secondary objective and work outcomes are measured as secondary outcomes.

1.4 Our preparatory studies for COS for Work

To lay the groundwork for the consensus process, we conducted three studies. The full text versions are attached to the e-mail. Reading is optional but highly recommended. Below is a summary of the aims and findings of the three papers.

1. **Extensive variability of work participation outcomes measured in randomized controlled trials: a systematic review. https://doi.org/10.1016/j.jclinepi.2021.10.013 – Published**
We performed a systematic review which showed how WP outcomes are measured across randomized controlled trials (RCTs) aimed to impact WP directly or indirectly. We found a great deal of heterogeneity on the level of outcome terminology and measurement methods. We grouped outcomes in four main categories: “employment status”, “absence from work”, “at work productivity loss” and “employability”.

2. **Preferred methods of measuring work participation. An international survey among trialists and Cochrane systematic reviewers. https://doi.org/10.1007/s10926-022-10031-0 - Published**
We contacted the authors of the RCTs included in the above review and the authors of Cochrane systematic reviews to explore their reasons for choosing specific outcomes and measurement methods. Our findings show that the authors based their choice of measurement methods mainly on previous similar studies, that feasibility is the most important factor in the choice of measurement method, and that a COS for Work is considered very useful for research. In addition, the authors indicated that the most important outcomes for a COS for Work would be “employment status” and “sickness absence” and “return to work”. Their preferred follow-up period was 12 months across all outcome categories.

[1] Williamson PR, Altman DG, Bagley H, Barnes KL, Blazeby JM, Brookes ST, et al. The COMET handbook: version 1.0. Trials. 2017;18(3):280.

For peer review only

3. **A general framework for selecting work participation outcomes in intervention studies among persons with health problems.** [https://doi.org/10.21203/rs.3.rs-1342930/v1 - Under review](https://doi.org/10.21203/rs.3.rs-1342930/v1)
As work participation is an umbrella term containing different types of outcomes, we looked for a comprehensive framework that could be used for COS for Work. Since there was no pre-existing framework, we developed one for COS for Work. The framework includes the following four stages of WP: (1) initiating employment, (2) having employment, (3) increasing or maintaining productivity at work and (4) return to employment. These work participation stages should help our consensus process to select the outcome concepts and outcome measures. Furthermore, we defined criteria for COS outcomes that are part of the document for selecting outcomes in the Delphi study.

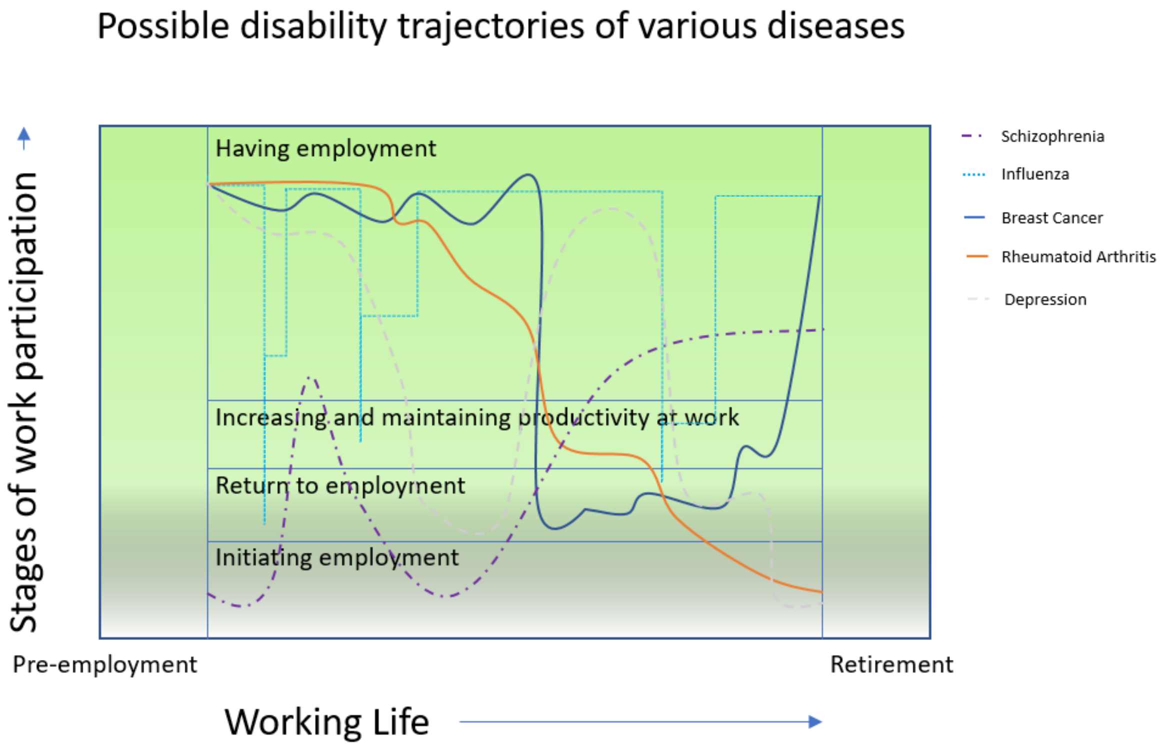

This figure is adapted from the COS framework. Here we illustrate how possible disability trajectories result from various health problems. The four work participation stages can be experienced during the course of the working life.

When linking the 24 commonly used outcomes to the four stages, 10 outcomes referred to 'Return to employment', 5 outcomes to 'Increasing or maintaining productivity at work', 7 outcomes to 'Having employment' and 2 outcomes to 'Initiating employment'. Many of these outcomes can be refined in more details, e.g. by specifying the timing (1 day, 4 weeks, 12 months) or type or amount of work (full time, part-time, last job, any work).

2. Instructions for the Delphi Study

<u>2.1 Purpose of the study</u>

The purpose of this consensus study is to identify a core set of outcomes (COS) that captures work participation and that can be used in intervention studies at each of the four stages of work participation

identified in our framework. Please note that discussion and votes in this study do not address measurement instruments.

The study includes 4 activities: Two online consensus discussions and two rounds of Delphi voting.

1. First online group discussion (20 April 2022)
2. Delphi I voting (11 – 14 May 2022)
3. Second online group discussion about the results of the first Delphi vote (25 May 2022)
4. Delphi II voting (30 May – 1 June)

We seek a minimum set of outcomes that are meaningful and easy to measure ('feasible') by all studies in the field. There are no strict rules for the number of outcomes a COS may contain, but the current 24 outcomes would defeat the purpose. Compromises may need to be made in the number and type of outcomes.

2.2 Organization

a) **Selection of participants**

To cover core outcomes relevant to a broad range of stakeholders, we invited experts from diverse professional groups. We have assigned you to one of the following stakeholder groups: researcher, policy maker, employer representative, employee representative, occupational health professional (see document "COS Delphi participants_ stakeholder groups"). Please use the perspective of your assigned stakeholder group in your country when discussing and selecting the core outcomes. Some of you fulfill more than one role in practice or have done so in the past. If for any reason you disagree with the group you have been assigned to, please let us know.

b) **Running the meeting**

Chair: Prof. Carel Hulshof will chair the meetings, and the COS for Work team will assist the process.

Protocol: To be transparent on how consensus is reached, we are working with a protocol that is publicly available on the COMET website (https://www.comet-initiative.org/studies/details/1195).

Video recording: We will record the online meetings for study purposes only. The recordings will be destroyed before 31 December 2022.

c) **Ground rules for communication**

All stakeholders should have the opportunity to voice their opinion. As the time for discussion will be limited, please try to present your arguments briefly and succinctly so that others can also put forward their arguments, thus facilitating the exchange.

d) **Contact for queries**

If anything is unclear, please do not hesitate to contact us.

Contact: Margarita Ravinskaya, m.ravinskaya@amsterdamumc.nl

2.3 The activities

1. First online group discussion

When: 20 April 2022

Zoom link

Connect to the discussion via the following link: https://uva-live.zoom.us/j/9294218837

Aims of the session

For peer review only

1. Ensure that all participants understand their roles and their tasks for the discussions and the Delphi votes.
2. Present the COS for Work framework.
3. Make explicit that each stage of work participation has more than one outcome concept. We are seeking consensus on outcome concepts and the core outcomes belonging to each stage for most stakeholders.
4. Present additional core outcomes that may have been submitted by at least two stakeholders.
5. Discuss the most important outcomes from the preliminary outcome set (document "Delphi voting: Stages and Outcomes"), first in breakout rooms and finally in plenary setting.

Preparatory work for the participants

1. Familiarize yourself with the document "Delphi voting: Stages and Outcomes" (see email attachment) to be used for the Delphi rounds. The preliminary list of outcomes per WP stage are based on the outcomes most commonly measured in our systematic review (see email attachment). If you miss crucial outcomes, please send us those outcomes and your reasons, as soon as possible but 18 April 2022 at the latest.
2. Write your preliminary selection of core outcomes in the worksheet table in the document. Please specify the minimum of outcomes that you think should be included in COS for Work.

   In this project, we aim to achieve consensus on outcomes only (not on measurement instruments). While choosing your preferred core outcomes, please write down your rationale for doing so. For example, in our survey reasons for choosing a certain outcome were their use in similar studies, relevance for the study population, or easy access to registry data. Discussing and balancing preferences for specific outcomes will facilitate the final selection.

Definition "Outcome Measure" versus "Measurement Instrument"

Please note the difference between "outcome concept", "outcome measure" and "measurement instrument" (or tool or method). "Outcome concept" is an umbrella term for the many facets of a certain concept. *An outcome is WHAT we aim to measure. A measurement instrument is the method HOW the outcome is measured.*
The Delphi voting will refer to the colored columns only (NO VOTING on the measurement instrument)

Outcome concept	Outcome measure (Description)	Measurement instrument (Example)
Work ability	Current self-rated physical and mental work ability compared to lifetime best	The Work Ability Index

3. Recommended but optional: familiarize yourself with our three background studies (systematic review, survey, and framework, see email attachment).

2. Delphi I: Individual online voting

When: 11 – 14 May 2022

Individual assignment for all participants

Use the DelphiManager software to select and rank outcomes and to motivate your selection and ranking. Please complete this task between 11 and 14 of May 2022.

Overall group aim:

For peer review only - http://bmjopen.bmj.com/site/about/guidelines.xhtml

- Reach an initial consensus on a set of core outcomes for each stage of work participation.

The DelphiManager software

We will use the DelphiManager software to vote on the outcomes for the four stages of WP. You will receive a link to register for the DelphiManager software.

The DelphiManager includes a ranking of the outcomes on a Likert Scale from 1-9: from 'not important' to 'critical', plus an 'unable to score' option. The results will be summarized and reflected in the second discussion round and a refined set of outcomes will be presented in the second Delphi vote.

3. Second online group discussion

When: 25 May 2022

Zoom link

Connect to the discussion via the following link: https://uva-live.zoom.us/j/9294218837

Overall group aim:

- Discuss the results of the first Delphi voting round with other stakeholders.

We will discuss the outcomes with the highest agreement (score 7 to 9) and the rationale on why certain outcomes deserve priority. Any strong arguments from stakeholders against high scoring outcomes and potential "downgrading" of high scoring outcomes will be discussed. Outcomes not ranked as critical (<50 % of the participants scored the outcomes with 7-9) will be reviewed to determine whether some or all of these outcomes can be dismissed from the second round of voting. Based on the arguments of the stakeholders in this discussion, the COS for Work team will make the final decision on which outcomes to include in the second Delphi vote.

4. Delphi II : Individual online voting

When: 30 May – 1 June 2022

Overall group aim:

- Reach consensus on the final set of core outcomes for each stage of work participation.

In the second Delphi voting round, participants see the scores of the other participants for each outcome in all four stages. Using DelphiManager software, you will re-rate all outcomes that were considered critical after the first Delphi round of voting and the second round of discussion.

For peer review only

For peer review only

Introduction

In this document we present a list of work participation outcomes measured randomized controlled trials. The outcomes are sorted according to the stage of work participation.
The stages are presented in the order of most to least commonly measured outcomes.

First, you will find a brief explanation of every stage. In particular we describe for which target group and intervention types outcomes belonging to each stage are relevant. We also provide some additional information for every stage. The “criteria to consider for COS outcomes” were developed as part of the framework. They are mostly important on the level of measurement. We will use these criteria after the Delphi study to determine how each COS outcome resulting from this Delphi study can be measured. We include the criteria for your information, so you may take them into consideration (as far as possible) when selecting most important outcomes.

For every stage you will find a preliminary set of outcomes. The “outcome concept” refers to a category of outcomes within each stage. The “outcome measure” is the list of outcomes which will be used to vote on in the Delphi rounds. In addition, we provide examples of measurement tools, instruments or methods.

Please consider which outcome measures you find most important and complete the worksheet belonging to every stage. It will be helpful if you also note the outcome ID you are referring to. This will be the basis of our first round of Discussion on 20 April 20 2022.

If you miss an important outcome concept or outcome measure, please send us the outcome and the reason of its’ importance. **Please do so 18 April 2022 at the latest**, in that way we can include the additional outcomes in the first discussion on 20 April.

Stage ‘Return to Employment’ & Sick leave from work

This is a stage of work participation when employed people (temporarily) stop attending work and are on sick leave. The ICF does not include sick leave or sickness absence, but potentially outcomes for this stage could fit under “maintaining employment” and “remunerative employment” ICF categories. Work related rehabilitation interventions designed to help workers return to work include outcomes relevant to this stage as their primary outcome. Any type of clinical, pharmaceutical or otherwise health related intervention may be expected to indirectly impact sick leave. Sickness absence can show that a person is unable to fulfil their worker role due to ill health, but other reasons for absenteeism, such as maternity leave or unwillingness to come to work also exist. Evaluation of absenteeism outcomes is often approached from the societal/economic perspective. Sick leave is often converted into a monetary value as part of economic evaluations to indicate extra costs for the employer or/and the employee.
Measuring changes in sick leave is not straightforward as the denominator *can refer to all workers or to workers that are on sick leave*. It is, therefore, very important to be clear to which group the sick leave figures refer.
More recently, the term ‘return to work’ has been introduced as an indicator of sickness absence from an individual perspective. For individuals, the worker role is important for economic reasons but also for reasons of well-being. For example, cancer survivors report that getting back to work is a final step in getting back to normal life after their disease/treatment experience.

Additional information to consider for this stage

- Outcomes within this stage of work participation are measured most frequently (45% of all RTC’s in our review) in intervention studies which directly or indirectly aim to impact work participation
- In our survey, 93% of RCT authors and 69% of Cochrane systematic reviewers indicated that outcomes belonging to this stage should be in COS for Work
- Registry data is preferred by researchers, but type and definition of sickness benefits registry data varies per country and may be inaccurate about the diagnosis

- Time to RtW as a standalone measure may not be a meaningful outcome about sustained effect of an intervention (recommendations are made to also measure duration of sick leave, sick leave spells and proportion of employees who return to part/full time work)

Criteria for COS outcomes

Criteria to consider	***Optional criteria***
Outcomes should:<br>1. Be sensitive to change<br>2. Be feasible to measure<br>3. Be applicable internationally<br>4. Be work participation specific<br>5. Capture multiple stakeholder perspectives<br>6. Be in alignment with the ICF model	*Outcomes should:*<br>1. Be used for cost-effectiveness studies<br>2. Be applicable across varying insurance schemes

**ID**	**Outcome concept**	**Outcome measure (description)**	**Measurement instrument (examples)**
1.1.1	Return to work	*Proportion of workers that return to work* after being absent because of an illness	- to any type of work<br>- to specific type of work<br>- to a defined amount of work<br>- at a defined point in time (e.g. at least 1 day, 3 days, 4 weeks, 12 months)
1.1.2		*Time to return to work* or duration of sickleave after being absent because of an illness	- to any type of work<br>- to specific type of work<br>- to a defined amount of work<br>- at a defined point in time (e.g. at least 1 day, 3 days, 4 weeks, 12 months)
1.1.3	Sustainable Return to work	*Proportion of workers that return to work and remain at work for a specified amount of time*	e.g., 4 weeks without relapse of sick leave at a specific moment of follow-up e.g. 12 months
1.1.4		*Time to return to work in workers absent because of an illness and who do not relapse within a specified amount of time*	e.g., 4 weeks at a specific moment of follow-up e.g. 12 months
1.1.5	Return to work: the workers perceptions	*Time it will take to return to work as perceived by the sick worker;* also described as self-efficacy for return to work, return to work expectations or intention to return to work	- Work Readiness Questionnaire (WoRQ)<br>- Inventory for Job Coping and Return Intention (JoCoRi)

1.2.1	Sickness absence *among all workers* in an organization/group	*A. Lost working days*<br>*B. Lost sick days*	A. Percentage of all working days of all workers in an organization during a time period e.g. 12 months<br>B. Sick days as percentage of all available days during a time period
1.2.2		*Sick leave duration*	- Working days absent because of illness averaged overall workers<br>- Calendar days sick
1.2.3		*Sick leave frequency*	Number of episodes of sick leave per worker expressed as percentage

1.2.4	Sickness absence *among absent workers* in an organization/group	*a) Lost working days*<br>*or*<br>*b) lost sick days*	a) percentage of all working days during a time period e.g. 12 months;<br>b) sick days as a percentage of all available days during a time period
1.2.5		*Sick leave duration*	• working days absent averaged over all sick leave episodes; can also be expressed as calendar days sick;<br>• is numerically identical to *time to return to work* among workers absent from work

Worksheet for participants

**Outcomes measures for 'Return to Employment'**	**Reasons for selecting these outcomes** *(Examples: previously used in similar studies, relevance for the study population, easy access to data, e.g. registry data, ...)*
**Step 1: Select all outcomes that you feel are important**	
a.	
b.	
c.	
d.	
e.	
f.	
g.	
**Step 2: Note the outcomes that you consider to be the bare minimum for COS for Work**	
a.	
b.	
c.	
d.	
e.	
f.	
g.	

Stage 'Increasing or maintaining productivity at work'

This stage of work participation occurs when people experience limitations or impairments whilst working which results in lower quality or lower output of work. The input versus output is often used to calculate cost. In terms of ICF, the outcomes could be placed under the ICF-category "Maintaining a job".
Outcomes measure relevant for this stage are for any type of vocational or clinical/pharmaceutical intervention that may impact on overall productivity, work ability and work functioning of people with a health problem.
From the worker perspective, feeling fit to work is essential for a successful worklife. Employers can offer interventions to reduce stress and increase wellbeing in the workplace as preventive measures.

Additional information to consider for this stage

- Outcomes for this stage were measured second most frequently in the review; 30% of all RCTs measured an outcome relating to productivity.
- 63% of the RCT authors and 75% of Cochrane reviewers indicated in the survey that measuring outcomes for this stage should be part of the core outcome set.

Criteria for COS outcomes

Criteria to consider	***Optional criteria***
Outcomes should:<br>1. Be sensitive to change<br>2. Be feasible to measure<br>3. Be applicable internationally<br>4. Be work participation specific<br>5. Capture multiple stakeholder perspectives<br>6. Be in alignment with the ICF model	*Outcomes should:*<br>1. Be used for cost-effectiveness studies<br>2. Be applicable across varying insurance schemes

**ID**	**Outcome concept**	**Outcome measure (Description)**	**Measurement instrument (Examples)**
2.1.1	Work productivity loss (economic evaluation)	Overall loss of productivity at work (in terms of quality or output at work) resulting from presenteeism or absenteeism regardless of cause.<br>Typically used for economic evaluations	Work Productivity and Activity Impairment Questionnaire
2.2.1	Presenteeism	Not being productive while at work due to health problems.<br>Typically used to evaluate the effect of health interventions for individuals	Stanford Presenteeism Scale
2.3.1	Work ability	Current self-rated physical and mental work ability compared to lifetime best. Often used for prognosis of RtW, however the commonly used tool (WAI) is not effective for identifying changes of work ability after RtW	Work Ability Index (WAI)
2.4.1	Work activity impairment	Experienced functional impairment of performing work activities due to a health problem.	Work Limitations Questionnaire (e.g. WLQ 25)
2.5.1	Perceptions affecting productivity	Beliefs in working capacity, motivation for work, vocational commitment, job coping, contentment with work, need for recovery	- Subjective Prognosis of Work Capacity<br>- Work Motivation Scale<br>- Need for Recovery Scale<br>- Job Coping (JoCoRi))

Worksheet for participants

**Outcomes measures for 'Increasing or maintaining productivity at work**	**Reasons for selecting these outcomes**<br>*(Examples: previously used in similar studies, relevance for the study population, easy access to data, e.g. registry data, ...)*
**Step 1: Select all outcomes that you feel are important**	
a.	
b.	
c.	
d.	
e.	
f.	
g.	
**Step 2: Note the outcomes that you consider to be the bare minimum for COS for Work**	
a.	
b.	
c.	
d.	
e.	
f.	
g.	

Stage 'Having employment'

Outcomes relevant for this stage of work participation measure whether a person is in employment, can retain employment, loses work or retires within the duration of the study. In general, these outcomes fit in the ICF categories under "Remunerative Employment" and "Keeping a Job". Being employed can be considered as having a contract with an employer or being self-employed. Both, vocational or non-vocational interventions, are relevant for this stage. Most studies on vocational rehabilitation have the primary aim to determine the effect of an intervention on employment status. Medical or pharmaceutical studies are more likely to measure the effect of the intervention on employment status as secondary outcome. The target group of these interventions are people who are known to be at risk of losing employment due to a health problem.

Additional information to consider for this stage

- Our review showed that 16% of all measured outcomes belonged to this stage.

- 98% of RCT authors and 100% of Cochrane reviewers agreed that outcomes for this stage of work participation should be part of COS for Work.
- Most of the measurement tools used to measure outcomes at this stage are not validated, such as unspecified (self-developed) items, interviews, and phone calls. The exception is data from registries such as those in the Nordic countries.

Criteria for COS outcomes

Criteria to consider	***Optional criteria***
Outcomes should:<br>1. Be sensitive to change<br>2. Be feasible to measure<br>3. Be applicable internationally<br>4. Be work participation specific<br>5. Capture multiple stakeholder perspectives<br>6. Be in alignment with the ICF model	*Outcomes should:*<br>1. Be used for cost-effectiveness studies<br>2. Be applicable across varying insurance schemes

**ID**	**Outcome concept**	**Outcome measure (Description)**	**Measurement instrument (Examples)**
3.1.1	Employment	Having any type of employment including self-employment	
3.1.2	Employment	Having met a pre-defined status of employment; part/full time, competitive work in a mainstream setting, performing the same tasks as non-disabled workers	
3.1.3	Employment	Predefined type or amount of income	
3.1.4	Employment	Duration of employment	
3.2.1	Employment	Having lost employment	
3.3.1	Work disability	Permanent and complete inability to engage in any work participation	
3.3.2	Work disability	Permanent partial disability to engage in work participation	

Worksheet for participants

**Outcomes measures for 'Having employment'**	**Reasons for selecting these outcomes**<br>*(Examples: previously used in similar studies, relevance for the study population, easy access to data, e.g. registry data, ...)*
**Step 1: Select all outcomes that you feel are important**	
a.	

b.	
c.	
d.	
e.	
f.	
g.	
**Step 2: Note the outcomes that you consider to be the bare minimum for COS for Work**	
a.	
b.	
c.	
d.	
e.	
f.	
g.	

Stage: Initiating employment

Outcomes within this stage measure whether participants are prepared for initiating employment. The target group are people who are unemployed and seeking employment (i.e., not self-employed or contracted by an employer, however, possibly with a type of subsidized governmental wage replacement benefit). This stage is particularly applicable to those who never worked, for example due to just completed education. ICF categories "Apprenticeship" (work preparation) and "Acquiring a job" apply. Interventions for this category focus on increasing skills, knowledge or attitude of participants for successful engagement in a workers' role.

Additional information to consider for this stage

- The outcomes belonging to this stage were measured least frequently in our review, in about 5% of all trials.
- In our survey, 59% of RCT authors and 75% of Cochrane reviewers indicated that outcomes from this category should be part of the COS.
- These outcomes have been shown to be a predictor for employment status.

Criteria for COS outcomes

Criteria to consider	***Optional criteria***
Outcomes should:	*Outcomes should:*

Criteria to consider	*Optional criteria*
7. Be sensitive to change<br>1. Be feasible to measure<br>2. Be applicable internationally<br>3. Be work participation specific<br>4. Capture multiple stakeholder perspectives<br>5. Be in alignment with the ICF model	1. Be used for cost-effectiveness studies<br>2. Be applicable across varying insurance schemes

ID	Outcome concept	Outcome measure (Description)	Measurement instrument (Examples)
4.1	Skills for job procurement	Total work-related network, Engagement in work seeking activities, Job interview skills, Work-related benefit of vocational training	- Work section of the Social Adjustment scale<br>- Mock Interview Rating Scale
4.2	Self-efficacy	Self-efficacy for job procurement, Hope, optimism and self-efficacy for achieving vocational success, Social/interpersonal self-efficacy	- Job Procurement Self-efficacy Scale<br>- The Work Hope Scale<br>- Subjective Prognosis of Work Capacity

Worksheet for participants

**Outcomes measures for 'Initiating employment'**	**Reasons for selecting these outcomes**<br>*(Examples: previously used in similar studies, relevance for the study population, easy access to data, e.g. registry data, ...)*
**Step 1: Select all outcomes that you feel are important to include in COS for Work**	
a.	
b.	
c.	
d.	
e.	
f.	
g.	
**Step 2: Note the outcomes that you consider to be the bare minimum for COS for Work**	
a.	
b.	

c.	
d.	
e.	
f.	
g.	

For peer review only

For peer review only

Suggested outcome(s)	Stage	Argumentation	Other remarks
**"Work environment" Availability/access to appropriate work modifications/supports**	Increasing or maintaining productivity	This is an issue that we have identified at least for physical disability groups that we have surveyed/interviewed. This also ensures a 'biopsychosocial approach' – in terms of CoS identifying personal factors but also factors in the social environment that are critical to RTW.	
**Work-life balance**	Increasing or maintaining productivity	We notice in people with Parkinson's they initially keep up productivity at work at cost of participation in other life roles. You partly measure this with 'need for recovery'… But would you consider including a measure about satisfaction with participation (e.g. USER-P- satisfaction scale?)	
**Additional perceptions affecting productivity**	Increasing or maintaining productivity	Are (self)-perceived stigma and perceived sef-efficacy at work covered in the concepts you describe?	
			A general problem I have with a lot of the current PROMS: they all assume the person is employed; some questions are not appropriate for people who are self-employed.
**Perceived competences**	Unclear		
**Impact of the disease on the career**	Unclear		
			The first question is that we probably should define the scope : individual level (e.g. effect of an intervention for people with cancer or depression) of collective level (effect of preventive measures on the rate of sick leave in a company),

			in order to priorize the COS
			The contentment for the job is mentioned but there is no COS for this
			Work ability index : effect of the disease or of aging ? We should include age in the analysis since the WAI may decrease with age, independently from the potential onset of diseases
**Predefined type of occupation**	Having employment	Sometimes people want to work in an occupation that meets their values more than money or time.	
**Predefined commute time**	Having employment	Work is a social activity for humans in the COMMUNITY. In some countries, it can take a long time to get to the workplace. Also, with regard to mental illness, people may be hesitant to work near their homes due to stigma. Therefore, matching commuting time seems to be important.	
**Predefined illness disclosure status**	Having employment	It would be important to make your own decisions about disclosure or nondisclosure for mental illness, HIV/AIDS, LGBT, and other conditions that are highly stigmatized.	
**Job satisfaction**	Having employment	Ultimately, job satisfaction appears to be most important, while measuring this outcome needs further discussion and scientific evidence.	
Lost working days <br>**(please note: I propose instead a slightly different measure: *working time of observed time ~ working days of days lived/observed*)**	Return to Employment	Basically, this measurement is sensitive to almost all core aspects of most other proposed measurements: time to RtW, sustained RtW and	

		risk of reoccurrence of sick leave:<br><br>It is sensitive to both sick leave duration of index sick leave *and* any reoccurrence of sick leave as well, and the length and timing of both of these events. This is contrary to *time to RtW (1.1.2), sick leave duration (1.2.5)* that are not sensitive to reoccurrence and *time to RtW… (1.1.4)* that is not sensitive to relapse beyond the specified threshold period (e.g. 4 weeks).	
Time to return to sustainable work, with high threshold (e.g. four consecutive months)	Return to Employment	Time to return to work (or any other positive event) is often not sensitive to reoccurrence – this problem can be handled by defining the threshold of sustainability rather high, like e.g. four months. The threshold of sustainability should be high enough, so that any reoccurrence after this threshold would not change the perception that the previous return to work was harmful, and retrospectively not recommended.	
**Skills for job procurement, externally assessed**	Initiating employment	Self-reported skill level is biased, why it should be externally assessed. Yet, job skills are most important for work participation, since work implies other stakeholders (costumers, employers etc.) should perceive work or services to have been delivered to pay money (which requires skills!)	
			I send you my proposals, but *I am not sure*, whether my

For peer review only

			choices/preferences are "missing outcomes" or just already mentioned outcomes. The reason for my doubts are thate the adjustments I propose might be so slight that the outcomes do not count as other and hence missing outcomes.
**Employment status operationalization**	Having employment	a) Labour market status during the last week of each year<br><br>OR<br><br>b) The main activity (i.e., labour market status) during the year<br><br>OR<br><br>c) The measure of labour market status, based on labour market status during each month.<br><br>###<br><br>a) and b) are strongly correlated.<br><br>We have used almost always a).	
**"Having lost employment" - early retirement**	Having employment	This seems to also be an important outcome	
**Time until employment since finishing school/vocational training**	Initiating employment	Would also be a more objective and internationally comparable outcome.	

For peer review only

For peer review only

This document contains 35 additional outcomes which were suggested by participants in the first round of Delphi voting. We consider whether the suggested outcomes match the criteria for outcomes suitable for COS for Work.

We would also like to draw your attention to the overarching definition of work participation used for COS for Work: *"work participation entails either performing work related activities or having the capacity to do a work related activity".* This definition is based on the definition of participation provided by the International Classification of Functioning, Disability and Health (ICF). The operationalization of this definition is not straight forward but the outcomes have to measure at the level of (not) performing a work related activity.

In addition, we use the criteria for outcomes suitable for the COS which were developed as part of the COS for Work framework.

For more reading on defining work participation, operationalization of outcomes and of the criteria, the framework can be accessed via **https://doi.org/10.21203/rs.3.rs-1342930/v1**

COS for Work outcomes criteria.

Criteria to consider	***Optional criteria***
Outcomes should:<br>1. Be sensitive to change<br>2. Be feasible to measure<br>3. Be applicable internationally<br>4. Be work participation specific<br>5. Capture multiple stakeholder perspectives<br>6. Be in alignment with the ICF model	*Outcomes should:*<br>1. Be used for cost-effectiveness studies<br>2. Be applicable across varying insurance schemes

Below we provide our argumentation for or against inclusion of the suggested outcomes in the next round of Delphi voting. If you disagree with our decision, there will be room to discuss this during our meeting on May 25.

For peer review only

Suggested outcome	Yes or No from COS team to include in next round	Reason
**Caregiver outcomes i.e. does caregiving affect work production**	No	This is an indirect variable/outcome. A contextual factor which may impact work but does not directly measure at either mental or physical level of "activity" by an individual.
**Worker satisfaction with job**	No	Job satisfaction was already included under outcome concept 2.5.1 "perceptions affecting productivity".
**Worker preceived stigma/bias from employer/society**	No	(Self) perceived stigma was already included under outcome concept 2.5.1 "perceptions affecting productivity".
**Provided with accomodation(s) for equitable work environment**	No	This is an indirect variable/outcome. A contextual factor which may impact work but does not directly measure at either mental or physical level of "activity" by an individual.
**Perceived social support - includes support from employer and access to appropriate work modifications)**	No	This is an indirect variable/outcome. A contextual factor which may impact work but does not directly measure at either mental or physical level of "activity" by an individual.
**Satisfaction of the worker with its career**	No	Job satisfaction was already included under outcome concept 2.5.1 "perceptions affecting productivity".
**Sustainable employment**	Yes	
**Work Ability Score (WAS)**	No	This is a measurement instrument which would measure the outcome 2.3.1 "work ability".
**Employment 3 - 5 years post injury/illness**	No	This would be a more specific definition of the outcome concept 3.1.4 (duration of employment).
**Accommodations requested and received**	No	This is an indirect variable/outcome. A contextual factor which may impact work but does not directly measure at either mental or physical level of "activity" by an individual.
**Quality of life**	No	Does not match criteria 4: COS for Work outcomes should be work participation specific.
**Health Status**	No	Does not match criteria 4: COS for Work outcomes should be work participation specific.

**Employer attention to occupational safety and health**	No	This is an indirect variable/outcome. A contextual factor which may impact work but does not directly measure at either mental or physical level of "activity" by an individual.
**Worker participation in job design**	No	This outcome is relevant in the context of workers participation decision making processes rather than evaluating a person's ability to work despite of a health problem.
**Worker participation in evaluation of injury control interventions**	No	This outcome is relevant in the context of workers participation decision making processes rather than evaluating a person's ability to work despite of a health problem.
**Adequacy of compensation system in replacing lost income after disability**	No	This is an indirect variable/outcome. A contextual factor which may impact work but does not directly measure at either mental or physical level of "activity" by an individual.
**Injury rate after return to work**	No	Does not match criteria 4: COS for Work outcomes should be work participation specific.
**Work Participation Score within 12 months following RTW; defined as a fraction with numbers of RTW weeks in the numerator and numbers of weeks receiving health related benefits in the denominator. K. Biering; N.H. Hjollund and T. Lund 2013**	No	This is at the level of measurement of RTW rather than on the level of outcome concepts.
**Average time from requested evaluation to determination**	No	Does not match criteria 4: COS for Work outcomes should be work participation specific.
**Possibilities to get a suitable job in the same organisation/group**	No	This would fall under the outcome concept 3.1.2 (having a pre-defined status of employment).
**Having support by your colleques - possibillitie that colleques take over task by your absence**	No	This is an indirect variable/outcome. A contextual factor which may impact work but does not directly measure at either mental or physical level of "activity" by an individual.
**Safe environment/culture in the organsiation by meaning workers**	No	This is an indirect variable/outcome. A contextual factor which may impact work but does not directly measure at either mental or physical level of "activity" by an individual.
**Number of HR coach /buddy's for returning/adjust to job in % total employees of organisations**	No	This is an indirect variable/outcome. A contextual factor which may impact work but does not directly measure at either mental or physical level of "activity" by an individual.
**Flexibility in hours and location to fulfill your tasks**	No	This is an indirect variable/outcome. A contextual factor which may impact

		work but does not directly measure at either mental or physical level of "activity" by an individual.
**Amount that is invested in hardware to maintain /keep workers (for instead invalid toilets rsi mouse)**	No	This is an indirect variable/outcome. A contextual factor which may impact work but does not directly measure at either mental or physical level of "activity" by an individual.
**Ability to visit a work related docter (arbocurative)**	No	This is an indirect variable/outcome. A contextual factor which may impact work but does not directly measure at either mental or physical level of "activity" by an individual.
**Work satisfaction vs normal workers**	No	Job satisfaction was already included under outcome concept 2.5.1 "perceptions affecting productivity".
**Level of education**	No	Baseline characteristic rather than an outcome measured at the start and end of an intervention to establish effectiveness.
**Sort of work (physical or mental capacity needed for the job)**	No	This is an indirect variable/outcome. A contextual factor which may impact work but does not directly measure at either mental or physical level of "activity" by an individual.
**Is the sick leave from a work related injury**	Yes/No	To be discussed
**Is there paid sick leave at the workplace**	No	This is an indirect variable/outcome. A contractual factor.
**Did workers see a company provided health care person or receive outside medical care**	No	This is an indirect variable/outcome. A contextual factor which may impact work but does not directly measure at either mental or physical level of "activity" by an individual.
**Did the person get any workers compensation for their injury**	No	This is an indirect variable/outcome. A contractual factor.
**If a work related injury; was the workplace changed to reduce chance of reinjury?**	No	This is an indirect variable/outcome. A contextual factor which may impact work but does not directly measure at either mental or physical level of "activity" by an individual.
**Hybrid work if position allows - in person and at home**	No	This is an indirect variable/outcome. A contextual factor which may impact work but does not directly measure at either mental or physical level of "activity" by an individual.

For peer review only

For peer review only

Results of the first round ratings combined for all stakeholders and stakeholder groups separately. Ratings are presented for each stage from most to least highly rated; a combination of mean score ($\bar{x}$) and the percentage of the stakeholders which rated an outcome with a score between 7-9. N.a.: not applicable. Stages of work participation: (1) initiating employment, (2) having employment, (3) increasing or maintaining productivity at work and (4) return to employment.

Stage	Outcome; clarifying text	Overall (n=55) $\bar{x}$; % 7-9	Researcher (n=35) $\bar{x}$; % 7-9	Employee (n=10) $\bar{x}$; % 7-9	Policy Maker (n=5) $\bar{x}$; % 7-9	Occupational Health Professional (n=4) $\bar{x}$; % 7-9	Employer (n=1) score
1	**Time to employment;** Time till getting a job	5,6; 44%	5,9; 51%	5,7; 50%	4,6; 0%	5; 25%	1 n.a.
1	**Self-efficacy for job procurement;** Self-efficacy for job procurement; Hope, optimism and self-efficacy for achieving vocational success; Social/intepersonal self-efficacy	5,6; 38%	5,4; 31%	6,1; 50%	6,2; 60%	5,8; 50%	1 n.a.
1	**Skills for job procurement;** Total work-related network, Engagement in work seeking activities, Job interview skills, Work-related benefit of vocational training	5,4; 38%	5; 29%	6,4; 60%	5,2; 40%	5,3; 50%	9 n.a.
2	**Employment - any type of employment including self-employment;** Any type of employment including self-employment	7,4; 75%	7,6; 80%	7,2; 70%	6,6; 60%	7; 75%	9 n.a.
2	**Employment - having lost**	6,5; 55%	6,4; 54%	7,5; 80%	5,4; 20%	7,3; 50%	1 n.a.

For peer review only - http://bmjopen.bmj.com/site/about/guidelines.xhtml

	**employment;** Having lost employment						
2	**Employment - predefined type of employment;** Having met a pre-defined status of employment; part/full time, competitive work in a mainstream setting, performing the same tasks as non-disabled workers	6,5; 51%	6,5; 51%	7,1; 60%	7,4; 60%	5; 25%	1 n.a.
2	**Work disability - Permanent and complete inability to engage in any work participation;** Permanent and complete inability to engage in any work participation	6,7; 34%	6,9; 63%	7,2; 80%	6; 40%	6,3; 50%	1 n.a.
2	**Work disability - Permanent partial disability to engage in work participation;** Permanent partial disability to engage in work participation	6,4; 31%	6,6; 51%	7,5; 90%	6; 40%	6; 50%	1 n.a.
2	**Employment - duration of employment;** Duration of employment	5,7; 40%	5,9; 40%	6; 50%	5,4; 20%	5; 50%	1 n.a.
2	**Employment - predefined type or amount of income;** Predefined type or amount of income	5,5; 33%	5,1; 23%	6,9; 70%	5,4; 20%	4.3; 25%	9 n.a.

For peer review only

3	**Work productivity loss (economic evaluation);** Overall loss of productivity at work (in terms of quality or output at work) resulting from presenteeism or absenteeism regardless of cause. Typically used for economic evaluations.	6,4; 58%	6,7; 63%	6,9; 70%	6; 40%	5; 25%	1 n.a.
3	**Work activity impairment;** Experienced functional impairment of performing work activities due to a health problem.	6,4; 51%	5,9; 40%	7,7; 80%	6; 40%	7,5; 75%	9 n.a.
3	**Work ability;** Current self-rated physical and mental work ability compared to lifetime best.	6,3; 53%	6,3; 46%	7,1; 90%	6,2; 40%	5,6; 50%	1 n.a.
3	**Presenteeism;** Not being productive while at work due to health problems. Typically used to evaluate the effect of health interventions for individuals.	5,9; 35%	5,8; 29%	6,7; 70%	4,6 ; 0%	5,3; 25%	9 n.a.
3	**Perceptions affecting productivity;** Beliefs in working capacity, motivation for work, vocational commitment, job coping, job satisfaction, need	5,5; 36%	5,1; 26%	7,2; 80%	5,2; 40%	4,3; 25%	9 n.a.

For peer review only

	for recovery, work-life balance, (self)-perceived stigma						
4	**Return to work - proportion of workers;** Proportion of workers that return to work after being absent because of illness	7,4; 76%	7,7; 80%	7; 70%	7,8; 80%	7,5; 75%	1 n.a.
4	**Return to work - time to return to work;** Time to return to work or duration of sickleave after being absent because of illness	7; 67%	7,3; 71%	6,8; 60%	6,8; 60%	7; 75%	1 n.a.
4	**Sustainable return to work – proportion of workers;** Proportion of workers that return to work and remain at work for a specified amount of time	6,7; 57%	6,3; 49%	7,6; 80%	7,2; 60%	6; 50%	9 n.a.
4	**Sustainable return to work – time until;** Time to return to work in workers absent because of illness and who do not relapse within a specified amount of time	6,3; 49%	6,1; 42%	6,9; 60%	6,6; 60%	5,8; 50%	9 n.a.
4	**Sick leave frequency;** Number of episodes of sick leave *per worker* expressed as percentage	5,8; 44%	5,4; 37%	6,9; 60%	5,6; 40%	5,5; 50%	9 n.a.
4	**Return to work - the worker's perceptions;** Self-efficacy for return	5,7; 38%	5; 20%	7,8; 80%	4,3; 20%	4,3; 25%	9 n.a.

	to work, return to work expectations or intention to return to work						
4	**Sick leave duration of all workers;** Working days or calendar days absent because of illness averaged over all workers	5,5; 38%	5,4; 37%	6,8; 50%	5; 20%	4; 25%	1 n.a.
4	**Sick leave duration - over all sick leave episodes;** Working days absent averaged over all sick leave episodes; can also be expressed as calendar days sick	5,4; 36%	5,6; 34%	6,8; 60%	4,8; 20%	3; 25%	1 n.a.
4	**Sickness absence among absent workers in an organization/group - lost working or sick days;** Working days or lost sick days as a percentage	5,3; 33%	5,3; 31%	6,1; 40%	6; 40%	4,3; 25%	1 n.a.
4	**Sickness absence among all workers;** Percentage of all working days of all workers in an organization during a time period e.g. 12 months; Sick days as percentage of all available days during a time period	5,3; 31%	5,3; 29%	6,8; 50%	6; 20%	4,3; 25%	1 n.a.

BMJ Open

Which outcomes should always be measured in intervention studies for improving work participation for people with a health problem? An international multi-stakeholder Delphi study to develop a core outcome set for work participation (COS for Work)

Journal:	*BMJ Open*
Manuscript ID	bmjopen-2022-069174.R1
Article Type:	Original research
Date Submitted by the Author:	20-Jan-2023
Complete List of Authors:	Ravinskaya, Margarita; Amsterdam UMC Locatie AMC, Department of Public and Occupational Health, Coronel Institute of Occupational Health, University of Amsterdam, Amsterdam Public Health research institute, Cochrane Work,<br>Verbeek, Jos ; University of Amsterdam, Amterdam UMC, Department of Public and Occupational Health, Coronel Institute of Occupational Health, University of Amsterdam, Amsterdam Public Health research institute, Cochrane Work<br>Langendam, Miranda; Amsterdam UMC Locatie AMC, Department Epidemiology and Data Science, University of Amsterdam, Amsterdam Public Health research institute<br>MADAN, IRA; GUYS AND ST THOMAS NHS FOUNDATION TRUST; King's College London Faculty of Life Sciences and Medicine<br>Verstappen, Suzanne; University of Manchester, Centre for Epidemiology Versus Arthritis, Faculty of Biology, Medicine and Health; NIHR Manchester Biomedical Research Centre<br>Kunz, Regina; University Hospital Basel, Research Unit EbIM, Evidence Based Insurance Medicine, Division of Clinical Epidemiology<br>Hulshof, C.; University of Amsterdam, Amsterdam UMC, Academic Medical Center, Department of Public and Occupational Health, Coronel Institute of Occupational Health, University of Amsterdam, Amsterdam Public Health research institute<br>Hoving, Jan; Amsterdam UMC Locatie AMC, Department of Public and Occupational Health, Coronel Institute of Occupational Health, University of Amsterdam, Amsterdam Public Health research institute, Cochrane Work
**Primary Subject Heading**:	Occupational and environmental medicine
Secondary Subject Heading:	Epidemiology, Health policy, Occupational and environmental medicine, Public health, Rehabilitation medicine
Keywords:	OCCUPATIONAL & INDUSTRIAL MEDICINE, PUBLIC HEALTH, REHABILITATION MEDICINE, STATISTICS & RESEARCH METHODS

I, the Submitting Author has the right to grant and does grant on behalf of all authors of the Work (as defined in the below author licence), an exclusive licence and/or a non-exclusive licence for contributions from authors who are: i) UK Crown employees; ii) where BMJ has agreed a CC-BY licence shall apply, and/or iii) in accordance with the terms applicable for US Federal Government officers or employees acting as part of their official duties; on a worldwide, perpetual, irrevocable, royalty-free basis to BMJ Publishing Group Ltd ("BMJ") its licensees and where the relevant Journal is co-owned by BMJ to the co-owners of the Journal, to publish the Work in this journal and any other BMJ products and to exploit all rights, as set out in our licence.

The Submitting Author accepts and understands that any supply made under these terms is made by BMJ to the Submitting Author unless you are acting as an employee on behalf of your employer or a postgraduate student of an affiliated institution which is paying any applicable article publishing charge ("APC") for Open Access articles. Where the Submitting Author wishes to make the Work available on an Open Access basis (and intends to pay the relevant APC), the terms of reuse of such Open Access shall be governed by a Creative Commons licence – details of these licences and which Creative Commons licence will apply to this Work are set out in our licence referred to above.

Other than as permitted in any relevant BMJ Author's Self Archiving Policies, I confirm this Work has not been accepted for publication elsewhere, is not being considered for publication elsewhere and does not duplicate material already published. I confirm all authors consent to publication of this Work and authorise the granting of this licence.

For peer review only

Which outcomes should always be measured in intervention studies for improving work participation for people with a health problem? An international multi-stakeholder Delphi study to develop a core outcome set for work participation (COS for Work)

Margarita Ravinskaya [a], Jos H. Verbeek [a], Miranda Langendam [c], Ira Madan[d,h], Suzanne M.M. Verstappen [f, g, h], Regina Kunz [e], Carel T.J. Hulshof [b], Jan L. Hoving [a]

[a] Cochrane Work, Amsterdam UMC, Academic Medical Center, University of Amsterdam, Coronel Institute of Occupational Health, Amsterdam Public Health research institute, Meibergdreef 9, 1105 AZ, Amsterdam, The Netherlands

[b] Amsterdam UMC, location Academic Medical Center, University of Amsterdam, Coronel Institute of Occupational Health, Amsterdam Public Health research institute, Meibergdreef 9, 1105 AZ, Amsterdam, The Netherlands

[c] Amsterdam UMC location Academic Medical Centre, Epidemiology and Data Science, Amsterdam, Amsterdam Public Health, Methodology, Meibergdreef 9, 1105 AZ, Amsterdam, The Netherlands

[d] Guy's and St Thomas' NHS Trust and Faculty of Life Sciences and Medicine, King's College London, United Kingdom

[e] Research Unit EbIM, Evidence-Based Insurance Medicine, University of Basel, Petersgraben 4, 4031 Basel, Switzerland

[f] Centre for Epidemiology Versus Arthritis, Faculty of Biology, Medicine and Health, The University of Manchester, Oxford Road, Manchester M13 9PL, United Kingdom

[g] NIHR Manchester Biomedical Research Centre, Manchester University NHS Foundation Trust, Manchester Academic Health Science Centre, 29 Grafton St, Manchester M13 9WU, United Kingdom

[h] MRC Versus Arthritis Centre for Musculoskeletal Health and Work, University of Southampton, University Rd, Highfield, Southampton SO17 1BJ, United Kingdom

Corresponding author. Margarita Ravinskaya. Cochrane Work, Amsterdam UMC, University of Amsterdam, Department of Public and Occupational Health, Coronel Institute of Occupational Health, Amsterdam Public Health research institute, Meibergdreef 9, 1105 AZ Amsterdam, The Netherlands. Tel.: +31 20 566 5337 E-mail address: m.ravinskaya@amsterdamumc.nl

Word count: 3838

For peer review only

ABSTRACT

Objective Synthesizing evidence of the effects of interventions to improve work participation among people with health problems is currently difficult due to heterogeneity in outcome measurements. A core outcome set for work participation is needed.

Study design and setting Following the Core Outcome Measures in Effectiveness Trials (COMET) methodology we used a five-step approach to reach international multi-stakeholder consensus on a core outcome set for work participation. Five sub-groups of stakeholders took part in two rounds of discussions and completed two Delphi voting rounds on 26 outcomes. A consensus of >= 80% determined core-outcomes and 50%-80% consensus was required for candidate-outcomes.

Results Fifty-eight stakeholders took part in the Delphi rounds. Core outcomes were: "any type of employment including self-employment", "proportion of workers that return to work after being absent because of illness" and "time to return to work". Ten candidate outcomes were proposed, among others: "sustainable employment", "work productivity", and "workers' perception of return to work".

Conclusion As a minimum, all studies evaluating the impact of interventions on work participation should include one employment outcome and two return to work outcomes if workers are on sick leave prior to the intervention.

Keywords work participation, employment, return to work, outcome measurements, core outcome set

Strengths and limitations of this study

- The core outcome set was developed in accordance with the Core Outcome Measures in Effectiveness Trials (COMET) guidelines
- A five-step approach was used to reach international multi-stakeholder consensus on a core outcome set for work participation.
- Five key stakeholder groups were involved in the consensus process: researchers, occupational health professionals, policy makers, employee/patient representatives and an employer
- Provided with comprehensive background information the stakeholders participated in two group discussions to discuss perspectives following a transparent Delphi procedure
- The majority of the stakeholders were researchers from Europe and North America

For peer review only

For peer review only - http://bmjopen.bmj.com/site/about/guidelines.xhtml

INTRODUCTION

Authors of systematic reviews (SRs) state that inconsistent reporting of work participation outcomes in clinical trials hampers evidence synthesis in the field of occupational health (OH).(1-3) Co-ordinating Editors of Cochrane review groups have indicated that the reliability and quality of SRs could be improved by using core outcome sets.(4) In 2019, the Coronel Institute of OH at AMC Amsterdam established an international research collaboration to develop a Core Outcome Set for Work Participation (COS for Work), based on the Core Outcome Measures in Effectiveness Trials (COMET) methodology.(5) Our aim was to develop a generic COS for Work to be used in intervention studies which expect to have an effect on work participation. As our COS is generic, and relevant to different diseases, it can be used for any type of intervention. For example, pharmaceutical studies for people with Rheumatoid Arthritis may not only improve clinical outcomes but indirectly also help participants participating in work. Such studies mostly measure work outcomes as a secondary outcome. Other types of interventions aim directly in helping people with work participation, such as return to work interventions for cancer survivors or workers with long COVID.

The first phase of our project involved a SR on the spectrum of work outcomes used to measure the effect of interventions in published randomized controlled trials (RCTs).(6) The SR showed extensive heterogeneity in work-participation outcome measurements and confirmed the need for a COS for Work. In addition, we saw that it was unclear why authors chose to measure outcomes in a certain way and that to create COS for Work we would need to have a framework for outcomes which would ensure meaningful, pragmatic and mutually exclusive categories in which outcomes could be placed. In the second phase, we created a framework to aid the selection of work participation outcomes.(7) The framework outlines four main stages of work participation: outcomes assessing whether a person is successful in acquiring or *initiating employment (stage 1)*, outcomes indicating whether a person is in employment, can retain employment or lost work within the duration of the study, i.e., *having employment (stage 2)*, outcomes that measure *increasing or maintaining productivity at work (stage 3)* when persons experience limitations or restrictions with working or have less output, and outcomes that assess *return to employment and sick leave from work (stage 4)* when people (temporarily) stop attending work and are on sick leave. Phase three involved a survey among reviewers and trial authors showing different reasons and preferences for choosing and using work outcomes. In particular, researchers choose outcomes because of their use in similar studies or their relevance for the health problem or type of intervention.(8)
In the fourth phase, we sought international consensus on COS for Work via a Delphi study. In the fifth and final phase, we will make recommendations for clinimetrically sound methods to measure the core outcomes selected during phase four.

The overall aim of the Delphi study is to define a comprehensive and minimal set of outcomes that is relevant and feasible for measuring the effectiveness of interventions that result in a change in work participation, based on consensus of an international, multi-stakeholder group.

METHODS

On-line multi-stakeholder discussions and Delphi voting

To develop methods, we followed the COMET guidelines (5), i.e. the Core Outcome Set—STAndards for Development (The COS-STAD).(9) For reporting we used the Core outcome set–STAndards for reporting (COS-STAR.(10) The protocol for this study was published on the COMET website prior to the commencement of the study (https://www.comet-initiative.org/studies/details/1195, online supplementary file 1), and includes an extensive explanation of the methodology. The five steps are summarized briefly below.

Step 1: preparation

We invited five groups of stakeholder representatives:

For peer review only

1. OH researchers – experienced researchers in OH field from varying disciplines, such as: health economy, occupational mental health, insurance medicine, workplace interventions, RtW interventions
2. OH professionals – physiotherapists, orthopedic surgeon, rehabilitation specialist
3. Workers/employees and patients – stakeholders representing patients and workers as members of patients federations, workers alliances and labor unions
4. Policy makers – stakeholders working for organizations creating OH policies such as the National Institute for Insurance against Accidents at Work, EU-OSHA
5. Employer – OH case manager

These five stakeholder groups were chosen as key stakeholders. Researchers will use the COS for Work and have experience with work participation outcomes measurement and the remaining stakeholder groups will use the results of what is researched in their professional or daily life.

Stakeholders were recruited from our phase two international survey (8) and our professional network. We anticipated that an effective group discussion with up to 30 participants would be feasible to allow diversity of opinion in the various subgroups. Our aim was to organize two group discussions of 30 participants, in the morning and late afternoon to account for the varying time zones. We sent out 90 invitations.

Stakeholders who agreed to participate received an information package that included [1] instructions for Delphi participation (online supplementary file 2), [2] a document containing the outcomes that would be included in the Delphi with worksheets to prepare for the first discussion (table 1, online supplementary file 3) and [3] a list of all participants in each discussion group. Item generation for the twenty four outcomes in the preliminary set (table 1) were based on the SR (6) and categorized under the four work participation stages as described in the framework (table 1).
Participants were invited to familiarize themselves with the material and add any outcomes, which they deemed missing from the preliminary list prior to the first discussion (online supplementary file 4). The research team evaluated whether these new outcomes fitted the COS for Work criteria (table 2) and presented the outcomes during the first discussion round.

For peer review only

Table 1. The preliminary set of outcomes that was presented to the stakeholders prior to the first discussion. *Item generation was based on the systematic review on how work participation outcomes are currently measured. Online supplementary file 3 contains the full overview with examples of how the outcomes could be measured.*

Stage of work participation — interventions and study populations for which outcomes are relevant within the stage	Outcome to be voted on during the Delphi
**Stage 1: Initiating employment:**<br><br>Interventions aiming to help unemployed people with distance to the job market due to a health problem get work	**1. Skills for job procurement.** Total work-related network, Engagement in work seeking activities, Job interview skills, Work-related benefit of vocational training
	**2. Self-efficacy**. Self-efficacy for job procurement, hope, optimism, and self-efficacy for achieving vocational success, social/interpersonal self-efficacy
**Stage 2: Having employment**<br><br>Relevant for any intervention and any type of health problem. Any type of health condition may impair work participation at the most elemental level, having work	**3. Employment. Having any type of employment including self-employment**
	**4. Employment. Having met a pre-defined status of employment**; part/full time, competitive work in a mainstream setting, performing the same tasks as non-disabled workers
	**5. Employment. Predefined type or amount of income**
	**6. Employment. Duration of employment**
	**7. Employment. Having lost employment**
	**8. Work disability.** Permanent and complete inability to engage in any work participation
	**9. Work disability.** Permanent partial disability to engage in work participation
**Stage 3: Increasing or maintaining productivity at work**<br><br>Interventions expected to help workers who are not on sick leave to maintain or increase productivity at work despite a health problem	**10. Work productivity loss (economic evaluation).** Overall loss of productivity at work (in terms of quality or output at work) resulting from presenteeism or absenteeism regardless of cause. Typically used for economic evaluations
	**11. Presenteeism.** Not being productive while at work due to health problems. Typically used to evaluate the effect of health interventions for individuals
	**12. Work ability.** Current self-rated physical and mental work ability compared to lifetime best.
	**13. Work activity impairment.** Experienced functional impairment of performing work activities due to a health problem
	**14. Perceptions affecting productivity.** Possible constructs: beliefs in working capacity, motivation for work, vocational commitment, job coping, contentment with work, need for recovery
**Stage 4: Return to employment and sick leave from work**<br><br>***Return to work:***<br>Interventions expected to help people who are on sick leave return to work	**15. Return to work - proportion of workers that return to work** after being absent because of illness
	**16. Return to work - time to return to work** or duration of sick leave after being absent because of illness
	**17. Sustainable return to work.** Proportion of workers that return to work and remain at work for a specified amount of time
	**18. Sustainable return to work.** Time to return to work in workers absent because of illness and who do not relapse within a specified amount of time
	**19. Return to work - the worker's perceptions.** Time it will take to return to work as perceived by the sick worker

For peer review only

Sick leave from work: (1) Interventions including people at risk of (frequent) sick leave due to a health problem, e.g. chronic conditions or recovery after an injury or procedure (2) Interventions aiming to prevent overall sickness absence among all workers	also described as self-efficacy for return to work, return to work expectations or intention to return to work
	**20. Sickness absence among all workers** in an organization/ group expressed as lost working days or lost sick days
	**21. Sick leave duration.** Working days absent because of illness averaged over all workers
	**22. Sick leave frequency**. Number of episodes of sick leave *per worker* expressed as percentage
	**23. Sickness absence among absent workers** in an organization/group expressed as lost working days or lost sick days
	**24. Sick leave duration.** Working days absent because of illness averaged over all workers

Table 2: Criteria for COS outcomes which the stakeholders were asked to consider when deciding for or against inclusion of outcomes in COS for Work.

Criteria to consider	*Optional criteria*
Outcomes should:<br>1. **Be sensitive to change**<br>2. **Be feasible to measure**<br>3. **Be applicable internationally**<br>4. **Be work participation specific**<br>5. **Capture multiple stakeholder perspectives**<br>6. **Be in alignment with the ICF model**	*Outcomes should if possible/applicable:*<br>1. Be used for cost-effectiveness studies<br>2. Be applicable across varying insurance schemes

Step 2: First online consensus stakeholder meeting

In the first online meeting, we presented the steps and research findings of the COS development including the four stages of work participation (see Table 1) and criteria for COS outcomes (table 2). Further, we elaborated on the various stakeholder perspectives and its effect on outcome prioritization, and discussed the proposed additional outcomes. In breakout rooms, groups of stakeholders created and discussed "absolutely in" and "absolutely out" outcomes for each stage. The results were discussed in the plenary session.

Step 3: First round of Delphi voting

COMET online Delphi Manager software participants voted on a total of twenty-five outcomes (outcomes from table 1 plus one outcome suggested by a stakeholder) accompanied by clarifying text on how it could be measured. With a personal login, the DelphiManager software allowed each outcome to be anonymously ranked on a Likert Scale from 1-9: from not important (0 points) to critical (9 points), including an "unable to score" option. Participants were asked to provide rationale for their ratings and additional outcomes could be suggested. A single voting reminder was sent to participants two days before the closing of the first round.

Step 4: Second online stakeholder meeting

The results of the first online Delphi voting round and a collated list of arguments on why to include or exclude an outcomes were first distributed via e-mail and then discussed in the second online stakeholder meeting. We also presented our analysis of suggested additional outcomes (online supplementary file 5). Based on the a-priori defined consensus definition (see 'data analysis') and input from the stakeholder meeting, the authors then decided which outcomes would be dropped, included, reformulated or added to the second Delphi round.

Step 5: Second Delphi voting round

For peer review only

The same online Delphi procedure allowed each participant to individually rate the outcomes that remained from the first Delphi round. As before, reminders were sent to non-completers.

Patient and Public involvement

Consultation of patients' is seen as a crucial aspect in the development of COS.(5) We have consulted patient representatives, who were also representing the 'employee' perspective, in the discussion rounds and the selection of core outcomes for COS for Work. The patient representatives were members of, amongst others: The Netherlands Patients Federation, Canadian Injured Workers Alliance and labor unions. In addition, we also involved policy makers active in the field of occupational health. The results were disseminated to all participants via e-mail.

Data analysis

After the first and second stakeholder meetings we held a meeting with the steering group to discuss how we could use and present the stakeholder input for the Delphi voting to inform all Delphi participants. The results from the Delphi rounds were extracted from the Delphi Manager software into a CSV format and analyzed in SPSS using descriptive statistics.

For each round, we calculated the distribution of scores for all outcomes as well as the percentage of stakeholder scoring between the 7-9. As per protocol, outcomes scored between 7-9 by at least 80% of all stakeholders were deemed as core – showing as "*definite* core outcome IN" on the spreadsheet distributed amongst stakeholders. Outcomes receiving a mean score between 7 -9, from 50% - 80% of the participants in the first Delphi round were "candidate outcomes" to be discussed with the stakeholders and re-rated in the second Delphi round – labeled as "*possible* core outcome IN". Outcomes which received a score between 7-9 from less than 50% of the participants were dropped after the first round. For outcomes that were not considered definite core outcome but for which there was strong disagreement between stakeholder groups in the first round, the steering group still considered them for being discussed and rated in the second round. It was possible to upgrade or downgrade the ranking of an outcome for inclusion or exclusion in the second Delphi round based on argumentation of stakeholders. The final core outcome set is based on the rankings of the second round.

RESULTS

Respondents

Initially we approached 90 potential stakeholders and 59 stakeholders agreed to participate (not everyone from this group registered for the Delphi). It was difficult to recruit employer and employee representatives; we had no replies from employee representatives and one employer. After the first discussion round, participants helped us to recruit 11 employee representatives through their professional network. In total 70 stakeholders agreed to participate. Twelve participants took part in either one or two of discussions but did not register for the Delphi Manager software, some of whom e-mailed that they did not manage to due to time constraints (See Table 3 for participant characteristics).

Table 3. Characteristics of survey respondents for two Delphi rounds (n; %). In total 58 participants registered as participants in the Delphi Manager. Each participant belonged to one stakeholder group. In every round three participants did not complete the voting.

	Delphi round 1 Number of participants	Delphi round 2 Number of participants
Stakeholders	58; 100%	58; 100 %
• **Researcher**	35; 60%	37; 63%
• **Employee**	10; 17%	10; 17%
• **Occupational Health professional**	4; 7%	4; 7%

• **Employer**	1; 2%	1; 2%
• **Policy maker**	5; 9%	3; 5%
• **Non-completers**	3; 5%	3; 5%;
**Location of residence**		
• **Europe**	31; 53%	30; 52%
• **North America**	17; 31%	17; 29%
• **Australia**	3; 5%	4; 7%
• **Asia**	3; 5%	3; 5%
• **South America**	1; 2%	1; 2%

First online consensus stakeholder discussion

We presented all Delphi voting information (ratings and arguments) to the stakeholders as outlined in section 2.1. The stakeholders discussed their preferences for and against the outcomes in four breakout rooms. They then presented their arguments in a plenary session that included discussion on topics such as:

- when work can be considered as sustainable and healthy and the challenges of measuring such concepts as part of this COS,
- which types of outcomes would be applicable and feasible to measure in an international context,
- why COS outcomes should have a positive focus (such as work-ability) rather than negative (such as sick leave).

We received fifteen outcomes (online supplementary file 4) which stakeholders found missing from the preliminary set (table 1). We did not include the outcomes which did not match with the COS criteria (table 2), outcomes which were already included but phrased slightly different, and outcomes which were a specification on the level of measurement. Two outcomes were added to the list for the first round of Delphi voting: (1) "(self)-perceived stigma" to the *perceptions affecting productivity* outcome concept and (2) "time to employment" to the *initiating employment* stage.

First round of Delphi voting and second online stakeholder discussion

The ratings for the first round of Delphi voting are presented in the online supplementary file 6. None of the outcomes within the four stages received our cut-point for "definite core outcome IN" (>80% score of 7-9 points).

<u>Stage 1: Initiating employment</u>

The highest-ranking outcome was "time to employment" with a mean score of 5.6 and 44% of stakeholders giving it a score between 7 - 9. To not entirely exclude this stage from the second round, the steering group included the newly proposed outcome "any type of employment including self-employment" for the second Delphi round.

<u>Stage 2: Having employment</u>

"Any type of employment" was the highest-ranking outcome with a mean score of 7.4 and 75% of the stakeholders scoring it between 7-9. Stakeholders indicated that this outcome was feasible to measure internationally irrespective of social insurance schemes. In addition, this outcome was also seen as an indicator of the degree of work participation when sickness absence is not measured, and people lose work because of a health problem - relevant for countries where sickness benefits are limited. An additional outcome was proposed by a stakeholder, discussed, and agreed for inclusion in the second round: "sustainable employment" (online supplementary file 5). The employee representatives advocated to define "sustainable" in terms of quality of the working life, such as having decent work and healthy work.

<u>Stage 3: Increasing or maintaining productivity at work</u>

For peer review only

"Work productivity loss", "Work activity impairment" and "Work ability" received "possible core outcome IN" rankings, and during the discussion, there were no objections, biddings to downgrade any of them or exclude them from the second round. "Perceptions affecting productivity" was ranked to be left out from the second round. However, we did not exclude this outcome as the outcome concept included measures such as work-life balance and job-satisfaction, which were brought forward as highly important by several stakeholders during the discussion. Therefore, it was upgraded for inclusion during the discussion.

<u>Stage 4: Return to employment</u>

The steering group discussed the disagreement on "Return to work – workers' perceptions' which were voted out by all stakeholder groups combined. The main argument against including it was whether the outcome actually measured work participation and whether it would be relevant to measure at the beginning and end of all types of interventions. However, this outcome was ranked as "definite core outcome IN" by the employee representatives and some stakeholders from other groups were keen on including it in the second round. Therefore, it was not excluded All versions of sickness absence outcomes received low ratings. Feedback from stakeholders suggested that this may be due to varying insurance schemes, how it is registered, lack of compensation of sick leave in many countries and in some cases punitive measures for being on registered sick leave. However, as there are interventions for which it is relevant to measure accumulated absence over time due to a health problem, and return to work may not be a relevant measure, the steering group decided to keep one absence outcome in the second round. We created a new outcome for the second round, with the aim of replacing sick leave terminology: "Duration of absence from work due to a health problem over a period of time". In addition, we added one outcome "having lost employment" to this stage, as an indicator of workers who do not return to work at all after a period of health-related absence.

3.4 *Second Delphi round – final vote on core outcomes*

Results of the second round are presented in table 4 and table 5 contains the COS for Work.

<u>Stage 1: Initiating employment</u>

"Having any type of employment including self-employment" as an outcome for interventions aiming to help unemployed people get work did not meet the consensus criteria to be included in the COS for Work.

<u>Stage 2: Having employment</u>

"Any type of employment including self-employment" received 87% consensus and was therefore included in the COS for Work

<u>Stage 3: Increasing or maintaining productivity at work</u>

None of the outcomes in this stage received enough consensus to be included in the COS for Work. However, "work productivity loss", "work ability" and "work activity impairment" received similar ratings to round one indicating they are seen as important but not critical for the COS for Work.

<u>Stage 4: Return to employment</u>

Two outcomes received overall consensus for inclusion: (1) "Return to work – proportion" and (2) "Time to return to work". "Proportion of workers with sustainable return to work" was voted as important but not critical by all stakeholders. "Workers' perception on return to work" received 90% agreement within the employee representatives group for inclusion but very low scores by other stakeholder groups. The "duration of absence from work due to health problem over a period of time" also received low scores for inclusion (38% score between 7-9), making it a higher rating than all the sick leave outcomes from the first round – but nonetheless excluding any absenteeism outcome from consideration for the COS for Work.

For peer review only

Table 4. Results of the second-round ratings presented for all stakeholders combined (overall) and for each stakeholder group separately. Ratings are presented for the four work participation stages. The outcomes from the Delphi rounds are presented within each stage from most to least highly rated. Only outcomes which received a mean score of 7-9 for 50% - 80% of the participants in the first Delphi round are included. We present the mean score ($\bar{x}$) given to each outcome per group and the percentage of the stakeholders within each group who rated an outcome with a score between 7-9. Further description of analysis can be found in section 'data analysis'. N.a.: not applicable.

Outcome<br><br>Clarifying text for new outcomes added to the second round (table 1 contains all initial outcomes)	Overall (n=55)<br>$\bar{x}$; % score 7-9	Researcher (n=37)<br>$\bar{x}$; % score 7-9	Employee (n=10)<br>$\bar{x}$; % score 7-9	Occupational Health Professional (n=4)<br>$\bar{x}$; % score 7-9	Policy Maker (n=3)<br>$\bar{x}$; % score 7-9	Employer (n=1)<br>score
**Stage 1: Initiating employment**						
Any type of employment including self-employment<br>(Outcome replaces all previous outcomes for stage 1. Also included in stage 2.)	6,2; 55%	6,6; 65%	6; 40%	5,3; 25%	6; 33%	1<br>n.a.
**Stage 2: Having employment**						
Employment - any type of employment including self-employment	7,9; 87%	8; 89%	7,4; 80%	7; 7,5%	8,7; 100%	9<br>n.a.
Sustainable employment<br>(New outcome based on suggestion from Delphi round 1 and the discussion: Any type of employment including self-employment AND one or more indicators of quality of working life (such as work life balance, healthy work, optimal work, work accommodations, decent work)).	7,2; 66%	6,7; 60%	8,6; 100%	7,8; 75%	5; 0%	9<br>n.a.
Work disability - self reported inability to engage in any work participation (Rephrased outcome based on the discussion).	6,9; 64%	7; 65%	7,4; 80%	7,8; 75%	4,3; 0%	1<br>n.a.
Employment - having lost employment	6,5; 56%	6,7; 60%	7,6; 80%	6; 25%	4,3; 0%	1<br>n.a.
**Stage 3: Increasing or maintaining employment**						
Work productivity loss (economic evaluation)	6,4; 62%	6,6; 70%	6,5; 50%	5; 25%	6,7; 67%	1<br>n.a.
Work ability	6,5; 60%	6,6; 60%	7,3; 90%	5,5; 25%	6,7; 67%	1<br>n.a.
Work activity impairment	6; 51%	5,5; 41%	7,5; 80%	6,8; 50%	6; 67%	9<br>n.a.
Perceptions affecting productivity	5,2; 35%	4,7; 24%	6,8; 70%	5; 50%	4,3; 0%	9<br>n.a.
**Stage 4: return to employment**						

For peer review only - http://bmjopen.bmj.com/site/about/guidelines.xhtml

Return to employment						
Return to work - proportion of workers	8; 91%	8; 92%	7,6; 90%	8,5; 100%	7,7; 67%	9<br>n.a.
Time to return to work	7,8; 80%	8; 84%	7,6; 80%	7,8; 75%	6; 33%	9<br>n.a.
Sustainable return to work – proportion of workers	7,2; 62%	7,2; 62%	7,7; 70%	7,8; 75%	5,3; 33%	9<br>n.a.
Duration of absence from work due to health problem over a period of time. (**This outcomes replaces previous sickness absence outcomes based on the suggestions from round 1 and the discussion**)	6; 38%	6; 40%	6,7; 40%	5,5; 25%	6,7; 33%	1<br>n.a.
Return to work - the worker's perceptions.	5,4; 38%	5,1; 24%	7,4; 90%	3,5; 25%	4,3; 33%	9<br>n.a.

Table 5: COS for Work. Outcomes which were selected by the panel to always be measured in studies measuring an effect on work participation.

**Stage of work participation**<br>**Interventions and study populations for which the outcomes are relevant within the stage**	**COS for Work**<br>**Outcomes which should always be measured in intervention studies relevant for the stage of work participation**
**Having employment**<br><br>Relevant for any intervention and any type of health problem. Any type of health condition may impair work participation at the most elemental level, having work	1. Having any type of employment including self-employment
**Return to work**<br><br>Interventions expected to help people who are absent from work due to a health problem return to work	1. Proportion of workers that return to work after being absent because of illness<br>2. Time to return to work in workers absent because of illness

DISCUSSION

Summary of main findings

Our international Delphi panel achieved consensus on three work participation outcomes for inclusion in COS for Work. As a minimum, all intervention studies addressing work participation should include *"any type of employment including self-employment"* as an outcome. Intervention studies which include participants who are absent from work due to a health problem should include the outcomes *"proportion of workers that return to work after being absent because of illness"* and *"time to return to work"*.

Strengths

We followed recommendations by the COMET initiative (5) to design the Delphi study, as well as the Core Outcome Set—STAndards for Development: The COS-STAD (9) and Core outcome set–STAndards for reporting: the COS-STAR.(10) For example, based on these standards we described the consensus definition *a priori* in a publicly available protocol. In addition, we used clearly communicated and specifically designed criteria for deciding which additional outcomes suggested by stakeholders would be suitable for COS for Work. Our systematic review provided us with a comprehensive overview of how work participation is measured and reported internationally. We were able to cluster all the outcome concepts we found in the literature into twenty-four outcomes. Working with a relatively low number of outcomes is a facilitating factor for having focused discussions with stakeholders and may have contributed to the low attrition rate. (11, 12)

The Delphi Manager software was designed by the COMET initiative to conduct Delphi studies. We had a group of international stakeholders representing a wide range of expertise relevant for research in the field of occupational health. The online discussions provided valuable exchange of perspectives between the stakeholders. For instance, through sharing the information on the various social security systems it became clear why some outcomes would be less feasible to measure internationally.

Limitations

A limitation of the study is that we do not have core outcomes for all four work-participation stages. However, for some outcomes that did not meet the inclusion criterion, the consensus was still high and they are good candidates to be considered for measurement in studies (see Table 6).

Another limitation of this study is that we had difficultly recruiting employee and employer representatives. The employee group did not join the first online discussion. As our panel consisted mostly of researchers it is likely that views of the other four stakeholder groups were underrepresented. Nonetheless, as COS for Work is developed to be used by researchers we believe that it addresses issues researchers face at an elemental level. As we presented the work participation stages with relevant outcomes in order of most to least commonly measured (6) the “initiating employment” stage might have received less, and possibly not enough, attention.

Most participants were from Europe and North America. This might have influenced which outcomes are seen as most relevant for studies conducted on these continents and the results might be less applicable to low-and middle-income countries.

Results compared to previous findings

In terms of defining core outcomes for work participation most research to date has been done by the Outcome Measures in Rheumatology (OMERACT) initiative.(13) Their main focus is on worker productivity loss including outcomes such as absenteeism, presenteeism and costs, and focus on people with rheumatic diseases and musculoskeletal diseases.(14, 15) We consider that COS for Work is a suitable addition to include in studies which measure such productivity outcomes.

Sustainable employment was proposed as a potential COS outcome by a stakeholder during the first Delphi round. While we did not find this outcome as a commonly measured work outcome in the literature (6) it appears to be a highly important outcome from the employee perspective. Sustainable return to work is commonly measured in terms of having the ability to work for an extended period. However, employee representatives and some researchers understand sustainable employment in terms of quality of work, having decent work and a good work-life balance. Defining sustainability in terms of healthy work is in line with results of a study done among employees on how they perceive sustainable work.(16)

Implications for practice and research

In table 6 we present the candidate outcomes that did not meet the cut-off but were also highly ranked by stakeholders. These findings are not reported in the results section as the aim of the study was to determine core outcomes for work participation. However, there were several outcomes for which there was strong

For peer review only

disagreement between the stakeholder groups. The reasons are discussed in the results section; the summary of the stakeholder discussions and ratings of the Delphi voting rounds. It is possible that the candidate outcomes did not reach the consensus criterion due to the background of the researchers (most researchers do not investigate outcomes relevant to help people with initiating employment). In addition, group discussion generally supported an outcome such as ‘sustainable employment’, but lack of consensus on the exact definition may have influenced low scores in the second Delphi voting round. Further research is needed to investigate which of these outcomes should be prioritized under which circumstances.

Table 6: Candidate outcomes. *These outcomes were also high ranking (by at least one group) and need further investigation in which context they could be (additional) COS for Work outcomes.*

Stage of work participation — interventions and study populations for which outcomes are relevant within the stage	Potential (additional) future COS outcomes which did not meet the cut-point for COS for Work inclusion
**Stage 1: Initiating employment**<br><br>Interventions aiming to help unemployed people with distance to the job market due to a health problem get work	• Having any type of employment including self-employment
**Stage 2: Having employment**<br><br>Relevant for any intervention and any type of health problem. Any type of health condition may impair work participation at the most elemental level, having work	• Sustainable employment<br>• Work disability - self reported inability to engage in any work participation<br>• Employment – having lost employment
**Stage 3: Increasing or maintaining productivity at work**<br><br>Interventions expected to help workers who are not on sick leave to maintain or increase productivity at work despite a health problem	• Work productivity loss (economic)<br>• Work ability<br>• Work activity impairment
**Stage 4: Return to work**<br><br>Interventions expected to help people who are on sick leave return to work	• Sustainable return to work – proportion of workers<br>• Workers’ perceptions on return to work<br>• Having lost employment

In our next study we will define and evaluate *how* to best measure ‘having any employment’ and ‘return to work’ in the COS for Work. Use of COS for Work by researchers will greatly help with evidence synthesis for researchers thereby helping decision making for practice and policy.

For peer review only - http://bmjopen.bmj.com/site/about/guidelines.xhtml

Acknowledgements

We would like to thank all the stakeholders who participated in this study for contributing to the results for COS for Work, the investment of time and the lively discussions. The following stakeholders have agreed to be publicly acknowledged as participants:

Aasdahl, Lene (NO); Anema, Johannes (NL); Berkowitz, Debbie (US); Bethge, Matthias (DE); Bühler, Jonas (CH); Bültmann, Ute (NL); Böckerman, Petri (FI); Coons, Trevor (US); Curti, Stefania (IT); Crosse, Caroline (AU); de Boer, Angela (NL); Dorstyn, Diana (AU); Foster, Nadine (AU); Friberg, Emilie (SE); Gehanno, Jean Francois (FR); Godderis, Lode (BE); Goodson, Nicola (UK); Graveline, Christine (CA); Gross, Douglas (CA); Hara, Karen Walseth (NO); Hannu, Timo (FI); Hoff, Andreas (DK); Hensing, Gunnel (SE); Hegewald, Janice (DE); Hoorntje, Alexander (NL); Janssen, Svenja (NL); Lacaille, Diane (CA); Lam, Raymond (CA); Landsbergis, Paul (US); Luiza Comper, Maria (BR); Lytsy, Per (SE); Macfarlane, Gary (UK); Mantis, Steve (CA); Merry, Kohle (CA); Meyers, Alysha (US); Neupane, Subas (FI); Nygård, Clas-Hakan (FI); Oyeflaten, Irene Larsen (NO); Pingle, Shyam (IN); Prior, Yeliz (UK); Schaafsma, Frederieke (NL); Samant, Yogindra (NO); Shaw, William (US); Snyder, Alexis (US); Steenstra, Ivan (CA); Sturkenboom, Ingrid (NL); Suijkerbuijk, Yvonne (NL); Tsutsumi, Akizumi (JP); Urquhart, Donna (AU); van Ee, Ilse (NL); van Zaanen, Yvonne (NL); Yamaguchi, Sosei (JP); Zhang, Wei (CA); Walker Bone, Karen (UK)

Funding

This research received no specific grant from any funding agency in the public, commercial or not-for-profit sectors. SV is supported by Versus Arthritis (grant numbers 20385, 20380) and the NIHR Manchester Biomedical Research Centre.

Contributors

MR, JH, JV, RK, CH, ML, IM, SV contributed to the conception, the study design and the execution of the Delphi. MR and JH tested the Delphi manager software prior to the first Delphi round. MR and JV analyzed the data. MR and JH assessed data quality. MR drafted the first version of the manuscript and the minor revisions, and JH, JV, RK, CH, ML, IM, SV contributed to the, the review and the editing process.

Competing interests

None declared.

Patient and public involvement

Patients and/or the public were involved in the design and conduct plans of this research as described in the Methods section.

Patient consent for publication

Not applicable.

Data sharing statement

All relevant data has been included in the article. Data are available upon request.

Ethics Approval

The research was conducted in accordance with the Declaration of Helsinki. The research proposal was submitted and approved by the Medical Ethical Committee of the Amsterdam UMC, Academic Medical Centre, Amsterdam, which judged that a comprehensive evaluation was not required since this study was not subject to the Netherlands' Medical Research Involving Human Subjects Act (reference: W22_129 # 22.171).

For peer review only

Reference list

1. Hoving JL, Lacaille D, Urquhart DM, et al. Non-pharmacological interventions for preventing job loss in workers with inflammatory arthritis. Cochrane Database of Systematic Reviews. 2014(11).
2. de Boer AG, Taskila TK, Tamminga SJ, et al. Interventions to enhance return-to-work for cancer patients. Cochrane database of systematic reviews. 2015(9).
3. Nieuwenhuijsen K, Faber B, Verbeek JH, et al. Interventions to improve return to work in depressed people. Cochrane Database of Systematic Reviews. 2014(12).
4. Kirkham JJ, Gargon E, Clarke M, et al. Can a core outcome set improve the quality of systematic reviews?–a survey of the Co-ordinating Editors of Cochrane Review Groups. *Trials*. 2013;14(1):21.
5. Williamson PR, Altman DG, Bagley H, et al. The COMET handbook: version 1.0. Trials. 2017;18(3):1-50.
6. Ravinskaya M, Verbeek JH, Langendam M, et al. Extensive variability of work participation outcomes measured in randomized controlled trials: a systematic review. *J Clin Epidemiol*. 2022;142:60-99.
7. Ravinskaya M, Verbeek JH, Langendam MW, et al. A general framework for selecting work participation outcomes in intervention studies among persons with health problems: a concept paper. *BMC Public Health*. 2022;22(1):1-15
8. Ravinskaya M, Verbeek JH, Langendam MW, et al. Preferred Methods of Measuring Work Participation: An International Survey Among Trialists and Cochrane Systematic Reviewers. *J Occup Rehabil*. 2022:1-
9. Kirkham JJ, Davis K, Altman DG, et al. Core outcome Set-STAndards for development: the COS-STAD recommendations. *PLoS Med.* 2017;14(11):e1002447.
10. Kirkham JJ, Gorst S, Altman DG, et al. Core outcome set–STAndards for reporting: the COS-STAR statement. *PLoS Med*. 2016;13(10):e1002148.
11. Williamson PR, Altman DG, Bagley H, et al. The COMET handbook: version 1.0. *Trials*. 2017;18(3):280.
12. Gargon E, Crew R, Burnside G, et al. Higher number of items associated with significantly lower response rates in COS Delphi surveys. *J Clin Epidemiol*. 2019;108:110-20.
13. Verstappen SM, Lacaille D, Boonen A, et al. Considerations for evaluating and recommending worker productivity outcome measures: an update from the OMERACT worker productivity group. *J Rheumatol.* 2019;46(10):1401-5.
14. Escorpizo R, Bombardier C, Boonen A, et al. Worker productivity outcome measures in arthritis. *J Rheumatol*. 2007;34(6):1372-80.
15. Gladman DD, Mease PJ, Healy P, et al. Outcome measures in psoriatic arthritis. *J Rheumatol*. 2007;34(5):1159-66.
16. Rooijackers B, de Rijk A, Horstman K, et al. A social ecology of sustainable employability: a qualitative study among Dutch employeesInge Houkes. *Eur J Public Health.* 2015;25(suppl_3).

For peer review only

PROTOCOL

REACHING CONSENSUS ON A CORE OUTCOME SET FOR WORK PARTICIPATION. A DELPHI STUDY.

Margarita Ravinskaya, Jos H. Verbeek, Miranda W. Langendam, Ira Madan, Suzanne M.M. Verstappen, Regina Kunz, Carel T.J. Hulshof, Jan L. Hoving

Date: 21 March 2022

INTRODUCTION

Authors of systematic reviews (SRs) state that inconsistent work participation outcome reporting in clinical trials hampers evidence synthesis in the field of occupational health (1-3). Co-ordinating Editors of Cochrane review groups indicate that reliability and quality of SRs could be improved by use of core outcome sets (4). In 2019, the Coronel Institute of Occupational Health at AMC Amsterdam established an international research collaboration to launch the development of a Core Outcome Set for Work Participation (COS for Work), based on the Core Outcome Measures in Effectiveness (COMET) Trials approach (COMET Initiative | Development of a Core Outcome Set for Work Participation (COS-WP) (comet-initiative.org)

The first phase of our research project involved a systematic review on the spectrum of work outcomes used to measure the effect of interventions in published trials (5). This review showed extensive heterogeneity in work participation outcome measurements and a need for a COS for Work. In the second phase we completed a survey among systematic reviewers and trial authors to gain insight in the reasons and preferences for choosing and using work outcomes (6). In addition, we created a framework to aid the selection of outcomes on of work participation (submitted). The framework outlines four main stages of work participation which can be used to group unique outcomes relevant for each stage. The stages are: (a) initiating employment, (b) having employment, (c) increasing or maintaining productivity at work and (d) return to employment.

In the current third phase we seek consensus on a comprehensive core set of work outcomes which can be used in all intervention studies that are expected to impact work participation directly or indirectly. In the fourth and final phase we will establish a list of validated outcome instruments/ measurable definitions for each of the core work outcomes.

The overall aim of this Delphi study is to define a minimal set of outcomes that are relevant, valid and feasible for measuring the effectiveness of interventions to achieve higher work participation, based on the consensus of an international, interdisciplinary and interprofessional stakeholder group.

For peer review only - http://bmjopen.bmj.com/site/about/guidelines.xhtml

METHODS

<u>Consensus process</u>

Steps of the consensus process

The consensus process will include five steps:

1. Preparation
2. First online consensus participant discussion (20 April 2022)
3. First round of Delphi voting (11-14 May 2022)
4. Second online discussion about the results of the first Delphi round (25 May 2022)
5. Second Delphi voting round (30 May 2022)

<u>1. Preparation</u>

The objective of this step: prepare the participants for the first online discussion.

Two groups participate in this study:

1. The international COS steering group with researchers from four countries:

   Jan Hoving (NL) , Margarita Ravinskaya (NL), Carel Hulshof (NL), Suzanne Verstappen (UK), Ira Madan (UK), Regina Kunz (CH), Miranda Langendam (NL), Jos Verbeek (FI)

2. Study participants.

   Five types of international stakeholders will be recruited for the consensus process:

   1. Researchers in occupational health with experience in RCT/SR and / or cost-effectiveness studies
   2. Health professionals in occupational health and insurance medicine
   3. Representatives of workers (e.g. union members)
   4. Representatives of employers (e.g. members of business federations)
   5. Representatives of policy makers (e.g. government representatives, members of EU OSHA or the World Health Organization)

Participants may also fulfill multiple roles (for example be a researcher and health professional) and share their knowledge and experience as input during the discussion rounds. During the Delphi rounds, however, the participants must choose one stakeholder group that they wish to represent. We will assign the stakeholder roles to every participant based on their background and confirm prior to the first discussion round if they agree. The majority of the participants will be researchers because their experience with measuring work participation will be helpful in making informed decisions. We arranged this composition of stakeholders to provide a diversity of views from the health, societal, worker and employer perspectives. There are no set guidelines on the number of participants for the consensus on core outcome sets. A study by Signha et al. that the number of participants to range from 13 to 222 (7). We will recruit about 100 participants with the aim to have 50 participants take part in the consensus.

For peer review only - http://bmjopen.bmj.com/site/about/guidelines.xhtml

We will recruit potential via the following channels:

1. Invite researchers who have responded to our survey (6) and indicated that we could contact them in the future
2. Invite researchers and other stakeholders through our international network

We will identify the potential participants based on their professional background and the geographical location. Participants living in Europe, North and South America, Asia, Australia and Africa will be invited. An e-mail will be sent out specifying the aims of the project, the required input and dates of the plenary discussions and the Delphi rounds.

Using the results from the systematic review, the survey and the new framework, the steering group will write a concise background document for participants attending the two rounds of the consensus process. This includes information on the COS for Work framework, the various work participation outcomes and definitions used in the literature and our proposed definitions for the different outcomes which will be used in the Delphi voting rounds. The participants are asked to reserve approximately two hours to prepare for the consensus process by reading/familiarizing themselves with the background document. In addition they are asked to provide written feedback about any outcome which is not part of our preliminary set but they would find potentially core with their reasoning behind it.

2. First online consensus participant discussion

The objectives of the online discussion are as follows:

1. Present the COS for Work framework with the four stages of work participation: (1) initiating employment, (2) having employment, (3) increasing or maintaining productivity at work, and (4) return to employment
2. Make explicit that within each stage of work participation there are various outcome concepts and we are seeking consensus on core outcomes belonging to each category.
3. Give an example of a COS for Work: core outcomes for every stage of work participation.
4. Ensure that all participants understand their roles and their tasks for the Delphi voting
5. Discuss which are the most important outcomes from the preliminary for every group of stakeholders
6. Present any additional core outcomes we received from the stakeholders (if more than two stakeholders provided they via mail prior to the discussion)

The discussions will take two hours per group. We will divide the participants in two groups to facilitate participation across all times zones. The first group will be from 09:00 till 11:00 CET and include participants from Europe, Asia and Australia. The second group will be from 17:00 to 19:00 CET and include participants from Europe, Canada, North America and South America.

The participants will receive a Zoom link. In the e-mail invitation we will state that the session will be recorded unless anyone wishes to object. We will discuss the topics as outlined in the aims of the discussion using the background document. Participants will have the opportunity to provide input on each topic. The chair, Carel Hulshof, will facilitate the discussion and the other members of the steering group will facilitate the chat and the poll functions. The chair will work with a meeting

For peer review only - http://bmjopen.bmj.com/site/about/guidelines.xhtml

manual prepared by the steering group. The results of this online discussion will serve as input for the first Delphi round.

3. First round of Delphi voting

Objective: get a preliminary agreed upon set of core outcomes for each stage of work participation

If for some reason participants were not able to attend the first discussion round they may take part in the Delphi voting if they familiarized themselves with the background document. Any written feedback will be taken into consideration by the steering group as input for the Delphi voting.

The list of the outcomes for every stage of work participation will be agreed upon during the discussion. If more than one stakeholder wants to add an outcome which was not part of the preliminary set we will add the outcome to the Delphi rounds.

Voting procedures and interpretation of the findings:

Participants will receive a link to register as a stakeholder for the DelphiManager software (provided by COMET) and vote on which categories and outcomes within the four stages of work participation they deem as core outcomes.

Quantitative data will be collected and provided to participants to inform the ranking of outcomes. The DelphiManager includes ranking based on a Likert Scale from 1-9; from not important to critical. An “unable to score” option is also given.

Consensus on outcomes to include in the core outcome set is when ≥ 80% of participants score an outcome as critically important (score 7 to 9) and consensus to not include an outcome as core is when <50 % of participants score outcomes with 7-9. However, it is possible that too many outcomes may have consensus to include for a core outcome set and some important outcomes may receive low scores due to lack of awareness of their importance by the majority of stakeholders. Therefore we will use the consensus definition, as described above, as a guideline and provide the participants with the opportunity to elaborate on their choices during the second discussion.

4. Online discussion of the results of the first Delphi round

The aim of the second discussion will be to go through the results of the first voting round. Outcomes with most agreement (score 7 to 9) will be presented and discussed. Clarifications of the outcomes and opinions on why certain outcomes deserve priority will be discussed. Any strong arguments from stakeholders against high scoring outcomes and potential “downgrading” of high scoring outcomes will be discussed. Outcomes which were not ranked as critical (<50 % of participants score outcomes with 7-9) will also be discussed and the participants will provide input whether all or some these outcomes can be dismissed from the second round of voting. Based on this discussion the steering group will consider the argumentation of the stakeholders and decide which outcomes will be included in the second Delphi round.

For peer review only - http://bmjopen.bmj.com/site/about/guidelines.xhtml

5. Second Delphi voting round

The objective of the second Delphi round will be to reach agreement by stakeholders on the final set of core outcomes for each stage of work participation. In the second Delphi round the participants will see the scores from other participants on every outcome within each of the four stages and re-rate the outcomes which were considered critical after the first round of voting and the second discussion round.

DATA ANALYSIS

The data from the Delphi rounds will be processed anonymously but in the discussion rounds the participants will be identifiable.

Group discussions

We will summarize the main take-aways from the group discussion on every topic and hold a meeting with the steering group on how we will process the input for the Delphi study. For the topics which require voting, at least 70% agreement will be required for an argument to be considered as a “yes”.

Delphi voting rounds

The results from the Delphi rounds will be extracted from the Delphi Manager software into a CSV format and analyzed in SPSS using descriptive statistics. We will report measures of the distribution scores for all outcomes which will be included in the final round. If too many outcomes will have agreement on inclusion, outcomes with the highest mean scores within each stage of work participation will be selected as core.

RESULTS

Results will be processed anonymously. Qualitative data from the discussions with the stakeholders will be summarized. Quantitative data from the voting rounds will include distributions of the scores for all outcomes and the number of participants who scored the outcomes.
In the following project the steering group will give recommendations on how to best measure the selected core outcomes. The results will be reported in an article to be submitted to an international peer reviewed journal. After the publication we will share the results with the participants and disseminate further within the research community.

For peer review only

Reference list

1. Hoving JL, Lacaille D, Urquhart DM, Hannu TJ, Sluiter JK, Frings-Dresen MH. Non-pharmacological interventions for preventing job loss in workers with inflammatory arthritis. Cochrane Database of Systematic Reviews. 2014(11).
2. de Boer AG, Taskila TK, Tamminga SJ, Feuerstein M, Frings-Dresen MH, Verbeek JH. Interventions to enhance return-to-work for cancer patients. Cochrane database of systematic reviews. 2015(9).
3. Nieuwenhuijsen K, Faber B, Verbeek JH, Neumeyer-Gromen A, Hees HL, Verhoeven AC, et al. Interventions to improve return to work in depressed people. Cochrane Database of Systematic Reviews. 2014(12).
4. Kirkham JJ, Gargon E, Clarke M, Williamson PR. Can a core outcome set improve the quality of systematic reviews?–a survey of the Co-ordinating Editors of Cochrane Review Groups. Trials. 2013;14(1):21.
5. Ravinskaya M, Verbeek JH, Langendam M, Daams JG, Hulshof CT, Madan I, et al. Extensive variability of work participation outcomes measured in randomized controlled trials: a systematic review. Journal of Clinical Epidemiology. 2022;142:60-99.
6. Ravinskaya Margarita; Verbeek JHL, Miranda; Hulshof, Carel TJ; Madan, Ira; Verstappen, Suzanne MM; Kunz, Regina; Hoving, Jan L. Preferred methods of measuring work participation. An international survey among trialists and Cochrane systematic reviewers. "In press". 2022.
7. Sinha IP, Smyth RL, Williamson PR. Using the Delphi technique to determine which outcomes to measure in clinical trials: recommendations for the future based on a systematic review of existing studies. PLoS medicine. 2011;8(1):e1000393.

For peer review only

For peer review only - http://bmjopen.bmj.com/site/about/guidelines.xhtml

Development of a core outcome set for work participation:

COS for Work

Introduction on COS for Work and instructions for the Delphi study

Steering group

Dr. Jan L. Hoving - Amsterdam UMC, location Academic Medical Center, University of Amsterdam, Coronel Institute of Occupational Health, Amsterdam Public Health research institute, Amsterdam, NL

Margarita Ravinskaya - Amsterdam UMC, Academic Medical Center, University of Amsterdam, Coronel Institute of Occupational Health, Amsterdam Public Health research institute, Amsterdam, NL

Dr. Jos H. Verbeek MD - Amsterdam UMC, location Academic Medical Center, University of Amsterdam, Coronel Institute of Occupational Health, Amsterdam Public Health research institute, Amsterdam, NL

Dr. Miranda W. Langendam - Amsterdam UMC, location Academic Medical Center, University of Amsterdam, Department Epidemiology and Data Science, Amsterdam Public Health research institute, NL

Prof. Ira Madan, MD, FRCP, FFOM – Guy's and St Thomas' NHS Trust and Faculty of Life Sciences and Medicine, King's College London, Centre for Musculoskeletal Health and Work, UK

Prof. Dr. Regina Kunz, MD - Research Unit EbIM, Evidence Based Insurance Medicine, Department of Clinical Research, University of Basel, CH

Dr. Suzanne M.M. Verstappen - [1] Centre for Epidemiology Versus Arthritis, Faculty of Biology, Medicine and Health, The University of Manchester, UK, [2] NIHR Manchester Biomedical Research Centre, Manchester University NHS Foundation Trust, Manchester Academic Health Science Centre, UK, [3] MRC Versus Arthritis Centre for Musculoskeletal Health and Work, University of Southampton, UK

Prof. Dr. Carel T.J. Hulshof, MD - Amsterdam UMC, location Academic Medical Center, University of Amsterdam, Coronel Institute of Occupational Health, Amsterdam Public Health research institute, Amsterdam, NL

1. Introduction

1.1 What is this document about?

You have accepted our invitation to take part in the consensus process to establish a core outcome set for work participation (COS for Work). Thank you for taking the time to collaborate with all of our international colleagues in the field of occupational health and related areas. In this document we explain the need for and purpose of COS for Work, how the consensus process will work, and what is expected of you.

1.2 What is a COS?

A COS is an agreed *minimum* set of outcomes that should be measured consistently in all studies in a specific health area to make study findings on similar topics more comparable. These core outcomes are chosen and defined by key stakeholders, for example health professionals, researchers, patient population and policy makers.

Core outcome sets have the following benefits: (1) facilitate large scale evidence synthesis by systematic reviews, (2) provide clearer answers to the question of what works best, (3) support informed decision making in clinical practice and policy, (4) reduce reporting bias and research waste, and (5) ensure that the outcome

measures selected are truly critical to key stakeholders[1] . A common misconception about the use of core outcome sets is that studies should *only* include the outcomes from the COS. This is not the case: rather, any other primary and secondary outcomes can be measured *alongside* the outcomes from a specific COS.

The current heterogeneity in measuring work participation (WP) hinders a full understanding of the findings across similar studies. A small set of WP outcomes used in all studies would close this gap. As of date such a generic set of study outcomes is lacking. This project aims to create a COS for Work. We consider this project to be a highly relevant contribution to research methodology of occupational health. It is a starting point. Further iterations will refine and improve the COS over time.

1.3 The purpose COS for Work

Even though COS can be developed for practice and/or research, this COS for Work is intended for research purposes only. The aim is to assist researchers in choosing which outcomes to measure in effectiveness studies to improve WP for people with health problems. Our COS for Work will be a generic COS (in contrast to COS that are developed for a specific disease). This means, it intends to evaluate any type of WP intervention in people with any type of health problem.

In general, two types of interventions can be distinguished: (1) vocational interventions with direct impact on work participation, such as Individual Placement and Support studies, and (2) non-vocational interventions, i.e. any type of clinical, pharmacological, psychological study with an intervention to improve health. For non-vocational interventions the effect on work participation is often a secondary objective and work outcomes are measured as secondary outcomes.

1.4 Our preparatory studies for COS for Work

To lay the groundwork for the consensus process, we conducted three studies. The full text versions are attached to the e-mail. Reading is optional but highly recommended. Below is a summary of the aims and findings of the three papers.

1. **Extensive variability of work participation outcomes measured in randomized controlled trials: a systematic review.** **https://doi.org/10.1016/j.jclinepi.2021.10.013 – Published**
We performed a systematic review which showed how WP outcomes are measured across randomized controlled trials (RCTs) aimed to impact WP directly or indirectly. We found a great deal of heterogeneity on the level of outcome terminology and measurement methods. We grouped outcomes in four main categories: "employment status", "absence from work", "at work productivity loss" and "employability".

2. **Preferred methods of measuring work participation. An international survey among trialists and Cochrane systematic reviewers.** **https://doi.org/10.1007/s10926-022-10031-0 - Published**
We contacted the authors of the RCTs included in the above review and the authors of Cochrane systematic reviews to explore their reasons for choosing specific outcomes and measurement methods. Our findings show that the authors based their choice of measurement methods mainly on previous similar studies, that feasibility is the most important factor in the choice of measurement method, and that a COS for Work is considered very useful for research. In addition, the authors indicated that the most important outcomes for a COS for Work would be "employment status" and "sickness absence" and "return to work". Their preferred follow-up period was 12 months across all outcome categories.

---

[1] Williamson PR, Altman DG, Bagley H, Barnes KL, Blazeby JM, Brookes ST, et al. The COMET handbook: version 1.0. Trials. 2017;18(3):280.

For peer review only - http://bmjopen.bmj.com/site/about/guidelines.xhtml

3. **A general framework for selecting work participation outcomes in intervention studies among persons with health problems.** https://doi.org/10.21203/rs.3.rs-1342930/v1 - Under review

As work participation is an umbrella term containing different types of outcomes, we looked for a comprehensive framework that could be used for COS for Work. Since there was no pre-existing framework, we developed one for COS for Work. The framework includes the following four stages of WP: (1) initiating employment, (2) having employment, (3) increasing or maintaining productivity at work and (4) return to employment. These work participation stages should help our consensus process to select the outcome concepts and outcome measures. Furthermore, we defined criteria for COS outcomes that are part of the document for selecting outcomes in the Delphi study.

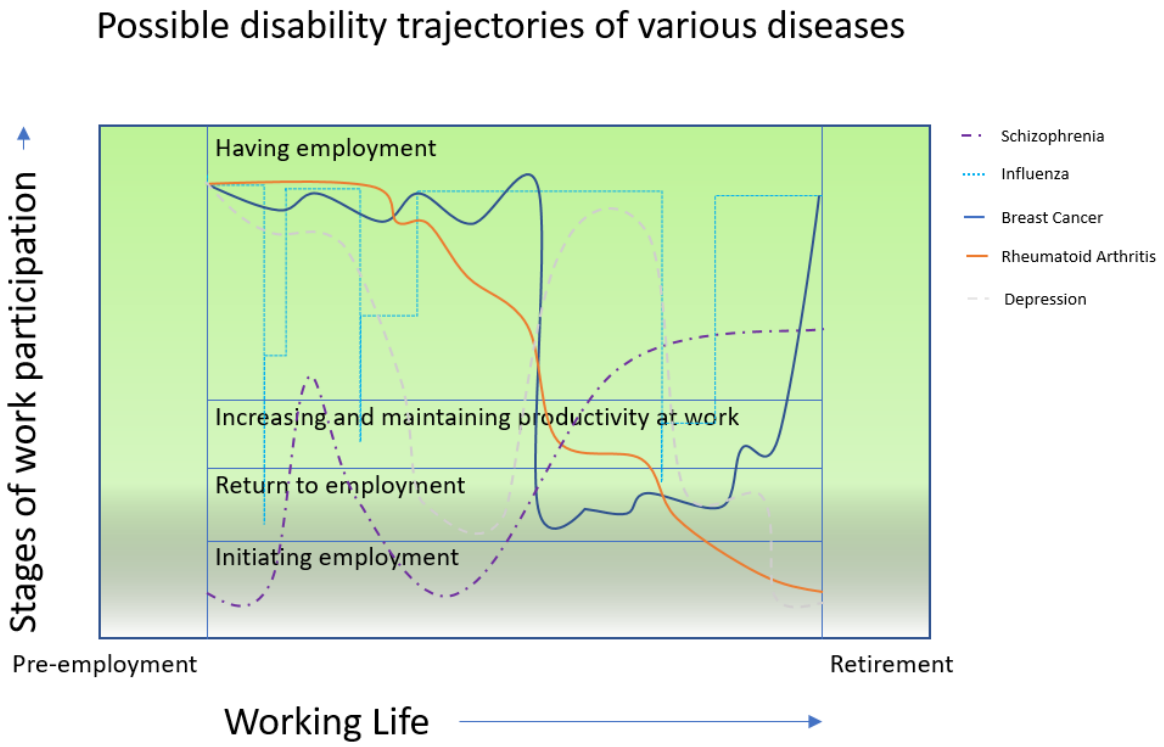

This figure is adapted from the COS framework. Here we illustrate how possible disability trajectories result from various health problems. The four work participation stages can be experienced during the course of the working life.

When linking the 24 commonly used outcomes to the four stages, 10 outcomes referred to 'Return to employment', 5 outcomes to 'Increasing or maintaining productivity at work', 7 outcomes to 'Having employment' and 2 outcomes to 'Initiating employment'. Many of these outcomes can be refined in more details, e.g. by specifying the timing (1 day, 4 weeks, 12 months) or type or amount of work (full time, part-time, last job, any work).

2. Instructions for the Delphi Study

<u>2.1 Purpose of the study</u>

The purpose of this consensus study is to identify a core set of outcomes (COS) that captures work participation and that can be used in intervention studies at each of the four stages of work participation

identified in our framework. Please note that discussion and votes in this study do not address measurement instruments.

The study includes 4 activities: Two online consensus discussions and two rounds of Delphi voting.

1. First online group discussion (20 April 2022)
2. Delphi I voting (11 – 14 May 2022)
3. Second online group discussion about the results of the first Delphi vote (25 May 2022)
4. Delphi II voting (30 May – 1 June)

We seek a minimum set of outcomes that are meaningful and easy to measure ('feasible') by all studies in the field. There are no strict rules for the number of outcomes a COS may contain, but the current 24 outcomes would defeat the purpose. Compromises may need to be made in the number and type of outcomes.

2.2 Organization

a) Selection of participants

To cover core outcomes relevant to a broad range of stakeholders, we invited experts from diverse professional groups. We have assigned you to one of the following stakeholder groups: researcher, policy maker, employer representative, employee representative, occupational health professional (see document "COS Delphi participants_ stakeholder groups"). Please use the perspective of your assigned stakeholder group in your country when discussing and selecting the core outcomes. Some of you fulfill more than one role in practice or have done so in the past. If for any reason you disagree with the group you have been assigned to, please let us know.

b) Running the meeting

Chair: Prof. Carel Hulshof will chair the meetings, and the COS for Work team will assist the process.

Protocol: To be transparent on how consensus is reached, we are working with a protocol that is publicly available on the COMET website (https://www.comet-initiative.org/studies/details/1195).

Video recording: We will record the online meetings for study purposes only. The recordings will be destroyed before 31 December 2022.

c) Ground rules for communication

All stakeholders should have the opportunity to voice their opinion. As the time for discussion will be limited, please try to present your arguments briefly and succinctly so that others can also put forward their arguments, thus facilitating the exchange.

d) Contact for queries

If anything is unclear, please do not hesitate to contact us.

Contact: Margarita Ravinskaya, m.ravinskaya@amsterdamumc.nl

2.3 The activities

1. First online group discussion

When: 20 April 2022

Zoom link

Connect to the discussion via the following link: https://uva-live.zoom.us/j/9294218837

Aims of the session

For peer review only

1. Ensure that all participants understand their roles and their tasks for the discussions and the Delphi votes.
2. Present the COS for Work framework.
3. Make explicit that each stage of work participation has more than one outcome concept. We are seeking consensus on outcome concepts and the core outcomes belonging to each stage for most stakeholders.
4. Present additional core outcomes that may have been submitted by at least two stakeholders.
5. Discuss the most important outcomes from the preliminary outcome set (document "Delphi voting: Stages and Outcomes"), first in breakout rooms and finally in plenary setting.

Preparatory work for the participants

1. Familiarize yourself with the document "Delphi voting: Stages and Outcomes" (see email attachment) to be used for the Delphi rounds. The preliminary list of outcomes per WP stage are based on the outcomes most commonly measured in our systematic review (see email attachment). If you miss crucial outcomes, please send us those outcomes and your reasons, as soon as possible but 18 April 2022 at the latest.
2. Write your preliminary selection of core outcomes in the worksheet table in the document. Please specify the minimum of outcomes that you think should be included in COS for Work.

   In this project, we aim to achieve consensus on outcomes only (not on measurement instruments). While choosing your preferred core outcomes, please write down your rationale for doing so. For example, in our survey reasons for choosing a certain outcome were their use in similar studies, relevance for the study population, or easy access to registry data. Discussing and balancing preferences for specific outcomes will facilitate the final selection.

Definition "Outcome Measure" versus "Measurement Instrument"

Please note the difference between "outcome concept", "outcome measure" and "measurement instrument" (or tool or method). "Outcome concept" is an umbrella term for the many facets of a certain concept. *An outcome is WHAT we aim to measure. A measurement instrument is the method HOW the outcome is measured.*
The Delphi voting will refer to the colored columns only (NO VOTING on the measurement instrument)

Outcome concept	Outcome measure (Description)	Measurement instrument (Example)
Work ability	Current self-rated physical and mental work ability compared to lifetime best	The Work Ability Index

3. Recommended but optional: familiarize yourself with our three background studies (systematic review, survey, and framework, see email attachment).

2. Delphi I: Individual online voting

When: 11 – 14 May 2022

Individual assignment for all participants

Use the DelphiManager software to select and rank outcomes and to motivate your selection and ranking. Please complete this task between 11 and 14 of May 2022.

Overall group aim:

- Reach an initial consensus on a set of core outcomes for each stage of work participation.

The DelphiManager software

We will use the DelphiManager software to vote on the outcomes for the four stages of WP. You will receive a link to register for the DelphiManager software.

The DelphiManager includes a ranking of the outcomes on a Likert Scale from 1-9: from 'not important' to 'critical', plus an 'unable to score' option. The results will be summarized and reflected in the second discussion round and a refined set of outcomes will be presented in the second Delphi vote.

3. Second online group discussion

When: 25 May 2022

Zoom link

Connect to the discussion via the following link: https://uva-live.zoom.us/j/9294218837

Overall group aim:

- Discuss the results of the first Delphi voting round with other stakeholders.

We will discuss the outcomes with the highest agreement (score 7 to 9) and the rationale on why certain outcomes deserve priority. Any strong arguments from stakeholders against high scoring outcomes and potential "downgrading" of high scoring outcomes will be discussed. Outcomes not ranked as critical (<50 % of the participants scored the outcomes with 7-9) will be reviewed to determine whether some or all of these outcomes can be dismissed from the second round of voting. Based on the arguments of the stakeholders in this discussion, the COS for Work team will make the final decision on which outcomes to include in the second Delphi vote.

4. Delphi II : Individual online voting

When: 30 May – 1 June 2022

Overall group aim:

- Reach consensus on the final set of core outcomes for each stage of work participation.

In the second Delphi voting round, participants see the scores of the other participants for each outcome in all four stages. Using DelphiManager software, you will re-rate all outcomes that were considered critical after the first Delphi round of voting and the second round of discussion.

For peer review only

For peer review only

Suggested outcome(s)	Stage	Argumentation	Other remarks
**"Work environment" Availability/access to appropriate work modifications/supports**	Increasing or maintaining productivity	This is an issue that we have identified at least for physical disability groups that we have surveyed/interviewed. This also ensures a 'biopsychosocial approach' – in terms of CoS identifying personal factors but also factors in the social environment that are critical to RTW.	
**Work-life balance**	Increasing or maintaining productivity	We notice in people with Parkinson's they initially keep up productivity at work at cost of participation in other life roles. You partly measure this with 'need for recovery'… But would you consider including a measure about satisfaction with participation (e.g. USER-P- satisfaction scale?)	
**Additional perceptions affecting productivity**	Increasing or maintaining productivity	Are (self)-perceived stigma and perceived sef-efficacy at work covered in the concepts you describe?	
			A general problem I have with a lot of the current PROMS: they all assume the person is employed; some questions are not appropriate for people who are self-employed.
**Perceived competences**	Unclear		
**Impact of the disease on the career**	Unclear		
			The first question is that we probably should define the scope : individual level (e.g. effect of an intervention for people with cancer or depression) of collective level (effect of preventive measures on the rate of sick leave in a company),

			in order to priorize the COS
			The contentment for the job is mentioned but there is no COS for this
			Work ability index : effect of the disease or of aging ? We should include age in the analysis since the WAI may decrease with age, independently from the potential onset of diseases
**Predefined type of occupation**	Having employment	Sometimes people want to work in an occupation that meets their values more than money or time.	
**Predefined commute time**	Having employment	Work is a social activity for humans in the COMMUNITY. In some countries, it can take a long time to get to the workplace. Also, with regard to mental illness, people may be hesitant to work near their homes due to stigma. Therefore, matching commuting time seems to be important.	
**Predefined illness disclosure status**	Having employment	It would be important to make your own decisions about disclosure or nondisclosure for mental illness, HIV/AIDS, LGBT, and other conditions that are highly stigmatized.	
**Job satisfaction**	Having employment	Ultimately, job satisfaction appears to be most important, while measuring this outcome needs further discussion and scientific evidence.	
Lost working days <br>**(please note: I propose instead a slightly different measure: *working time of observed time ~ working days of days lived/observed*)**	Return to Employment	Basically, this measurement is sensitive to almost all core aspects of most other proposed measurements: time to RtW, sustained RtW and	

For peer review only

		risk of reoccurrence of sick leave:<br><br>It is sensitive to both sick leave duration of index sick leave *and* any reoccurrence of sick leave as well, and the length and timing of both of these events. This is contrary to *time to RtW (1.1.2), sick leave duration (1.2.5)* that are not sensitive to reoccurrence and *time to RtW… (1.1.4)* that is not sensitive to relapse beyond the specified threshold period (e.g. 4 weeks).	
Time to return to sustainable work, with high threshold (e.g. four consecutive months)	Return to Employment	Time to return to work (or any other positive event) is often not sensitive to reoccurrence – this problem can be handled by defining the threshold of sustainability rather high, like e.g. four months. The threshold of sustainability should be high enough, so that any reoccurrence after this threshold would not change the perception that the previous return to work was harmful, and retrospectively not recommended.	
**Skills for job procurement, externally assessed**	Initiating employment	Self-reported skill level is biased, why it should be externally assessed. Yet, job skills are most important for work participation, since work implies other stakeholders (costumers, employers etc.) should perceive work or services to have been delivered to pay money (which requires skills!)	
			I send you my proposals, but *I am not sure*, whether my

For peer review only

			choices/preferences are "missing outcomes" or just already mentioned outcomes. The reason for my doubts are thate the adjustments I propose might be so slight that the outcomes do not count as other and hence missing outcomes.
**Employment status operationalization**	Having employment	a) Labour market status during the last week of each year<br><br>OR<br><br>b) The main activity (i.e., labour market status) during the year<br><br>OR<br><br>c) The measure of labour market status, based on labour market status during each month.<br><br>###<br><br>a) and b) are strongly correlated.<br><br>We have used almost always a).	
**"Having lost employment" - early retirement**	Having employment	This seems to also be an important outcome	
**Time until employment since finishing school/vocational training**	Initiating employment	Would also be a more objective and internationally comparable outcome.	

For peer review only

For peer review only

Introduction

In this document we present a list of work participation outcomes measured randomized controlled trials. The outcomes are sorted according to the stage of work participation.
The stages are presented in the order of most to least commonly measured outcomes.

First, you will find a brief explanation of every stage. In particular we describe for which target group and intervention types outcomes belonging to each stage are relevant. We also provide some additional information for every stage. The "criteria to consider for COS outcomes" were developed as part of the framework. They are mostly important on the level of measurement. We will use these criteria after the Delphi study to determine how each COS outcome resulting from this Delphi study can be measured. We include the criteria for your information, so you may take them into consideration (as far as possible) when selecting most important outcomes.

For every stage you will find a preliminary set of outcomes. The "outcome concept" refers to a category of outcomes within each stage. The "outcome measure" is the list of outcomes which will be used to vote on in the Delphi rounds. In addition, we provide examples of measurement tools, instruments or methods.

Please consider which outcome measures you find most important and complete the worksheet belonging to every stage. It will be helpful if you also note the outcome ID you are referring to. This will be the basis of our first round of Discussion on 20 April 20 2022.

If you miss an important outcome concept or outcome measure, please send us the outcome and the reason of its' importance. **Please do so 18 April 2022 at the latest**, in that way we can include the additional outcomes in the first discussion on 20 April.

Stage 'Return to Employment' & Sick leave from work

This is a stage of work participation when employed people (temporarily) stop attending work and are on sick leave. The ICF does not include sick leave or sickness absence, but potentially outcomes for this stage could fit under "maintaining employment" and "remunerative employment" ICF categories. Work related rehabilitation interventions designed to help workers return to work include outcomes relevant to this stage as their primary outcome. Any type of clinical, pharmaceutical or otherwise health related intervention may be expected to indirectly impact sick leave. Sickness absence can show that a person is unable to fulfil their worker role due to ill health, but other reasons for absenteeism, such as maternity leave or unwillingness to come to work also exist. Evaluation of absenteeism outcomes is often approached from the societal/economic perspective. Sick leave is often converted into a monetary value as part of economic evaluations to indicate extra costs for the employer or/and the employee.
Measuring changes in sick leave is not straightforward as the denominator *can refer to all workers or to workers that are on sick leave*. It is, therefore, very important to be clear to which group the sick leave figures refer.
More recently, the term 'return to work' has been introduced as an indicator of sickness absence from an individual perspective. For individuals, the worker role is important for economic reasons but also for reasons of well-being. For example, cancer survivors report that getting back to work is a final step in getting back to normal life after their disease/treatment experience.

Additional information to consider for this stage

- Outcomes within this stage of work participation are measured most frequently (45% of all RTC's in our review) in intervention studies which directly or indirectly aim to impact work participation
- In our survey, 93% of RCT authors and 69% of Cochrane systematic reviewers indicated that outcomes belonging to this stage should be in COS for Work
- Registry data is preferred by researchers, but type and definition of sickness benefits registry data varies per country and may be inaccurate about the diagnosis

For peer review only - http://bmjopen.bmj.com/site/about/guidelines.xhtml

- Time to RtW as a standalone measure may not be a meaningful outcome about sustained effect of an intervention (recommendations are made to also measure duration of sick leave, sick leave spells and proportion of employees who return to part/full time work)

Criteria for COS outcomes

Criteria to consider	***Optional criteria***
Outcomes should:<br>1. Be sensitive to change<br>2. Be feasible to measure<br>3. Be applicable internationally<br>4. Be work participation specific<br>5. Capture multiple stakeholder perspectives<br>6. Be in alignment with the ICF model	*Outcomes should:*<br>1. Be used for cost-effectiveness studies<br>2. Be applicable across varying insurance schemes

**ID**	**Outcome concept**	**Outcome measure (description)**	**Measurement instrument (examples)**
1.1.1	Return to work	*Proportion of workers that return to work* after being absent because of an illness	- to any type of work<br>- to specific type of work<br>- to a defined amount of work<br>- at a defined point in time (e.g. at least 1 day, 3 days, 4 weeks, 12 months)
1.1.2		*Time to return to work* or duration of sickleave after being absent because of an illness	- to any type of work<br>- to specific type of work<br>- to a defined amount of work<br>- at a defined point in time (e.g. at least 1 day, 3 days, 4 weeks, 12 months)
1.1.3	Sustainable Return to work	*Proportion of workers that return to work and remain at work for a specified amount of time*	e.g., 4 weeks without relapse of sick leave at a specific moment of follow-up e.g. 12 months
1.1.4		*Time to return to work in workers absent because of an illness and who do not relapse within a specified amount of time*	e.g., 4 weeks at a specific moment of follow-up e.g. 12 months
1.1.5	Return to work: the workers perceptions	*Time it will take to return to work as perceived by the sick worker;* also described as self-efficacy for return to work, return to work expectations or intention to return to work	- Work Readiness Questionnaire (WoRQ)<br>- Inventory for Job Coping and Return Intention (JoCoRi)

1.2.1	Sickness absence *among all workers* in an organization/group	*A. Lost working days*<br>*B. Lost sick days*	A. Percentage of all working days of all workers in an organization during a time period e.g. 12 months<br>B. Sick days as percentage of all available days during a time period
1.2.2		*Sick leave duration*	- Working days absent because of illness averaged overall workers<br>- Calendar days sick
1.2.3		*Sick leave frequency*	Number of episodes of sick leave per worker expressed as percentage

1.2.4	Sickness absence *among absent workers* in an organization/group	*a) Lost working days*<br>*or*<br>*b) lost sick days*	a) percentage of all working days during a time period e.g. 12 months;<br>b) sick days as a percentage of all available days during a time period
1.2.5		*Sick leave duration*	• working days absent averaged over all sick leave episodes; can also be expressed as calendar days sick;<br>• is numerically identical to *time to return to work* among workers absent from work

Worksheet for participants

**Outcomes measures for 'Return to Employment'**	**Reasons for selecting these outcomes** *(Examples: previously used in similar studies, relevance for the study population, easy access to data, e.g. registry data, ...)*
**Step 1: Select all outcomes that you feel are important**	
a.	
b.	
c.	
d.	
e.	
f.	
g.	
**Step 2: Note the outcomes that you consider to be the bare minimum for COS for Work**	
a.	
b.	
c.	
d.	
e.	
f.	
g.	

Stage 'Increasing or maintaining productivity at work'

This stage of work participation occurs when people experience limitations or impairments whilst working which results in lower quality or lower output of work. The input versus output is often used to calculate cost. In terms of ICF, the outcomes could be placed under the ICF-category "Maintaining a job".
Outcomes measure relevant for this stage are for any type of vocational or clinical/pharmaceutical intervention that may impact on overall productivity, work ability and work functioning of people with a health problem.
From the worker perspective, feeling fit to work is essential for a successful worklife. Employers can offer interventions to reduce stress and increase wellbeing in the workplace as preventive measures.

Additional information to consider for this stage

- Outcomes for this stage were measured second most frequently in the review; 30% of all RCTs measured an outcome relating to productivity.
- 63% of the RCT authors and 75% of Cochrane reviewers indicated in the survey that measuring outcomes for this stage should be part of the core outcome set.

Criteria for COS outcomes

Criteria to consider	***Optional criteria***
Outcomes should:<br>1. Be sensitive to change<br>2. Be feasible to measure<br>3. Be applicable internationally<br>4. Be work participation specific<br>5. Capture multiple stakeholder perspectives<br>6. Be in alignment with the ICF model	*Outcomes should:*<br>1. Be used for cost-effectiveness studies<br>2. Be applicable across varying insurance schemes

**ID**	**Outcome concept**	**Outcome measure (Description)**	**Measurement instrument (Examples)**
2.1.1	Work productivity loss (economic evaluation)	Overall loss of productivity at work (in terms of quality or output at work) resulting from presenteeism or absenteeism regardless of cause.<br>Typically used for economic evaluations	Work Productivity and Activity Impairment Questionnaire
2.2.1	Presenteeism	Not being productive while at work due to health problems.<br>Typically used to evaluate the effect of health interventions for individuals	Stanford Presenteeism Scale
2.3.1	Work ability	Current self-rated physical and mental work ability compared to lifetime best. Often used for prognosis of RtW, however the commonly used tool (WAI) is not effective for identifying changes of work ability after RtW	Work Ability Index (WAI)
2.4.1	Work activity impairment	Experienced functional impairment of performing work activities due to a health problem.	Work Limitations Questionnaire (e.g. WLQ 25)
2.5.1	Perceptions affecting productivity	Beliefs in working capacity, motivation for work, vocational commitment, job coping, contentment with work, need for recovery	- Subjective Prognosis of Work Capacity<br>- Work Motivation Scale<br>- Need for Recovery Scale<br>- Job Coping (JoCoRi))

Worksheet for participants

**Outcomes measures for 'Increasing or maintaining productivity at work**	**Reasons for selecting these outcomes** *(Examples: previously used in similar studies, relevance for the study population, easy access to data, e.g. registry data, ...)*
**Step 1: Select all outcomes that you feel are important**	
a.	
b.	
c.	
d.	
e.	
f.	
g.	
**Step 2: Note the outcomes that you consider to be the bare minimum for COS for Work**	
a.	
b.	
c.	
d.	
e.	
f.	
g.	

Stage 'Having employment'

Outcomes relevant for this stage of work participation measure whether a person is in employment, can retain employment, loses work or retires within the duration of the study. In general, these outcomes fit in the ICF categories under "Remunerative Employment" and "Keeping a Job". Being employed can be considered as having a contract with an employer or being self-employed. Both, vocational or non-vocational interventions, are relevant for this stage. Most studies on vocational rehabilitation have the primary aim to determine the effect of an intervention on employment status. Medical or pharmaceutical studies are more likely to measure the effect of the intervention on employment status as secondary outcome. The target group of these interventions are people who are known to be at risk of losing employment due to a health problem.

Additional information to consider for this stage

- Our review showed that 16% of all measured outcomes belonged to this stage.

- 98% of RCT authors and 100% of Cochrane reviewers agreed that outcomes for this stage of work participation should be part of COS for Work.
- Most of the measurement tools used to measure outcomes at this stage are not validated, such as unspecified (self-developed) items, interviews, and phone calls. The exception is data from registries such as those in the Nordic countries.

Criteria for COS outcomes

Criteria to consider	***Optional criteria***
Outcomes should:<br>1. Be sensitive to change<br>2. Be feasible to measure<br>3. Be applicable internationally<br>4. Be work participation specific<br>5. Capture multiple stakeholder perspectives<br>6. Be in alignment with the ICF model	*Outcomes should:*<br>1. Be used for cost-effectiveness studies<br>2. Be applicable across varying insurance schemes

**ID**	**Outcome concept**	**Outcome measure (Description)**	**Measurement instrument (Examples)**
3.1.1	Employment	Having any type of employment including self-employment	
3.1.2	Employment	Having met a pre-defined status of employment; part/full time, competitive work in a mainstream setting, performing the same tasks as non-disabled workers	
3.1.3	Employment	Predefined type or amount of income	
3.1.4	Employment	Duration of employment	
3.2.1	Employment	Having lost employment	
3.3.1	Work disability	Permanent and complete inability to engage in any work participation	
3.3.2	Work disability	Permanent partial disability to engage in work participation	

Worksheet for participants

**Outcomes measures for 'Having employment'**	**Reasons for selecting these outcomes**<br>*(Examples: previously used in similar studies, relevance for the study population, easy access to data, e.g. registry data, ...)*
**Step 1: Select all outcomes that you feel are important**	
a.	

b.	
c.	
d.	
e.	
f.	
g.	
**Step 2: Note the outcomes that you consider to be the bare minimum for COS for Work**	
a.	
b.	
c.	
d.	
e.	
f.	
g.	

Stage: Initiating employment

Outcomes within this stage measure whether participants are prepared for initiating employment. The target group are people who are unemployed and seeking employment (i.e., not self-employed or contracted by an employer, however, possibly with a type of subsidized governmental wage replacement benefit). This stage is particularly applicable to those who never worked, for example due to just completed education. ICF categories "Apprenticeship" (work preparation) and "Acquiring a job" apply. Interventions for this category focus on increasing skills, knowledge or attitude of participants for successful engagement in a workers' role.

Additional information to consider for this stage

- The outcomes belonging to this stage were measured least frequently in our review, in about 5% of all trials.
- In our survey, 59% of RCT authors and 75% of Cochrane reviewers indicated that outcomes from this category should be part of the COS.
- These outcomes have been shown to be a predictor for employment status.

Criteria for COS outcomes

Criteria to consider	***Optional criteria***
Outcomes should:	*Outcomes should:*

Criteria to consider	*Optional criteria*
7. Be sensitive to change<br>1. Be feasible to measure<br>2. Be applicable internationally<br>3. Be work participation specific<br>4. Capture multiple stakeholder perspectives<br>5. Be in alignment with the ICF model	1. Be used for cost-effectiveness studies<br>2. Be applicable across varying insurance schemes

ID	Outcome concept	Outcome measure (Description)	Measurement instrument (Examples)
4.1	Skills for job procurement	Total work-related network, Engagement in work seeking activities, Job interview skills, Work-related benefit of vocational training	- Work section of the Social Adjustment scale<br>- Mock Interview Rating Scale
4.2	Self-efficacy	Self-efficacy for job procurement, Hope, optimism and self-efficacy for achieving vocational success, Social/interpersonal self-efficacy	- Job Procurement Self-efficacy Scale<br>- The Work Hope Scale<br>- Subjective Prognosis of Work Capacity

Worksheet for participants

**Outcomes measures for 'Initiating employment'**	**Reasons for selecting these outcomes**<br>*(Examples: previously used in similar studies, relevance for the study population, easy access to data, e.g. registry data, ...)*
**Step 1: Select all outcomes that you feel are important to include in COS for Work**	
a.	
b.	
c.	
d.	
e.	
f.	
g.	
**Step 2: Note the outcomes that you consider to be the bare minimum for COS for Work**	
a.	
b.	

c.	
d.	
e.	
f.	
g.	

For peer review only

For peer review only

This document contains 35 additional outcomes which were suggested by participants in the first round of Delphi voting. We consider whether the suggested outcomes match the criteria for outcomes suitable for COS for Work.

We would also like to draw your attention to the overarching definition of work participation used for COS for Work: *"work participation entails either performing work related activities or having the capacity to do a work related activity".* This definition is based on the definition of participation provided by the International Classification of Functioning, Disability and Health (ICF). The operationalization of this definition is not straight forward but the outcomes have to measure at the level of (not) performing a work related activity.

In addition, we use the criteria for outcomes suitable for the COS which were developed as part of the COS for Work framework.

For more reading on defining work participation, operationalization of outcomes and of the criteria, the framework can be accessed via **https://doi.org/10.21203/rs.3.rs-1342930/v1**

COS for Work outcomes criteria.

Criteria to consider	***Optional criteria***
Outcomes should:<br>1. Be sensitive to change<br>2. Be feasible to measure<br>3. Be applicable internationally<br>4. Be work participation specific<br>5. Capture multiple stakeholder perspectives<br>6. Be in alignment with the ICF model	*Outcomes should:*<br>1. Be used for cost-effectiveness studies<br>2. Be applicable across varying insurance schemes

Below we provide our argumentation for or against inclusion of the suggested outcomes in the next round of Delphi voting. If you disagree with our decision, there will be room to discuss this during our meeting on May 25.

For peer review only

Suggested outcome	Yes or No from COS team to include in next round	Reason
**Caregiver outcomes i.e. does caregiving affect work production**	No	This is an indirect variable/outcome. A contextual factor which may impact work but does not directly measure at either mental or physical level of "activity" by an individual.
**Worker satisfaction with job**	No	Job satisfaction was already included under outcome concept 2.5.1 "perceptions affecting productivity".
**Worker preceived stigma/bias from employer/society**	No	(Self) perceived stigma was already included under outcome concept 2.5.1 "perceptions affecting productivity".
**Provided with accomodation(s) for equitable work environment**	No	This is an indirect variable/outcome. A contextual factor which may impact work but does not directly measure at either mental or physical level of "activity" by an individual.
**Perceived social support - includes support from employer and access to appropriate work modifications)**	No	This is an indirect variable/outcome. A contextual factor which may impact work but does not directly measure at either mental or physical level of "activity" by an individual.
**Satisfaction of the worker with its career**	No	Job satisfaction was already included under outcome concept 2.5.1 "perceptions affecting productivity".
**Sustainable employment**	Yes	
**Work Ability Score (WAS)**	No	This is a measurement instrument which would measure the outcome 2.3.1 "work ability".
**Employment 3 - 5 years post injury/illness**	No	This would be a more specific definition of the outcome concept 3.1.4 (duration of employment).
**Accommodations requested and received**	No	This is an indirect variable/outcome. A contextual factor which may impact work but does not directly measure at either mental or physical level of "activity" by an individual.
**Quality of life**	No	Does not match criteria 4: COS for Work outcomes should be work participation specific.
**Health Status**	No	Does not match criteria 4: COS for Work outcomes should be work participation specific.

**Employer attention to occupational safety and health**	No	This is an indirect variable/outcome. A contextual factor which may impact work but does not directly measure at either mental or physical level of "activity" by an individual.
**Worker participation in job design**	No	This outcome is relevant in the context of workers participation decision making processes rather than evaluating a person's ability to work despite of a health problem.
**Worker participation in evaluation of injury control interventions**	No	This outcome is relevant in the context of workers participation decision making processes rather than evaluating a person's ability to work despite of a health problem.
**Adequacy of compensation system in replacing lost income after disability**	No	This is an indirect variable/outcome. A contextual factor which may impact work but does not directly measure at either mental or physical level of "activity" by an individual.
**Injury rate after return to work**	No	Does not match criteria 4: COS for Work outcomes should be work participation specific.
**Work Participation Score within 12 months following RTW; defined as a fraction with numbers of RTW weeks in the numerator and numbers of weeks receiving health related benefits in the denominator. K. Biering; N.H. Hjollund and T. Lund 2013**	No	This is at the level of measurement of RTW rather than on the level of outcome concepts.
**Average time from requested evaluation to determination**	No	Does not match criteria 4: COS for Work outcomes should be work participation specific.
**Possibilities to get a suitable job in the same organisation/group**	No	This would fall under the outcome concept 3.1.2 (having a pre-defined status of employment).
**Having support by your colleques - possibillitie that colleques take over task by your absence**	No	This is an indirect variable/outcome. A contextual factor which may impact work but does not directly measure at either mental or physical level of "activity" by an individual.
**Safe environment/culture in the organsiation by meaning workers**	No	This is an indirect variable/outcome. A contextual factor which may impact work but does not directly measure at either mental or physical level of "activity" by an individual.
**Number of HR coach /buddy's for returning/adjust to job in % total employees of organisations**	No	This is an indirect variable/outcome. A contextual factor which may impact work but does not directly measure at either mental or physical level of "activity" by an individual.
**Flexibility in hours and location to fulfill your tasks**	No	This is an indirect variable/outcome. A contextual factor which may impact

For peer review only

		work but does not directly measure at either mental or physical level of "activity" by an individual.
**Amount that is invested in hardware to maintain /keep workers (for instead invalid toilets rsi mouse)**	No	This is an indirect variable/outcome. A contextual factor which may impact work but does not directly measure at either mental or physical level of "activity" by an individual.
**Ability to visit a work related docter (arbocurative)**	No	This is an indirect variable/outcome. A contextual factor which may impact work but does not directly measure at either mental or physical level of "activity" by an individual.
**Work satisfaction vs normal workers**	No	Job satisfaction was already included under outcome concept 2.5.1 "perceptions affecting productivity".
**Level of education**	No	Baseline characteristic rather than an outcome measured at the start and end of an intervention to establish effectiveness.
**Sort of work (physical or mental capacity needed for the job)**	No	This is an indirect variable/outcome. A contextual factor which may impact work but does not directly measure at either mental or physical level of "activity" by an individual.
**Is the sick leave from a work related injury**	Yes/No	To be discussed
**Is there paid sick leave at the workplace**	No	This is an indirect variable/outcome. A contractual factor.
**Did workers see a company provided health care person or receive outside medical care**	No	This is an indirect variable/outcome. A contextual factor which may impact work but does not directly measure at either mental or physical level of "activity" by an individual.
**Did the person get any workers compensation for their injury**	No	This is an indirect variable/outcome. A contractual factor.
**If a work related injury; was the workplace changed to reduce chance of reinjury?**	No	This is an indirect variable/outcome. A contextual factor which may impact work but does not directly measure at either mental or physical level of "activity" by an individual.
**Hybrid work if position allows - in person and at home**	No	This is an indirect variable/outcome. A contextual factor which may impact work but does not directly measure at either mental or physical level of "activity" by an individual.

For peer review only

Results of the first round ratings combined for all stakeholders and stakeholder groups separately. Ratings are presented for each stage from most to least highly rated; a combination of mean score ($\bar{x}$) and the percentage of the stakeholders which rated an outcome with a score between 7-9. N.a.: not applicable. Stages of work participation: (1) initiating employment, (2) having employment, (3) increasing or maintaining productivity at work and (4) return to employment.

Stage	Outcome; clarifying text	Overall (n=55) $\bar{x}$; % 7-9	Researcher (n=35) $\bar{x}$; % 7-9	Employee (n=10) $\bar{x}$; % 7-9	Policy Maker (n=5) $\bar{x}$; % 7-9	Occupational Health Professional (n=4) $\bar{x}$; % 7-9	Employer (n=1) score
1	**Time to employment;** Time till getting a job	5,6; 44%	5,9; 51%	5,7; 50%	4,6; 0%	5; 25%	1 n.a.
1	**Self-efficacy for job procurement;** Self-efficacy for job procurement; Hope, optimism and self-efficacy for achieving vocational success; Social/intepersonal self-efficacy	5,6; 38%	5,4; 31%	6,1; 50%	6,2; 60%	5,8; 50%	1 n.a.
1	**Skills for job procurement;** Total work-related network, Engagement in work seeking activities, Job interview skills, Work-related benefit of vocational training	5,4; 38%	5; 29%	6,4; 60%	5,2; 40%	5,3; 50%	9 n.a.
2	**Employment - any type of employment including self-employment;** Any type of employment including self-employment	7,4; 75%	7,6; 80%	7,2; 70%	6,6; 60%	7; 75%	9 n.a.
2	**Employment - having lost**	6,5; 55%	6,4; 54%	7,5; 80%	5,4; 20%	7,3; 50%	1 n.a.

For peer review only

	**employment;** Having lost employment						
2	**Employment - predefined type of employment;** Having met a pre-defined status of employment; part/full time, competitive work in a mainstream setting, performing the same tasks as non-disabled workers	6,5; 51%	6,5; 51%	7,1; 60%	7,4; 60%	5; 25%	1 n.a.
2	**Work disability - Permanent and complete inability to engage in any work participation;** Permanent and complete inability to engage in any work participation	6,7; 34%	6,9; 63%	7,2; 80%	6; 40%	6,3; 50%	1 n.a.
2	**Work disability - Permanent partial disability to engage in work participation;** Permanent partial disability to engage in work participation	6,4; 31%	6,6; 51%	7,5; 90%	6; 40%	6; 50%	1 n.a.
2	**Employment - duration of employment;** Duration of employment	5,7; 40%	5,9; 40%	6; 50%	5,4; 20%	5; 50%	1 n.a.
2	**Employment - predefined type or amount of income;** Predefined type or amount of income	5,5; 33%	5,1; 23%	6,9; 70%	5,4; 20%	4.3; 25%	9 n.a.

3	**Work productivity loss (economic evaluation);** Overall loss of productivity at work (in terms of quality or output at work) resulting from presenteeism or absenteeism regardless of cause. Typically used for economic evaluations.	6,4; 58%	6,7; 63%	6,9; 70%	6; 40%	5; 25%	1 n.a.
3	**Work activity impairment;** Experienced functional impairment of performing work activities due to a health problem.	6,4; 51%	5,9; 40%	7,7; 80%	6; 40%	7,5; 75%	9 n.a.
3	**Work ability;** Current self-rated physical and mental work ability compared to lifetime best.	6,3; 53%	6,3; 46%	7,1; 90%	6,2; 40%	5,6; 50%	1 n.a.
3	**Presenteeism;** Not being productive while at work due to health problems. Typically used to evaluate the effect of health interventions for individuals.	5,9; 35%	5,8; 29%	6,7; 70%	4,6 ; 0%	5,3; 25%	9 n.a.
3	**Perceptions affecting productivity;** Beliefs in working capacity, motivation for work, vocational commitment, job coping, job satisfaction, need	5,5; 36%	5,1; 26%	7,2; 80%	5,2; 40%	4,3; 25%	9 n.a.

For peer review only

	for recovery, work-life balance, (self)-perceived stigma						
4	**Return to work - proportion of workers;** Proportion of workers that return to work after being absent because of illness	7,4; 76%	7,7; 80%	7; 70%	7,8; 80%	7,5; 75%	1 n.a.
4	**Return to work - time to return to work;** Time to return to work or duration of sickleave after being absent because of illness	7; 67%	7,3; 71%	6,8; 60%	6,8; 60%	7; 75%	1 n.a.
4	**Sustainable return to work – proportion of workers;** Proportion of workers that return to work and remain at work for a specified amount of time	6,7; 57%	6,3; 49%	7,6; 80%	7,2; 60%	6; 50%	9 n.a.
4	**Sustainable return to work – time until;** Time to return to work in workers absent because of illness and who do not relapse within a specified amount of time	6,3; 49%	6,1; 42%	6,9; 60%	6,6; 60%	5,8; 50%	9 n.a.
4	**Sick leave frequency;** Number of episodes of sick leave *per worker* expressed as percentage	5,8; 44%	5,4; 37%	6,9; 60%	5,6; 40%	5,5; 50%	9 n.a.
4	**Return to work - the worker's perceptions;** Self-efficacy for return	5,7; 38%	5; 20%	7,8; 80%	4,3; 20%	4,3; 25%	9 n.a.

	to work, return to work expectations or intention to return to work						
4	**Sick leave duration of all workers;** Working days or calendar days absent because of illness averaged over all workers	5,5; 38%	5,4; 37%	6,8; 50%	5; 20%	4; 25%	1 n.a.
4	**Sick leave duration - over all sick leave episodes;** Working days absent averaged over all sick leave episodes; can also be expressed as calendar days sick	5,4; 36%	5,6; 34%	6,8; 60%	4,8; 20%	3; 25%	1 n.a.
4	**Sickness absence among absent workers in an organization/group - lost working or sick days;** Working days or lost sick days as a percentage	5,3; 33%	5,3; 31%	6,1; 40%	6; 40%	4,3; 25%	1 n.a.
4	**Sickness absence among all workers;** Percentage of all working days of all workers in an organization during a time period e.g. 12 months; Sick days as percentage of all available days during a time period	5,3; 31%	5,3; 29%	6,8; 50%	6; 20%	4,3; 25%	1 n.a.

Core Outcome Set-STandards for Reporting: The COS-STAR Statement.

SECTION/TOPIC ITEM No. CHECKLIST ITEM	TOPIC ITEM No.	CHECKLIST ITEM	Reported in line numbers of the manuscript
TITLE/ABSTRACT			
Title	1	Identify in the title that the paper reports the development of a COS Abstract	1-4
Abstract	1a	Provide a structured summary	40-56
INTRODUCTION			
Background and Objectives	2a	Describe the background and explain the rationale for developing the COS	81-87
	2b	Describe the specific objectives with reference to developing a COS	81-87
Scope	3a	Describe the health condition(s) and population(s) covered by the COS	81-87
	3b	Describe the intervention(s) covered by the COS	81-87
	3c	Describe the setting(s) in which the COS is to be applied	81-87
METHODS			
Protocol/Registry Entry	4	Indicate where the COS development protocol can be accessed, if available, and/or the study registration detail	116
Participants	5	Describe the rationale for stakeholder groups involved in the COS development process, eligibility criteria for participants from each group, and a description of how the individuals involved were identified	138-146
Information Sources	6a	Describe the information sources used to identify an initial list of outcomes.	150-152
	6b	Describe how outcomes were dropped/combined, with reasons (if applicable)	213-224
Consensus Process	7	Describe how the consensus process was undertaken	261-321
Outcome Scoring	8	Describe how outcomes were scored and how scores were summarised	169-185
Consensus Definition	9a	Describe the consensus definition	213-218, 262-263
Ethics and Consent	10	Provide a statement regarding the ethics and consent issues for the study	462-466
RESULTS			
Protocol Deviations	11	Describe any changes from the protocol (if applicable), with reasons, and describe what impact these changes have on the results	Protocol was followed
Participants	12	Present data on the number and relevant characteristics of the people	234-244

For peer review only

		involved at all stages of COS development	
Outcomes	13a	List all outcomes considered at the start of the consensus process	168-172
	13b	Describe any new outcomes introduced and any outcomes dropped, with reasons, during the consensus process	260-300, 324-337
COS	14	List the outcomes in the final COS	340-341
DISCUSSION			
Limitations	15	Discuss any limitations in the COS development process	356-368
Conclusions	16	Provide an interpretation of the final COS in the context of other evidence, and implications for future research	367-398
OTHER INFORMATION			
Funding	17	Describe sources of funding/role of funders	445-447
Conflicts of Interest	18	Describe any conflicts of interest within the study team and how these were managed	453

For peer review only
